# Efficient metal-free photochemical borylation of aryl halides under batch and continuous-flow conditions†
†Electronic supplementary information (ESI) available: Experimental procedures and characterization of new compounds are provided. See DOI: 10.1039/c5sc04521e


**DOI:** 10.1039/c5sc04521e

**Published:** 2016-02-01

**Authors:** Kai Chen, Shuai Zhang, Pei He, Pengfei Li

**Affiliations:** a Center for Organic Chemistry , Frontier Institute of Science and Technology (FIST) , Xi'an Jiaotong University , 99 Yanxiang Road , Xi'an , Shaanxi 710054 , China . Email: lipengfei@mail.xjtu.edu.cn

## Abstract

A rapid, chemoselective and metal-free C–B bond-forming reaction of aryl iodides and bromides in aqueous solution at low temperatures was discovered. This reaction is amenable to batch and continuous-flow conditions and shows exceptional functional group tolerance and broad substrate scope regarding both the aryl halide and the borylating reagent. Initial mechanistic experiments indicated a photolytically generated aryl radical as the key intermediate.

## Abstract 2

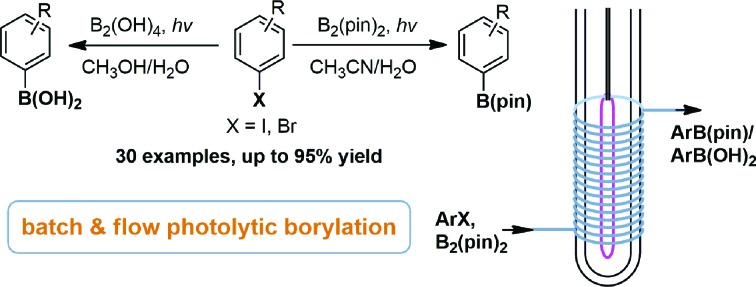
A metal-free C–B bond forming reaction of aryl halides under batch and continuous-flow conditions is described.

## Introduction

**Scheme 1:**
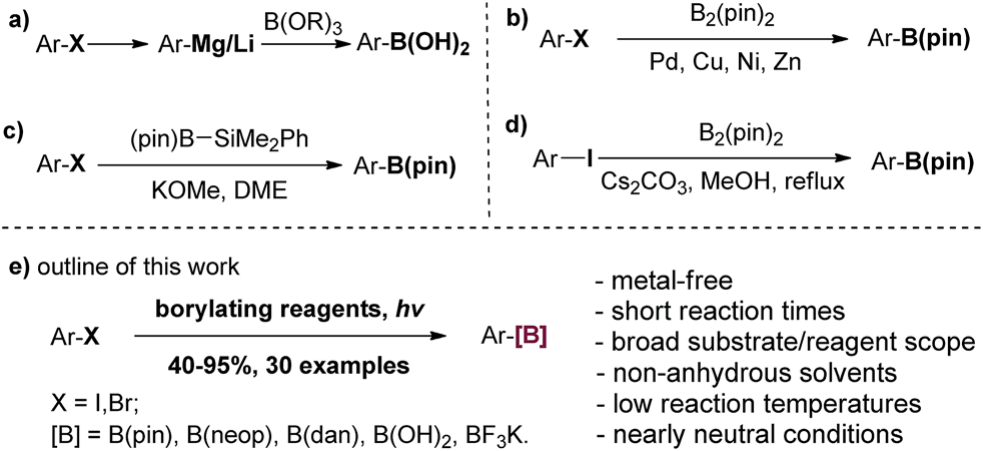
Summary of borylation reactions of aryl halides and outline of this work.

Arylboronic acids and esters have found broad applications in chemical, medicinal and materials sciences. In synthetic organic chemistry, in particular, they are versatile synthons for the formation of carbon–carbon or carbon–heteroatom bonds.^1^ Conventional methods for generating arylboron compounds involve reactions of arylmetallic intermediates with trialkyl borates, followed by transesterification or hydrolysis. These reactions suffer some major drawbacks such as limited functional group tolerance as well as the necessity of rigorous anhydrous conditions (Scheme 1a).^2^ In recent decades, transition metal-catalyzed borylation reactions using palladium, nickel, copper and zinc have emerged as highly useful methods for the conversion of C–X bonds to C–B bonds (Scheme 1b).^3^ More recently, direct C–H borylation methods based on transition-metal catalysts have also been developed.^4^ In order to reduce the costs and the amount of heavy metal residue in the final products, several transition-metal-free methods for C–B bond formation have been developed. Ito and coworkers discovered an alkali alkoxide-mediated borylation of aryl halides with a silylborane as the unique borylating reagent (Scheme 1c).^5^ Zhang and coworkers reported that aryl iodides could be borylated with 4.0 equivalents of bis(pinacolato)diboron in refluxing methanol using 2.0 equivalents of Ce_2_CO_3_ as the promoter. The reaction time ranged from several hours to days and the yields were generally moderate (Scheme 1d).^6^ Fernándes and Muñiz transformed diaryliodonium acetates to arylboronates under mild conditions.^7^ Using aryl amines as the starting material, Wang developed a mild and efficient Sandmeyer-type borylation process.8a–c Borylation of aryl diazonium salts8d–f and aryl triazenes8*g* has also been reported. In addition, innovative methods for direct C–H borylation under transition metal-free conditions have been reported,^9^ although the substrates were limited to either electron rich arenes or heterocycles, and air and moisture sensitive reagents were needed. Consequently, a practical, metal-free method that is rapid and effective, works under mild conditions with various readily available borylating reagents, shows high functional group tolerance and avoids strong acids, bases and hazardous reagents is still highly desirable. Herein, we wish to report our discovery and development of a new borylation reaction of aryl halides using light as a clean reagent (Scheme 1e).^10^

## Results and discussion

Initially, a solution of 4-iodoanisole (**1a**) and bis(pinacolato)-diboron (**2**) in acetonitrile was placed in a quartz test tube and irradiated with a 300 W high pressure mercury lamp (maximum at 365 nm) for 4 hours. Encouragingly, the desired aryl-B(pin) product **3a** was formed in 29% yield based on ^1^H NMR analysis of the crude product (Table 1, entry 1). Other polar solvents such as trifluoroethanol and methanol did not improve the reaction (entries 2 and 3). Adding water and acetone as co-solvents was beneficial in both cases and increased the yield to 46% (entries 4 and 5). Screening of various organic and inorganic additives revealed that an organic base, *N*,*N*,*N*′,*N*′-tetramethyldiaminomethane (TMDAM), could further improve the yield to 58% (entry 9). By comparison, other bases led to inferior results (entries 6–8). Interestingly, a greater amount of TMDAM led to a significantly lower yield (entry 10). Using two equivalents of B_2_(pin)_2_ could improve the yield to 72% (entry 11). Further optimization by changing the reaction concentration of **1a** resulted in a higher yield (*c* = 0.1 M, 81% yield) (entry 12 *vs.* 11 and 13).

**Table 1:** Reaction optimization under batch and continuous-flow conditions

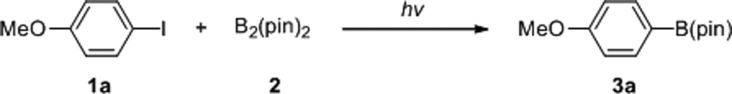				
Entry	**2** (eq.)	Solvent	Additive (mol%)	Yield^*c*^ [%]
**Batch conditions** ^*a*^				
1	1.0	MeCN	None	29
2	1.0	TFE	None	26
3	1.0	MeOH	None	15
4	1.0	MeCN/H_2_O	None	42
5	1.0	MeCN/H_2_O/acetone	None	46
6	1.0	MeCN/H_2_O/acetone	Cs_2_CO_3_ (100)	16
7	1.0	MeCN/H_2_O/acetone	KO^*t*^Bu (100)	12
8	1.0	MeCN/H_2_O/acetone	TMEDA (50)	52
9	1.0	MeCN/H_2_O/acetone	TMDAM (50)	58
10	1.0	MeCN/H_2_O/acetone	TMDAM (100)	39
11	2.0	MeCN/H_2_O/acetone	TMDAM (50)	72
**12** ^*d*^	**2.0**	**MeCN/H**_**2**_ **O/acetone**	**TMDAM (50)**	**81**
13^*e*^	2.0	MeCN/H_2_O/acetone	TMDAM (50)	55

**Flow conditions** ^*b*^				
14	2.0	MeCN/H_2_O/acetone	TMDAM (50)	87
**15**	**1.5**	**MeCN/H**_**2**_ **O/acetone**	**TMDAM (50)**	**88**

^*a*^Batch conditions: **1a** (0.1–0.2 mmol, *c* = 0.05 M/0.1 M), **2** (0.1–0.4 mmol), RT, 4 h.

^*b*^Flow conditions: **1a** (*c* = 0.1 M), –5 °C, residence time 15 min.

^*c*^Determined by ^1^H NMR with 1,3,5-trimethoxybenzene as an internal standard.

^*d*^ *c* = 0.1 M.

^*e*^ *c* = 0.2 M; TMEDA: *N*,*N*,*N*,*N*-tetramethylethylenediamine; TMDAM: *N*,*N*,*N*′,*N*′-tetramethyldiaminomethane.

During the study, we observed gradual decomposition of B_2_(pin)_2_. We felt that continuous-flow photolytic conditions might help in reducing the amount of B_2_(pin)_2_ by competitively accelerating the desired reaction. In comparison with a typical batch photoreactor, microchannel photochemical reactors have significant benefits for reaction efficiency, yield, reproducibility, material throughput and scale-up.^11^–^13^ Based on the method developed by Booker-Milburn11*a* and our own experience in flow chemistry,^14^ we designed and assembled a continuous-flow photochemical reactor. Thus, transparent fluorinated ethylene propylene (FEP) tubing (reaction volume 780 μL) was coiled around a jacketed quartz immersion well in which the mercury lamp was situated. The reaction temperature was regulated by a cooling liquid circulating pump (see ESI†). A stock solution containing all reactants and reagents was introduced into the tubing using a syringe pump. To our delight, running the reaction under the same conditions as entry 12 but in continuous-flow mode gave **3a** in excellent yield (87%, entry 14) with a residence time of only 15 minutes. Indeed, the amount of B_2_(pin)_2_ could be reduced to 1.5 equivalents without affecting the reaction efficiency (88% yield, entry 15).

With the optimized conditions in hand, we examined the substrate scope of the current borylation reaction under batch and/or continuous-flow conditions, as summarized in Table 2. Iodoarenes with various electron-donating, -neutral and -withdrawing groups at the *para*-, *meta*-, or *ortho*-positions, including hydroxyl, amino, amide, ester, acid, ketone, cyano, fluorine, boronate and trifluoromethyl groups, were all efficiently converted to the corresponding aryl pinacol boronates in good to excellent yields (**3a–3r**). Groups potentially reactive under UV light such as aryl ketone (for **3i**) and biaryl (for **3h**) were compatible. A substrate containing an allyl ether group was also viable (**3r**), which is interesting considering that the reaction might involve a reactive carbon-based radical and the double bond could be attacked. In addition, the borylation of 2-amino-5-iodopyridine was possible, and a moderate yield of the corresponding boronate **3s** was observed by ^1^H NMR spectroscopic analysis. Attempts to purify **3s** were unsuccessful due to its decomposition on silica gel. Furthermore, when aryl bromides were subjected to the same reaction conditions, the desired products were produced in comparable or slightly lower yields than the iodides (**3c**, **3f**, **3k**, **3l** and **3t–3x**). Finally, different borylating reagents were utilized under otherwise identical conditions. Reactions using bis(neopentanediolato)diboron B_2_(neop)_2_ successfully afforded the desired products in good yields (**3y** and **3z**). Interestingly, when an unsymmetrical diboron (pin)B–B(dan) was employed, selective introduction of the B(dan) moiety was realized (**3aa** and **3ab**) and no aryl pinacol boronate was observed.^15^ To demonstrate the stability and usefulness of this reaction in larger scale preparation, the borylation reactions of iodobenzene and 4-iodophenol were carried out at gram scale (10.0 mmol) employing a commercial automated flow chemistry system (reactor volume 7.8 mL, see ESI†). Without any further optimization, the reactions produced the desired arylboronate products in excellent isolated yields (**3b** 90% and **3c** 93%) and the productivity corresponded to ∼3 mmol h^–1^.

**Table 2:** Substrate scope of the photolytic borylation^*a*^

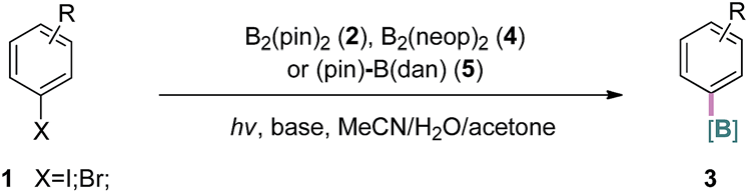
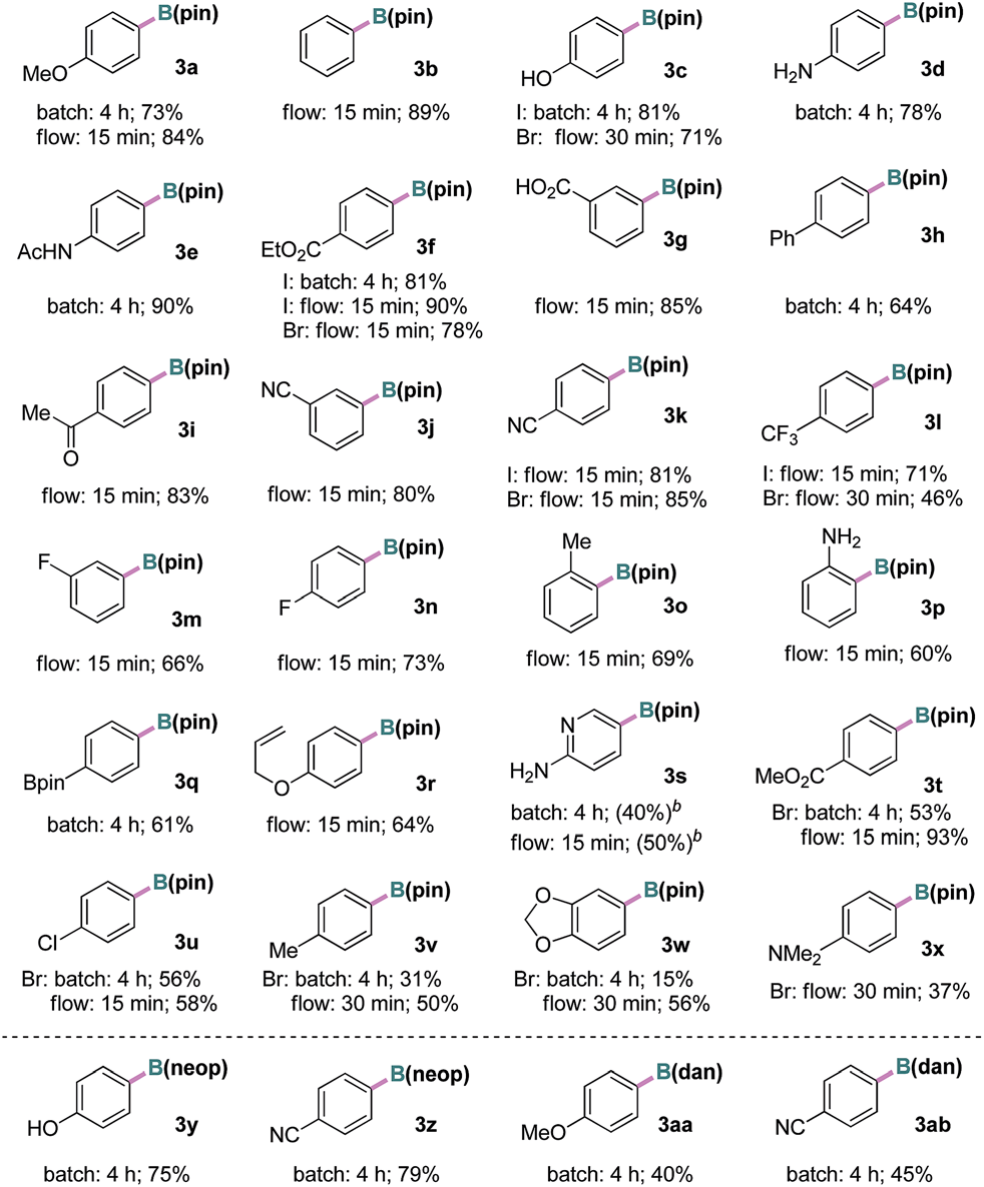

^*a*^Batch conditions: **1a** (0.2 mmol, *c* = 0.1 M), **2** (0.4 mmol, 2.0 eq.), TMDAM (0.5 eq.), RT, 4 h; flow conditions: **1a** (*c* = 0.1 M), **2** (1.5 eq.), TMDAM (0.5 eq.), –5 °C, residence time 15–30 min.

^*b*^Determined by ^1^H NMR with 1,3,5-trimethoxybenzene as an internal standard; TMDAM: *N*,*N*,*N*′,*N*′-tetramethyldiaminomethane.

Encouraged by the above results, we further investigated the possibility of using a more atom economical borylating reagent, bis-boronic acid (BBA, **6**). Largely because its polar protic properties may not be amenable to most known borylation methods, this reagent has only recently been successfully used in palladium or nickel-catalyzed Miyaura borylation by Molander and coworkers.^16^ In the present borylation, pleasingly, we were able to convert 4-iodoanisole **1a** to the corresponding boronic acid **7a** under continuous-flow conditions in quantitative yield based on ^1^H NMR analysis (residence time 10 minutes). The key variation from the previous conditions was using aqueous methanol (MeOH : H_2_O = 4 : 1 v/v) as the solvent. Due to the inconvenience of isolating the pure arylboronic acid, aqueous KHF_2_ was added and the resulting potassium aryltrifluoroborate **8a** was obtained in 93% yield. Other aryl and heteroaryl iodides and a bromide were also transformed to the boronates in good to excellent yields in this manner (Table 3).

**Table 3:** Continuous-flow photolytic borylation with B_2_(OH)_4_

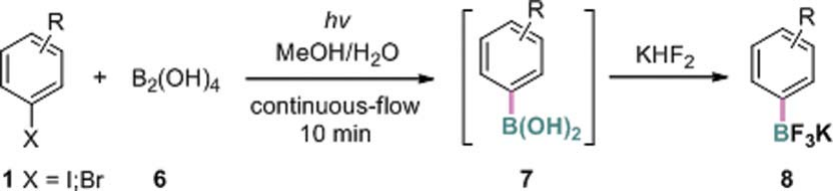
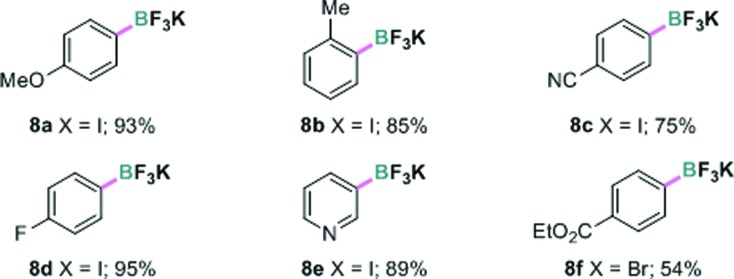

To gain insight into the reaction mechanism, and particularly to probe the role of additives and light, we conducted a series of control experiments (Table 4). When the batch reaction of **1f** with B_2_(pin)_2_ was run under the standard conditions, deiodination product **9** was formed in 7% yield in addition to the borylation product **3f** (entry 1). In the absence of both TMDAM and light (entry 2), no conversion was observed. However, the reaction with 0.5 equivalents of TMDAM in the dark led to a small amount of **3f** (entry 3); higher reaction temperatures and prolonged reaction time had little influence on the outcome. A hydrogen atom donor, Bu_3_SnH, increased the conversion but led to **9** as the major product (entry 4). Furthermore, the reaction with Bu_3_SnH under UV irradiation afforded **9** in high yield (entry 5). Similarly, using 9,10-dihydroanthracene instead of Bu_3_SnH, **9** (26%) and concomitant anthracene (11%) were observed (entry 6). Finally, when TEMPO was added as a radical scavenger, the conversion was low and four products including **3f** (15%), **9** (11%), the aryl-TEMPO adduct **10** (14%) and ethyl 4-hydroxybenzoate **11** (26%) were formed (entry 7).

**Table 4:** Control experiments for preliminary mechanistic studies^*a*^

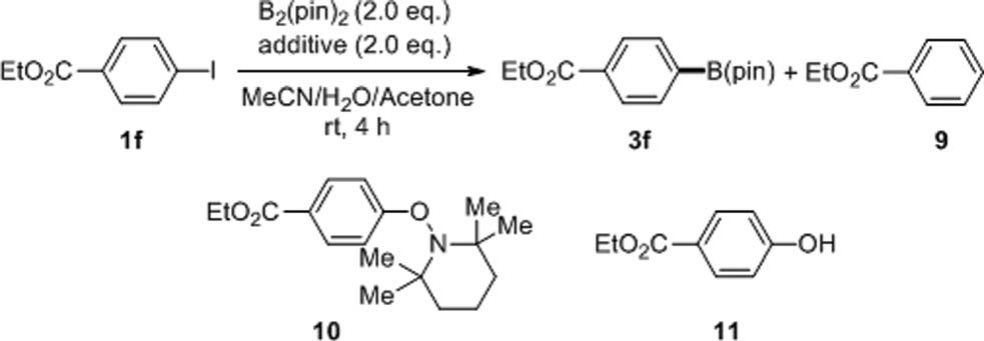						
Entry	Light	TMDAM	Additive	Conversion [%]	Yield of **3f** [%]	Yield of **9** [%]
1	+	+	–	100	81	7
2	–	–	–	0	0	0
3	–	+	–	13	13	0
4	–	+	Bu_3_SnH	46	17	26
5	+	+	Bu_3_SnH	100	18	80
6	+	+	DHA	68	42	26^*b*^
7	+	+	TEMPO	69	15	11

^*a*^Reactions were run in batch and yields were determined by ^1^H NMR spectroscopic analysis with 1,3,5-trimethoxybenzene as an internal standard.

^*b*^11% of anthracene was formed. TMDAM: *N*,*N*,*N*′,*N*′-tetramethyldiaminomethane; DHA: 9,10-dihydroanthracene; TEMPO: (2,2,6,6-tetramethylpiperidin-1-yl)oxyl.

**Scheme 2:**
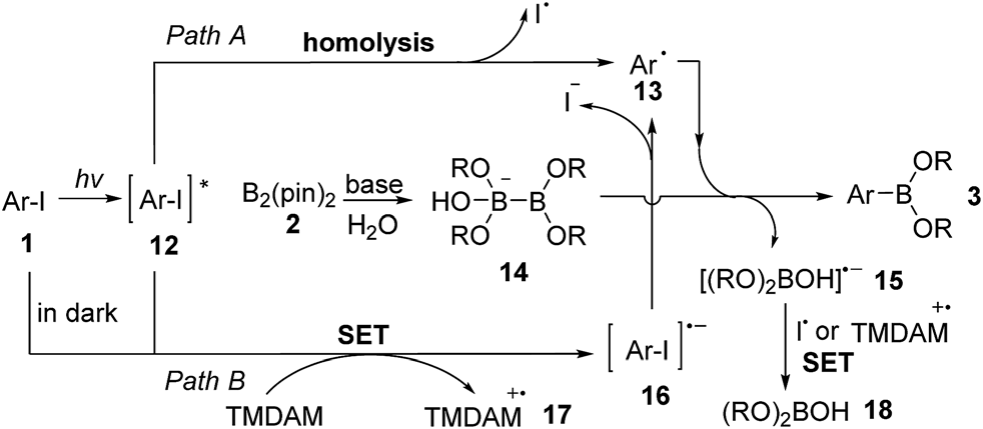
Proposed reaction mechanism.

Based on the experimental results and related reports on photolytic reactions of aryl iodides,^17^ we propose two pathways both involving an aryl radical intermediate as the possible reaction mechanism (Scheme 2). The excited state **12** is generated by UV irradiation of aryl iodide **1**. In path A, **12** undergoes homolytic C–I bond cleavage to form aryl radical **13** and an iodine atom. Under aqueous conditions, TMDAM activates a water molecule, combining with B_2_(pin)_2_ (**2**) to form a sp^3^–sp^2^ diboron species **14**.^7^,8f,^18^ Aryl radical **13** then reacts with **14** to produce arylboronate **3** and a boryl radical anion **15**.^19^**15** can also be viewed as an anionic base-stabilized boryl radical.^20^ Alternatively, in path B, the excited state **12** or the starting aryl iodide **1** (when in darkness, although with low efficiency) is reduced by TMDAM *via* a single electron transfer (SET) process to form radical anion **16** and TMDAM-derived radical cation **17**. **16** then undergoes C–I bond cleavage to generate aryl radical **13** and iodide anion. Finally, **15** is oxidized by the iodine atom from path A or TMDAM-derived radical cation **17** from path B to form borate **18** as a byproduct.

## Conclusions

In summary, we have discovered a novel and efficient photolytic borylation reaction of aryl halides using diboron reagents. This metal-free reaction features very mild conditions, short reaction times, generally high yields and broad functional group tolerance. Considering the reaction conditions, borylating reagent types and possible reaction mechanism, this work represents an important complementary approach to the existing C–B bond formation methods. Further studies on the mechanism and synthetic applications of this reaction are ongoing.

## Supplementary Material

Supplementary information
