# Supplementary material for: Efficient metal-free photochemical borylation of aryl halides under batch and continuous-flow conditions
Source: Chem Sci. 2016 Feb 1;7(6):3676–80. doi: 10.1039/c5sc04521e (PMC6008923; doi:10.1039/c5sc04521e)

Electronic Supplementary Material (ESI) for Chemical Science.
This journal is © The Royal Society of Chemistry 2016

Efficient Metal-Free Photochemical Borylation of Aryl Halides under Batch and Continuous-Flow Conditions

Kai Chen, Shuai Zhang, Pei He and Pengfei Li*

Frontier Institute of Science and Technology (FIST), Center for Organic Chemistry,
Xi'an Jiaotong University, Xi'an, Shaanxi, 710054 (China).
E-mail: lipengfei@mail.xjtu.edu.cn

Table of Contents

1. General Reagent Information……………………………………………………………S2

2. General Analytical Information……………………………………………………………S2

3. General Information for Continuous-Flow Setup……………………………………...…S2

4. Reaction optimizations……………………………………………..……………………S5

5. Experimental Procedures…………………………………………………………………S8

6. References……………………………………………………………………………...S27

7. Copies of NMR Spectra…………………………………………….……………S27

1. General Reagent Information

Commercial reagents were purchased from J&K, Energy, Sigma-Aldrich, Alfa Aesar, Acros Organics, Strem Chemicals, TCI and used as received or purified according to Purification of Common Laboratory Chemicals. MeCN, MeOH and Acetone were purchased from Acros Organics and used directly without further purification. Distilled water was degassed with sonication under vacuum and then backfilled with argon.

2. General Analytical Information

NMR spectra were measured on a Bruker Avance-400 spectrometer and chemical shifts (δ) are reported in parts per million (ppm). ^{1}H NMR spectra were recorded at 400 MHz in NMR solvents ($CDCl_3$, Acetone-d_6, DMSO-d_6) and referenced internally to corresponding solvent resonance, and ^{13}C NMR spectra were recorded at 100 MHz and referenced to corresponding solvent resonance, Carbons bearing boron substituents were generally not observed due to quadrupolar relaxation. ^{11}B NMR spectra were collected on at 128.4 MHz. ^{19}F NMR spectra were collected on at 376.5 MHz. Coupling constants are reported in Hz with multiplicities denoted as s (singlet), d (doublet), t (triplet), q (quartet), m (multiplet) and br (broad). Infrared spectra were collected on a Thermo Fisher Nicolet 6700 FT-IR spectrometer using ATR (Attenuated Total Reflectance) method. Absorption maxima (ν max) are reported in wavenumbers (cm^{-1}). Melting points were determined with MP300, a laboratory Devices Inc, chinese instrument, and are uncorrected. High resolution mass spectra (HRMS) were obtained on a Bruker Apex IV FTMS spectrometer or an Agilent 6224 LC/MS TOF spectrometer.

3. General Information for Continuous Flow Setup

General Material Information

The equipment configuration that was used for the borylation reaction is depicted in Figures S1 and S2. The dual channels syringe pump (LSP02-1B) was purchased from Baoding Longer Precision Pump Company, which are suitable for high accuracy and small flow rate liquid transferring. The equipped devices (such as: quartz immersion well, 300 W high-pressure mercury lamp and accompanying power supply etc.) were part of the photochemistry instrument XPA-7 (Figures S1) which was purchased from Xujiang electromechanical plant (Nanjing, China). The transparent fluorinated ethylene propylene (FEP) tubing, fluidic connections and the back-pressure regulator were purchased from IDEX Health and Science, formerly Upchurch Scientific. The cooling liquid circulating pump (DLSB-5/10) was purchased from Zhengzhou Changcheng Scientific Industrial and Trade Co. Ltd. The E-series flow chemistry reaction system was a product of Vapourtec, UK.

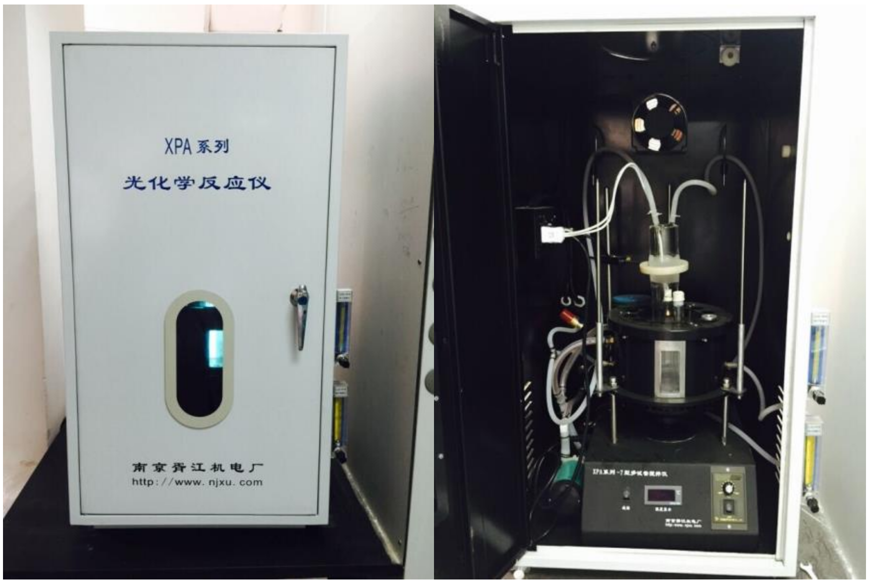

Figure S1. Photochemical batch reactor.

General setup for the continuous-flow reactor (**Figure S2**)

A 300 W high-pressure mercury lamp is positioned in the center of a jacketed quartz immersion well using the cooling liquid circulating pump to regulate the reaction temperature. One layer of the transparent fluorinated ethylene propylene (FEP) tubing 1.0 m (1.6 mm OD; 1.0 mm ID; the internal volume 0.78 mL) is winded around the central part of the immersion well, and both ends of the tubing are extended with another 0.5 m FEP tubing. The rest part of the quartz immersion well was covered with aluminum foil. The reaction mixture is introduced into the tubing using a syringe pump at controlled flow rates and collected into a cylinder after passing through a 5-psi back pressure regulator.

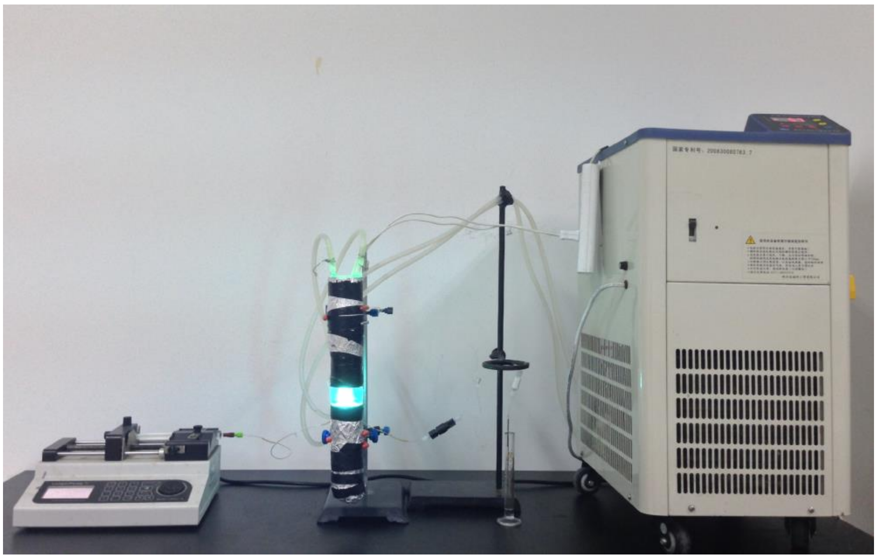

Figure S2. Schematic and pictorial photochemical flow reactor.

(Note: the UV lamp was safely placed in a box to prevent any possible injury.)

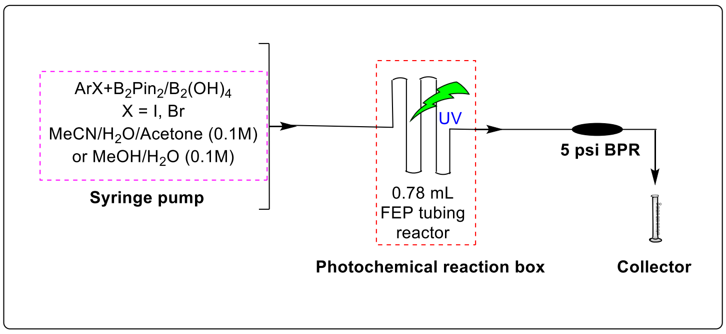

General setup for the gram-scale continuous-flow reactor

A 300 W high-pressure mercury lamp is positioned in the center of a jacketed quartz immersion well using the cooling liquid circulating pump to regulate the reaction temperature. One layer of the transparent fluorinated ethylene propylene (FEP) tubing 10.0 m (1.6 mm OD; 1.0 mm ID; the internal volume 7.8 mL) is winded around the central part of the immersion well, and both ends of the tubing are extended with another 0.5 m FEP tubing. The rest part of the quartz immersion well was covered with aluminum foil. The reaction mixture is introduced into the tubing using the Vapourtec E-series flow chemistry reactor under a 5-psi pressure at controlled flow rates and finally into the collector.

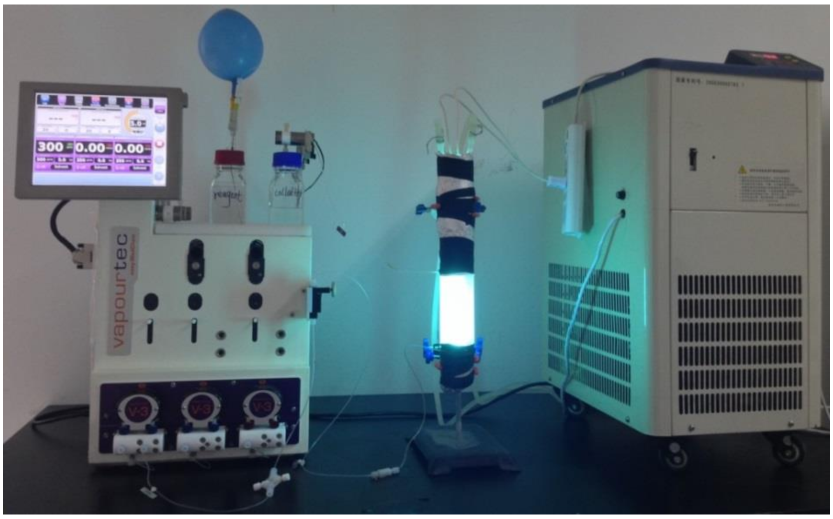

Figure S3. Gram scale photochemical flow reactor with the Vapourtec E-series.

(Note: the UV lamp was safely placed in a box to prevent any possible injury.)

4. Reaction optimization

4.1 Optimization of the reaction parameters

Table S1. Reaction optimization under batch and continuous-flow conditions.

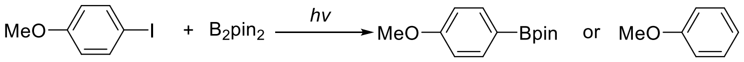

1a 2 3a 3aa

Entry	**2** (eq.)	Solvent	Additive (mol %)	Time	Yield [%][c] *(**3a**/conversion/**3aa**)*
		**Batch conditions**[a]			
1	1.0	MeCN	none	4 h	29/57/9
2	1.0	TFE	none	4 h	26/66/29
3	1.0	MeOH	none	4 h	15/70/15
4	1.0	MeCN/H_2O (4/1)	none	4 h	42/58/13
5	1.0	MeCN/H_2O (4/1) /Acetone (0.9 M)	none	4 h	46/67/11
6	1.0	MeCN/H_2O (4/1) /Acetone (0.9 M)	Cs_2CO_3 (100)	4 h	16/50/12
7	1.0	MeCN/H_2O (4/1) /Acetone (0.9 M)	KO^tBu (100)	4 h	12/25/3
8	1.0	MeCN/H_2O (4/1) /Acetone (0.9 M)	TMEDA (50)	4 h	52/71/20
9	1.0	MeCN/H_2O (4/1) /Acetone (0.9 M)	TMDAM (50)	4 h	58/93/18
10	1.0	MeCN/H_2O (4/1) /Acetone (0.9M)	TMDAM (100)	4 h	39/97/38
11	2.0	MeCN/H_2O (4/1) /Acetone (0.9 M)	TMDAM (50)	4 h	72/85/5
12	2.0	MeCN/H_2O (3/1) /Acetone (0.9M)	TMDAM (50)	4 h	56/93/19
13	2.0	MeCN/H_2O (5/1) /Acetone (0.9 M)	TMDAM (50)	4 h	53/78/14
14	2.0	MeCN/H_2O (4/1) /Acetone (1.8 M)	TMDAM (50)	4 h	58/90/23
**15**[d]	**2.0**	**MeCN/H_2O (4/1) /Acetone (0.9 M)**	**TMDAM (50)**	**4 h**	**81/94/10**
16[e]	2.0	MeCN/H_2O (4/1) /Acetone (0.9 M)	TMDAM (50)	4 h	55/85/16
17[d]	2.0	MeCN/H_2O (4/1) /Acetone (0.9 M)	TMDAM (50)	1 h	41/48/4
		**Flow conditions**[b]			
18	2.0	MeCN/H_2O (4/1) /Acetone (0.9M)	TMDAM (50)	15 min	87/100/6
**19**	**1.5**	MeCN/H_2O (4/1) /Acetone (0.9M)	**TMDAM (50)**	**15 min**	**88/100/4**

[a] batch conditions: **1a** (0.1-0.2 mmol, c = 0.05 M/0.1 M), **2** (0.1-0.4 mmol), RT, 4 h; [b] flow conditions: **1a** (c = 0.1 M), -5 ^{o}C, residence time 10-15 min; [c] determined by ^{1}H NMR with 1,3,5-trimethoxybenzene as an internal standard; [d] c = 0.1 M; [e] c = 0.2 M; f) in dark; TMEDA: N,N,N,N-tetramethylethylenediamine; TMDAM: N,N,N',N'-tetramethyldiaminomethane.

Table S2. Supplementary optimization experiments using $B_2(OH)_2$ as the borylating reagent

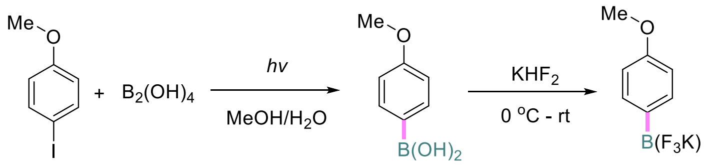

Entry	Ratio (1a/2)	Solvent	Additive [mol %]	Time	Yield [%][c]
			Batch conditions[a]		
1	1:2	MeCN	none	1 h	NR
2	1:2	MeOH	none	1 h	21
3	1:2	MeCN/H_2O/Acetone	none	1 h	NR
4	1:2	MeOH/H_2O	none	1 h	50
5	1:2	MeOH/H_2O/Acetone	TMDAM (50)	1 h	49
6[d]	1:2	MeOH/H_2O	none	1 h	67
			Flow conditions[b]		
8[d]	1:1.5	MeOH/H_2O	none	10 min	93[e]
9[d]	1:1.5	MeOH/H_2O	none	5 min	59[e]

[a] batch conditions: **1a** (0.1 mmol, c = 0.05 M), **2** (1.0-2.0 eq.), RT, 1 h; [b] flow conditions: **1a** (c = 0.1 M), **2** (1.5eq.), -5 ^{o}C; [c] determined by ^{1}H-NMR with 1,3,5-trimethoxybenzene as an internal standard; [d] batch conditions/ flow conditions: **1a** (c = 0.1 M); [e] determined by the trifluoroborate.

4.2 Studies of the decomposition of B_2pin_2

In order to reduce the amount of B_2pin_2 needed, we investigated the decomposition of B_2pin_2 under different reaction conditions and checked the results by ^{11}B NMR. We found that although a solution of B_2pin_2 in MeCN/water/acetone itself did not decompose (not shown), heating this mixture, addition of TMDAM or applying UV irradiation led to the formation of a new peak on ^{11}B NMR. The detailed results are shown in Figure S4. Using methanol-d4 as the solvent, we knew that the decomposition product was not $B_2(OH)_4$ (Figure S5).

Figure S4. ^{11}B NMR spectra of B_2pin_2 and its decomposition mixtures (in $CDCl_3$, 400 MHz)

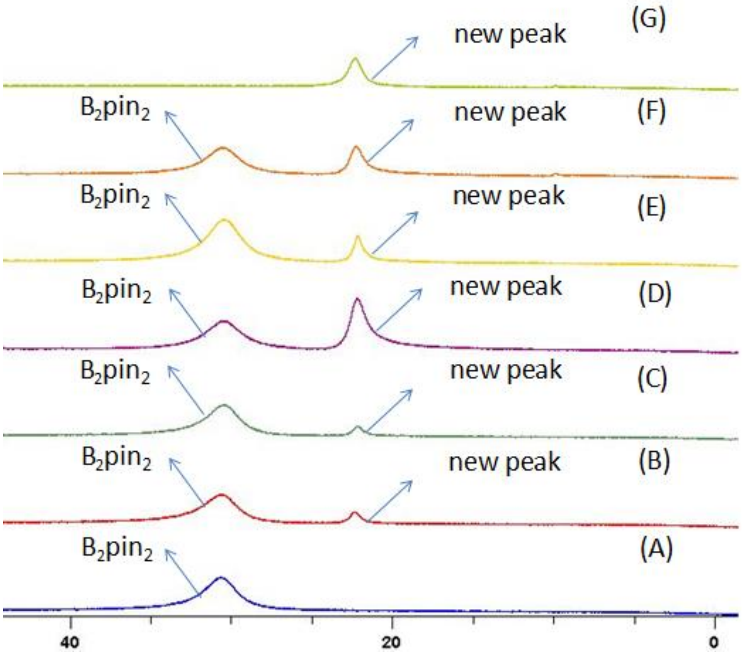

Common reaction conditions: B_2Pin_2, 0.2 mmol in MeCN/H_2O/Acetone: 2 ml

(A) blank

(B) in dark, room temperature, TMDAM 0.5 eq., 14 hours

(C) in dark, no TMDAM, 70 ^oC, 14 hours

(D) in dark, TMDAM 0.5 eq., 70 ^oC, 14 hours

(E) UV, room temperature, no TMDAM, 1 hour

(F) UV, room temperature, TMDAM 0.5 eq., 1 hour

(G) UV, room temperature, TMDAM 0.5 eq., 3 hour

Figure S5. ^{11}B NMR spectra of B_2pin_2 , $B_2(OH)_4$ and the the B_2pin_2 hydrolysis mixture in CD_3OD:

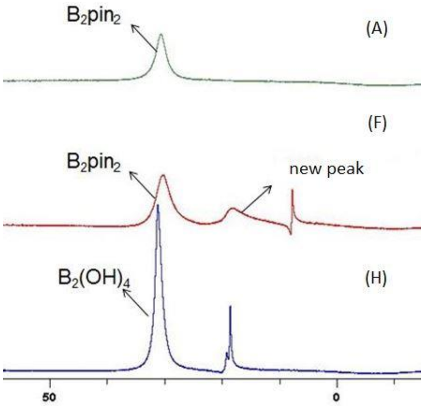

(A) B_2Pin_2, blank; (F) B_2Pin_2, UV, room temperature, TMDAM 0.5 eq., 1 hour; (H) $B_2(OH)_4$

4.3 Additional experiments in the mechanistic study

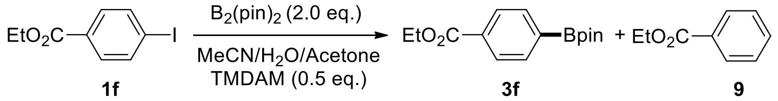

1f **3f** **9**

a) batch conditions, dark, rt, 14 hours; conversion 24%; **3f**: 24%; **9**: 0%

b) batch conditions, dark, 70 ^{o}C, 14 hours; conversion 44%; **3f**: 32%; **9**: 8%

c) batch conditions, UV, rt, 4 hours; conversion 100%; **3f**: 81%; **9**: 7%

d) flow conditions, UV, -5 ^{o}C, 15 min; conversion 100%; **3f**: 100%

Yields based on ^{1}H NMR analysis of the crude products with 1,3,5-trimethoxybenzene added as an internal standard.

5. Typical Experimental Procedures

A）Typical batch procedure of the metal-free borylation using the set-up in Figure S1

A solution of the aryl iodide (0.1-0.2 mmol), B_2pin_2/$B_2(OH)_2$ (0.1-0.4 mmol), TMDAM (if needed, 14 μL, 0.5 eq), acetone (if needed, 0.2 mL) and the additive reagents in MeCN/MeOH and H_2O (4/1 v/v, 2.0 mL) was added into a quartz test tube containing a magnetic stirring bar and the mixture was purged with argon for 10 min. The tube was then capped with a septum. The reaction mixture was irradiated using a 300 W high-pressure mercury lamp through a water-cooled quartz immersion well for 4h. Then the internal standard 1,3,5-trimethoxybenzene (0.1-0.2 mmol) was added into the crude product mixture for the ^{1}HNMR yield study. Or through the usual workup and column chromatography produced the target boronate.

B）Typical procedure for the synthesis of boronate esters in flow using the set-up in Figure S2

An oven-dried screwcapping volumetric flask (10.0 mL) was charged with aryl halide **3** (1.0 mmol, if solid, 1.0 eq) and B_2pin_2 (1.5 mmol, 1.5 eq) then capped with a septum. The vessel was evacuated and back-filled with argon (this process was carried out a total of 3 times). **3** (1.0 mmol, if liquid, 1 eq), TMDAM (70 μL, 0.5 eq) acetone (0.9 mL) and H_2O (1.8 mL) were added by syringes and MeCN was added to dissolve the solids and filled up to volume. Before reaction all the solutions were prepared under argon atmosphere and the reactors tubing were purged with MeCN three times at least. After that, all reactors and connecting tubings were filled with MeCN. A 10 mL disposable syringe was used to pump the reaction solution through the continuous-flow reactor at controlled flow rates. A cooling liquid circulating pump is used to maintain the temperature around the tubing is -5 ^{o}C. After reaching steady state, a sample of the reaction mixture was collected which contained theoretical yield of 0.3 mmol based on flow rate and collecting time. The mixture was concentrated and usual workup and column chromatography produced the target boronate.

2-(4-methoxyphenyl)-4,4,5,5-tetramethyl-1,3,2-dioxaborolane (3a).

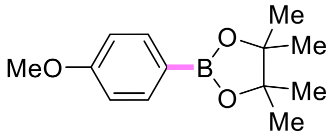

[1] Following the general procedure for batch metal-free borylation using the set-up in **Figure S1**, a solution of the 1-iodo-4-methoxybenzene (46.8 mg, 0.2 mmol), B_2pin_2 (101.6 mg, 0.4 mmol), TMDAM (14 μL, 0.1 mmol), acetone (0.2 mL) and the additive reagents in MeCN/H_2O (4/1 v/v, 2.0 mL) was added into a quartz test tube containing a magnetic stirring bar and the mixture was purged with argon for 10 min. The tube was then capped with a septum. The reaction mixture was irradiated using a 300 W high-pressure mercury lamp through a water-cooled quartz immersion well for 4h. Then 0.5 mL brine was added, the mixture was extracted with ethyl acetate. The combined organic layer were dried with sodium sulfate, filtered and concentrated in vacuo then purified by column chromatography (EA/PE 1:50) to get the product **3a** (34.2 mg, 73%) as a pale yellow liquid.

[2] Following the general continuous metal-free borylation procedure using the set-up in **Figure S2**, syringe was loaded with a solution of 1-iodo-4-methoxybenzene (234.0 mg, 1.0 mmol), B_2pin_2 (381.0 mg, 1.5 mmol), and TMDAM (70 μL, 0.5 mmol) in 10.0 mL volume. The flow rate was 52 μL/min. After steady state, a sample solution was collected into a graduated cylinder for 58 min (3.0 mL, 0.3 mmol). Then 0.5 mL brine was added, the mixture was extracted with ethyl acetate. The combined organic layer were dried with sodium sulfate, filtered and concentrated in vacuo then purified by column chromatography (EA/PE 1:50) to get the product **3a** (59.0 mg, 84%) as a pale yellow liquid. Spectroscopical data in accordance with the literature.[1] ^{1}H NMR (400 MHz, $CDCl_3$): δ ppm 7.76 (d, J = 8.4 Hz, 2H), 6.91 (d, J = 8.8 Hz, 2H), 3.83 (s, 3H), 1.33 (s, 12H). ^{13}C NMR (100 MHz, $CDCl_3$): δ ppm 162.3, 137.0, 113.4, 83.5, 55.2, 25.0. Carbon bearing boron not observed. IR (neat, cm^{-1}): 2978, 1605, 1360, 1143, 1091, 1030.

4,4,5,5-tetramethyl-2-phenyl-1,3,2-dioxaborolane (3b).

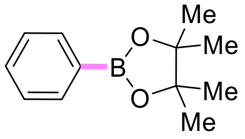

[1] Following the general continuous-flow borylation procedure using the set-up in **Figure S2**, the syringe was loaded with a solution of iodobenzene (204.0 mg, 1.0 mmol), B_2pin_2 (381.0 mg, 1.5 mmol), and TMDAM (70 μL, 0.5 mmol) in 10.0 mL volume. The flow rate was 52 μL/min. After steady state, a sample solution was collected into a graduated cylinder for 58 min (3.0 mL, 0.3 mmol). Then 0.5 mL brine was added, the mixture was extracted with ethyl acetate. The combined organic layer were dried with sodium sulfate, filtered and concentrated in vacuo then purified by column chromatography (PE) to get the product **3b** (54.5 mg, 89%) as a pale yellow liquid.

[2] The grams scale borylation of iodobenzene using the continuous-flow set-up in **Figure S3**, Vapourtec E-series flow chemistry reactor was loaded with a solution of iodobenzene (2.04 g, 10.0 mmol), B_2pin_2 (3.81 g, 15.0 mmol), TMDAM (700 μL, 5 mmol) in 100.0 mL MeCN/H_2O = 4/1 and acetone. The flow rate was 520 μL/min. After steady state, a sample solution was collected into the collector for 154 min (80.0 mL, 8.0 mmol). Then the mixture was concentrated in vacuo and extracted with ethyl acetate. The combined organic layer were dried with sodium sulfate,

filtered and concentrated in vacuo then purified by column chromatography (PE) to get the product **3b** (1.47 g, 90%) as a pale yellow liquid. Spectroscopical data was in accordance with the literature.[2] ^{1}H NMR (400 MHz, $CDCl_3$): δ ppm 7.81 (d, *J* = 7.6 Hz, 2H), 7.48 (t, *J* = 7.4 Hz, 1H), 7.38 (t, *J* = 7.4 Hz, 2H), 1.35 (s, 12H). ^{13}C NMR (100 MHz, $CDCl_3$): δ ppm 134.7, 131.2, 127.6, 83.7, 24.5. Carbon bearing boron not observed. IR (neat, cm^{-1}): 2959, 2359, 1734, 1361, 1261, 1092, 1020.

4-(4,4,5,5-tetramethyl-1,3,2-dioxaborolan-2-yl)phenol (3c).

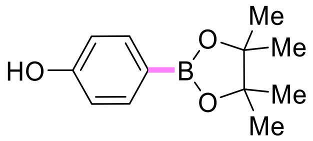

[1] Following the general procedure for batch metal-free borylation using the set-up in **Figure S1**, a solution of the 4-iodophenol (44.0 mg, 0.2 mmol), B_2pin_2 (101.6 mg, 0.4 mmol), TMDAM (14 μL, 0.5 eq), acetone (0.2 mL) and the additive reagents in $MeCN/H_2O$ (4/1 v/v, 2.0 mL) was added into a quartz test tube containing a magnetic stirring bar and the mixture was purged with argon for 10 min. The tube was then capped with a septum. The reaction mixture was irradiated using a 300 W high-pressure mercury lamp through a water-cooled quartz immersion well for 4h. Then 0.5 mL brine was added, the mixture was extracted with ethyl acetate. The combined organic layer were dried with sodium sulfate, filtered and concentrated in vacuo then purified by column chromatography (EA/PE 1:50→EA/PE 1:10) to get the product **3c** (35.6 mg, 81%) as a white solid.

[2] Following the general continuous metal-free borylation procedure using the set-up in **Figure S2**, syringe was loaded with a solution of 4-bromophenol (173.0 mg, 1.0 mmol), B_2pin_2 (381.0 mg, 1.5 mmol), and TMDAM (70 μL, 0.5 mmol) in 10.0 mL volume. The flow rate was 26 μL/min. After steady state, a sample solution was collected into a graduated cylinder for 116 min (3.0 mL, 0.3 mmol). Then 0.5 mL brine was added, the mixture was extracted with ethyl acetate. The combined organic layer were dried with sodium sulfate, filtered and concentrated in vacuo then purified by column chromatography (EA/PE 1:50→EA/PE 1:10) to get the product **3c** (46.8 mg, 71%) as a white solid.

[3] The grams scale borylation of 4-iodophenol using the continuous-flow set-up in **Figure S3**, Vapourtec E-series flow chemistry reactor was loaded with a solution of 4-iodophenol (2.20 g, 10.0 mmol), B_2pin_2 (3.81 g, 15.0 mmol), TMDAM (700 μL, 5 mmol) in 100.0 mL $MeCN/H_2O$ = 4/1 and acetone. The flow rate was 520 μL/min. After steady state, a sample solution was collected into the collector for 154 min (80.0 mL, 8.0 mmol). Then the mixture was concentrated in vacuo and extracted with ethyl acetate. The combined organic layer were dried with sodium sulfate, filtered and concentrated in vacuo then purified by column chromatography (EA/PE 1:50→EA/PE 1:10) to get the product **3c** (1.63 g, 93%) as a white solid. Mp108 - 110 ^{o}C. Spectroscopical data was in accordance with the literature.[3] ^{1}H NMR (400 MHz, $CDCl_3$): δ ppm 7.71 (d, *J* = 7.6 Hz, 2H), 6.82 (d, *J* = 7.2 Hz, 2H), 5.14 (s, 1H), 1.33 (s, 12H). ^{13}C NMR (100 MHz, $CDCl_3$): δ ppm 158.1, 137.2, 114.8, 83.6, 24.7. Carbon bearing boron not observed. IR (neat, cm-1): 3367, 2979, 1608, 1360, 1143, 1087.

4-(4,4,5,5-tetramethyl-1,3,2-dioxaborolan-2-yl)aniline (3d).

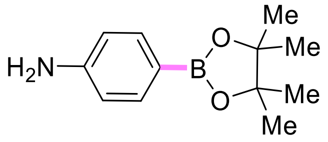

Following the general procedure for batch metal-free borylation using the set-up in **Figure S1**, a solution of the 4-iodoaniline (43.8 mg, 0.2 mmol), B_2pin_2 (101.6 mg, 0.4 mmol), TMDAM (14 μL, 0.5 eq), acetone (0.2 mL) and the additive reagents in MeCN/H_2O (4/1 v/v, 2.0 mL) was added into a quartz test tube containing a magnetic stirring bar and the mixture was purged with argon for 10 min. The tube was then capped with a septum. The reaction mixture was irradiated using a 300 W high-pressure mercury lamp through a water-cooled quartz immersion well for 4h. Then 0.5 mL brine was added, the mixture was extracted with ethyl acetate. The combined organic layer were dried with sodium sulfate, filtered and concentrated in vacuo then purified by column chromatography (EA/PE 1:50→EA/PE 1:5) to get the product **3d** (34.2 mg, 78%) as a white solid. Mp134 - 136 ^{o}C. Spectroscopical data in accordance with the literature.[3] ^{1}H NMR (400 MHz, $CDCl_3$): δ ppm 7.62(d, *J* = 7.6 Hz, 2H), 6.66 (d, *J* = 8.0 Hz, 2H), 3.82 (s, 2H), 1.32 (s, 12H). ^{13}C NMR (100 MHz, $CDCl_3$): δ ppm 149.2, 136.5, 114.1, 83.3, 25.0.Carbon bearing boron not observed. IR (neat, cm-1): 3450, 2977, 1603, 1360, 1143, 1087.

N-(4-(4,4,5,5-tetramethyl-1,3,2-dioxaborolan-2-yl)phenyl)acetamide (3e).

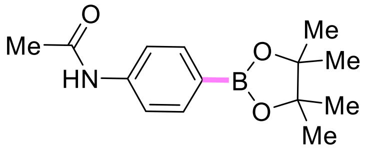

Following the general procedure for batch metal-free borylation using the set-up in **Figure S1**, syringe was loaded with a solution of N-(4-iodophenyl)acetamide (52.2 mg, 0.2 mmol), B_2pin_2 (101.6 mg, 0.4 mmol), TMDAM (14 μL, 0.5 eq), acetone (0.2 mL) and the additive reagents in MeCN/H_2O (4/1 v/v, 2.0 mL) was added into a quartz test tube containing a magnetic stirring bar and the mixture was purged with argon for 10 min. The tube was then capped with a septum. The reaction mixture was irradiated using a 300 W high-pressure mercury lamp through a water-cooled quartz immersion well for 4h. Then 0.5 mL brine was added, the mixture was extracted with ethyl acetate. The combined organic layer were dried with sodium sulfate, filtered and concentrated in vacuo then purified by column chromatography (EA/PE 1:2) to get the product **3e** (47.0 mg, 90%) as a white solid. Mp188 - 190 ^{o}C. Spectroscopical data in accordance with the literature.[4] ^{1}H NMR (400 MHz, $CDCl_3$): δ ppm 7.77 (d, *J* = 8.4 Hz, 2H), 7.51(d, *J* = 8.0 Hz, 2H), 7.24 (s, 1H), 2.18 (s, 3H), 1.33 (s, 12H). ^{13}C NMR (100 MHz, $CDCl_3$): δ ppm 168.2, 140.5, 135.8, 118.5, 83.7, 29.7, 24.8. Carbon bearing boron not observed. IR (neat, cm-1): 3309, 2980, 1595, 1362, 1145, 1091.

ethyl 4-(4,4,5,5-tetramethyl-1,3,2-dioxaborolan-2-yl)benzoate (3f).

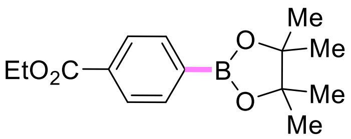

[1] Following the general procedure for batch metal-free borylation using the set-up in **Figure S1**, a solution of the ethyl 4-iodobenzoate (55.2 mg, 0.2 mmol), B_2pin_2 (101.6 mg, 0.4 mmol), TMDAM (14 μL, 0.5 eq), acetone (0.2 mL) and the additive reagents in MeCN/H_2O (4/1 v/v, 2.0 mL) was added into a quartz test tube containing a magnetic stirring bar and the mixture was purged with argon for 10 min. The tube was then capped with a septum. The reaction mixture was irradiated using a 300 W high-pressure mercury lamp through a water-cooled quartz immersion well for 4h. Then 0.5 mL brine was added, the mixture was extracted with ethyl acetate. The combined organic layer were dried with sodium sulfate, filtered and concentrated in vacuo then purified by column chromatography (EA/PE 1:300) to get the product **3f** (44.7 mg, 81%) as a pale yellow liquid.

[2] Following the general continuous metal-free borylation procedure using the set-up in **Figure S2**, syringe was loaded with a solution of ethyl 4-iodobenzoate (276.1 mg, 1.0 mmol), B_2pin_2 (381.0 mg, 1.5 mmol), and TMDAM (70 μL, 0.5 mmol) in 10.0 mL volume. The flow rate was 52 μL/min. After steady state, a sample solution was collected into a graduated cylinder for 58 min (3.0 mL, 0.3 mmol). Then 0.5 mL brine was added, the mixture was extracted with ethyl acetate. The combined organic layer were dried with sodium sulfate, filtered and concentrated in vacuo then purified by column chromatography (EA/PE 1:300) to get the product **3f** (74.5 mg, 90%) as a pale yellow liquid.

[3] Following the general continuous metal-free borylation procedure using the set-up in **Figure S2**, syringe was loaded with a solution of ethyl 4-bromobenzoate (229.1 mg, 1.0 mmol), B_2pin_2 (381.0 mg, 1.5 mmol), and TMDAM (70 μL, 0.5 mmol) in 10.0 mL volume. The flow rate was 52 μL/min. After steady state, a sample solution was collected into a graduated cylinder for 58 min (3.0 mL, 0.3 mmol). Then 0.5 mL brine was added, the mixture was extracted with ethyl acetate. The combined organic layer were dried with sodium sulfate, filtered and concentrated in vacuo then purified by column chromatography (EA/PE 1:300) to get the product **3f** (64.6 mg, 78%) as a pale yellow liquid. Spectroscopical data in accordance with the literature.[1] ^{1}H NMR (400 MHz, $CDCl_3$): δ ppm 8.03 (d, *J* = 8.0 Hz, 2H), 7.87 (d, *J* = 8.0 Hz, 2H), 4.40 (m, 2H), 1.42 (t, *J* = 7.0 Hz, 3H), 1.35 (s, 12H). ^{13}C NMR (100 MHz, $CDCl_3$): δ ppm 166.6, 134.6, 132.6, 128.5, 84.1, 61.0, 24.8, 14.3. Carbon bearing boron not observed. IR (neat, cm^{-1}): 2979, 2928, 1719, 1508, 1399, 1360, 1269, 1109.

3-(4,4,5,5-tetramethyl-1,3,2-dioxaborolan-2-yl)benzoic acid (3g).

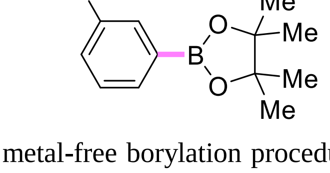

Following the general continuous metal-free borylation procedure using the set-up in **Figure S2**, syringe was loaded with a solution of 3-iodobenzoic acid (248.0 mg, 1.0 mmol), B_2pin_2 (381.0 mg, 1.5 mmol), and TMDAM (70 μL, 0.5 mmol) in 10.0 mL volume. The flow rate was 52 μL/min. After steady state, a sample solution was collected into a graduated cylinder for 58 min (3.0 mL,

0.3 mmol). Then 0.5 mL brine was added, the mixture was extracted with ethyl acetate. The combined organic layer were dried with sodium sulfate, filtered and concentrated in vacuo then purified by column chromatography (DCM/MeOH=30/1) to get the product **3g** (63.3 mg, 85%) as a white solide. Mp: 201 - 203 °C. Spectroscopical data in accordance with the literature.[12] ^{1}H NMR (400 MHz, $CDCl_3$): δ ppm 8.57 (s, 1H), 8.21 (d, *J* = 8.0 Hz, 1H), 8.05 (d, *J* = 7.2 Hz, 1H), 7.50 (t, *J* = 7.6 Hz, 1H), 1.37 (s, 12H). ^{13}C NMR (100 MHz, $CDCl_3$): δ ppm 172.2, 139.9, 136.3, 132.8, 128.9, 128.1, 84.1, 24.8. Carbon bearing boron not observed. HRMS (ESI^+): Calculated for $C_{13}H_{17}BO_4Na$ $(M+Na)^+$: 271.1117, Found: 271.1112. IR (neat, cm^{-1}): 3750, 2978, 2927, 1683, 1606, 1360, 1291, 1143.

2-([1,1'-biphenyl]-4-yl)-4,4,5,5-tetramethyl-1,3,2-dioxaborolane (3h).

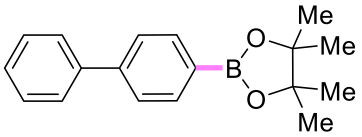

Following the general procedure for batch metal-free borylation using the set-up in **Figure S1**, a solution of the 4-iodo-1,1'-biphenyl (56.0 mg, 0.2 mmol), B_2pin_2 (101.6 mg, 0.4 mmol), TMDAM (14 μL, 0.5 eq), acetone (0.2 mL) and the additive reagents in $MeCN/H_2O$ (4/1 v/v, 2.0 mL) was added into a quartz test tube containing a magnetic stirring bar and the mixture was purged with argon for 10 min. The tube was then capped with a septum. The reaction mixture was irradiated using a 300 W high-pressure mercury lamp through a water-cooled quartz immersion well for 4h. Then 0.5 mL brine was added, the mixture was extracted with ethyl acetate. The combined organic layer were dried with sodium sulfate, filtered and concentrated in vacuo then purified by column chromatography (EA/PE 1:100) to get the product **3h** (35.8 mg, 64%) as a white solid. Mp: 103 - 105 °C. Spectroscopical data in accordance with the literature.[1] ^{1}H NMR (400 MHz, $CDCl_3$): δ ppm 7.91 (d, *J* = 8.4 Hz, 2H), 7.64 (m, 4H), 7.47 (t, *J* = 7.6 Hz, 2H), 7.38 (t, *J* = 7.4 Hz, 1H), 1.37 (s, 12H). ^{13}C NMR (100 MHz, $CDCl_3$): δ ppm 143.8, 140.9, 135.2, 128.7, 127.5, 127.2, 126.4, 83.8, 25.0. Carbon bearing boron not observed. IR (neat, cm^{-1}): 2978, 2927, 1741, 1609, 1362, 1143, 1093.

1-(4-(4,4,5,5-tetramethyl-1,3,2-dioxaborolan-2-yl)phenyl)ethan-1-one (3i).

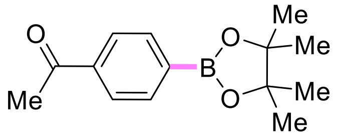

Following the general continuous metal-free borylation procedure using the set-up in **Figure S2**, syringe was loaded with a solution of 1-(4-iodophenyl)ethan-1-one (246.0 mg, 1.0 mmol), B_2pin_2 (381.0 mg, 1.5 mmol), and TMDAM (70 μL, 0.5 mmol) in 10.0 mL volume. The flow rate was 52 μL/min. After steady state, a sample solution was collected into a graduated cylinder for 58 min (3.0 mL, 0.3 mmol). Then 0.5 mL brine was added, the mixture was extracted with ethyl acetate. The combined organic layer were dried with sodium sulfate, filtered and concentrated in vacuo then purified by column chromatography (PE) to get the product **3i** (61.3 mg, 83%) as a white solid. Mp: 63 - 65 °C. Spectroscopical data in accordance with the literature.[5] ^{1}H NMR (400 MHz,

$CDCl_3$): δ ppm 7.94 (d, J = 8.0 Hz, 2H), 7.90 (d, J = 8.0 Hz, 2H), 2.62 (s, 3H), 1.36 (s, 12H). ^{13}C NMR (100 MHz, $CDCl_3$): δ ppm 198.5, 139.2, 135.0, 127.3, 84.2, 26.9, 24.9. Carbon bearing boron not observed. IR (neat, cm^{-1}): 2979, 2925, 1684, 1507, 1361, 1267, 1144, 1095.

3-(4,4,5,5-tetramethyl-1,3,2-dioxaborolan-2-yl)benzonitrile (3j).

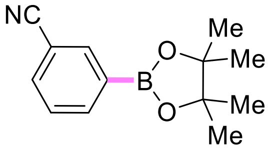

Following the general continuous metal-free borylation procedure using the set-up in **Figure S2**, syringe was loaded with a solution of 3-iodobenzonitrile (229.0 mg, 1.0 mmol), B_2pin_2 (381.0 mg, 1.5 mmol), and TMDAM (70 μL, 0.5 mmol) in 10.0 mL volume. The flow rate was 52 μL/min. After steady state, a sample solution was collected into a graduated cylinder for 58 min (3.0 mL, 0.3 mmol). Then 0.5 mL brine was added, the mixture was extracted with ethyl acetate. The combined organic layer were dried with sodium sulfate, filtered and concentrated in vacuo then purified by column chromatography (PE) to get the product **3j** (55.0 mg, 80%) as a pale yellow solide. Mp: 74 - 76 ^{o}C. Spectroscopical data in accordance with the literature. [4] ^{1}H NMR (400 MHz, $CDCl_3$): δ ppm 8.09 (s, 1H), 8.01 (d, J = 7.6 Hz, 1H), 7.73 (d, J = 9.6 Hz, 1H), 7.48 (t, J = 7.6 Hz, 1H), 1.35 (s, 12H). ^{13}C NMR (100 MHz, $CDCl_3$): δ ppm 138.7, 138.4, 134.4, 128.4, 118.8, 112.0, 84.4, 24.8. Carbon bearing boron not observed. IR (neat, cm^{-1}): 2980, 2929, 2229, 1740, 1602, 1359, 1143, 1088.

4-(4,4,5,5-tetramethyl-1,3,2-dioxaborolan-2-yl)benzonitrile (3k).

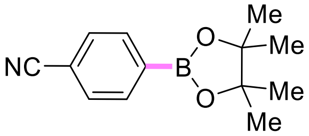

[1] Following the general continuous metal-free borylation procedure using the set-up in **Figure S2**, syringe was loaded with a solution of 4-iodobenzonitrile (229.0 mg, 1.0 mmol), B_2pin_2 (381.0 mg, 1.5 mmol), and TMDAM (70 μL, 0.5 mmol) in 10.0 mL volume. The flow rate was 52 μL/min. After steady state, a sample solution was collected into a graduated cylinder for 58 min (3.0 mL, 0.3 mmol). Then 0.5 mL brine was added, the mixture was extracted with ethyl acetate. The combined organic layer were dried with sodium sulfate, filtered and concentrated in vacuo then purified by column chromatography (PE/EA=20/1) to get the product **3k** (55.6 mg, 81%) as a pale yellow solide. Spectroscopical data in accordance with the literature.

[2] Following the general continuous metal-free borylation procedure using the set-up in **Figure S2**, syringe was loaded with a solution of 4-bromobenzonitrile (182.0 mg, 1.0 mmol), B_2pin_2 (381.0 mg, 1.5 mmol), and TMDAM (70 μL, 0.5 mmol) in 10.0 mL volume. The flow rate was 52 μL/min. After steady state, a sample solution was collected into a graduated cylinder for 58 min (3.0 mL, 0.3 mmol). Then 0.5 mL brine was added, the mixture was extracted with ethyl acetate. The combined organic layer were dried with sodium sulfate, filtered and concentrated in vacuo then purified by column chromatography (PE/EA=20/1) to get the product **3k** (58.4 mg, 85%) as a pale yellow solide. Mp: 94 - 97 ^{o}C. Spectroscopical data in accordance with the literature. [4] ^{1}H

NMR (400 MHz, $CDCl_3$): δ ppm 7.89 (d, *J* = 8.4 Hz, 2H), 7.64 (d, *J* = 8.4 Hz, 2H), 1.35 (s, 12H). ^{13}C NMR (100 MHz, $CDCl_3$): δ ppm 135.1, 131.1, 118.8, 114.5, 84.4, 24.8. Carbon bearing boron not observed. IR (neat, cm^{-1}): 2981, 2926, 2854, 2227, 1506, 1358, 1270, 1141, 1086.

4,4,5,5-tetramethyl-2-(4-(trifluoromethyl)phenyl)-1,3,2-dioxaborolane (3l).

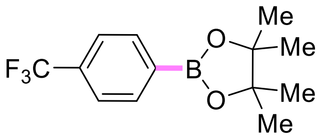

Following the general continuous metal-free borylation procedure using the set-up in **Figure S2**, syringe was loaded with a solution of 1-iodo-4-(trifluoromethyl)benzene (272.0 mg, 1.0 mmol), B_2pin_2 (381.0 mg, 1.5 mmol), and TMDAM (70 μL, 0.5 mmol) in 10.0 mL volume. The flow rate was 52 μL/min. After steady state, a sample solution was collected into a graduated cylinder for 58 min (3.0 mL, 0.3 mmol). Then 0.5 mL brine was added, the mixture was extracted with ethyl acetate. The combined organic layer were dried with sodium sulfate, filtered and concentrated in vacuo then purified by column chromatography (PE) to get the product **3l** (58.0 mg, 71%) as a white solide.

[2] Following the general continuous metal-free borylation procedure using the set-up in **Figure S2**, syringe was loaded with a solution of 1-bromo-4-(trifluoromethyl)benzene (225.0 mg, 1.0 mmol), B_2pin_2 (381.0 mg, 1.5 mmol), and TMDAM (70 μL, 0.5 mmol) in 10.0 mL volume. The flow rate was 26 μL/min. After steady state, a sample solution was collected into a graduated cylinder for 116 min (3.0 mL, 0.3 mmol). Then 0.5 mL brine was added, the mixture was extracted with ethyl acetate. The combined organic layer were dried with sodium sulfate, filtered and concentrated in vacuo then purified by column chromatography (PE) to get the product **3l** (37.5 mg, 46%) as a white solide. Mp: 71 - 73 °C Spectroscopical data in accordance with the literature[1] ^{1}H NMR (400 MHz, $CDCl_3$): δ ppm 7.91 (d, *J* = 7.6 Hz, 2H), 7.62 (d, *J* = 7.6 Hz, 2H), 1.35 (s, 12H). ^{13}C NMR (100 MHz, $CDCl_3$): δ ppm 135.0, 132.9 (q, *J* = 32.0 Hz), 125.5 (q, *J* = 271.0 Hz), 124.3 (q, *J* = 7.0 Hz), 84.2, 24.8. Carbon bearing boron not observed. ^{19}F NMR (376.5 MHz, $CDCl_3$) δ - 63.0. IR (neat, cm^{-1}): 2918, 2849, 1733, 1362, 1322, 1260, 1092, 1019.

2-(3-fluorophenyl)-4,4,5,5-tetramethyl-1,3,2-dioxaborolane(3m).

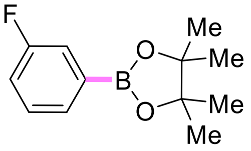

Following the general continuous metal-free borylation procedure using the set-up in **Figure S2**, syringe was loaded with a solution of 1-fluoro-3-iodobenzene (222.0 mg, 1.0 mmol), B_2pin_2 (381.0 mg, 1.5 mmol), and TMDAM (70 μL, 0.5 mmol) in 10.0 mL volume. The flow rate was 52 μL/min. After steady state, a sample solution was collected into a graduated cylinder for 58 min (3.0 mL, 0.3 mmol). Then 0.5 mL brine was added, the mixture was extracted with ethyl acetate. The combined organic layer were dried with sodium sulfate, filtered and concentrated in vacuo then purified by column chromatography (PE) to get the product **3m** (44.0 mg, 66%) as a pale yellow liquid. Spectroscopical data in accordance with the literature. [3] ^{1}H NMR (400 MHz,

$CDCl_3$): δ ppm 7.58 (d, *J* = 7.6 Hz, 1H), 7.49 (m, 1H), 7.36 (m, 1H), 7.16 (m, 1H), 1.35 (s, 12H). ^{13}C NMR (100 MHz, $CDCl_3$): δ ppm 163.7 (d, *J* = 245.0 Hz), 130.3 (d, *J* = 3.0 Hz), 129.5 (d, *J* = 7.0 Hz), 121.0 (d, *J* = 19.0 Hz), 118.3 (d, *J* =21.0 Hz), 84.1, 24.8. Carbon bearing boron not observed. ^{19}F NMR (376.5 MHz, acetone-d_6) δ - 114.2. IR (neat, cm^{-1}): 2979, 2925, 2854, 1580, 1434, 1356, 1207, 1145.

2-(4-fluorophenyl)-4,4,5,5-tetramethyl-1,3,2-dioxaborolane (3n).

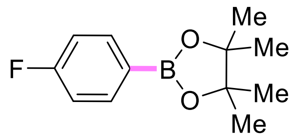

Following the general continuous metal-free borylation procedure using the set-up in **Figure S2,** syringe was loaded with a solution of 1-fluoro-4-iodobenzene (222.0 mg, 1.0 mmol), B_2pin_2 (381.0 mg, 1.5 mmol), and TMDAM (70 μL, 0.5 mmol) in 10.0 mL volume. The flow rate was 52 μL/min. After steady state, a sample solution was collected into a graduated cylinder for 58 min (3.0 mL, 0.3 mmol). Then 0.5 mL brine was added, the mixture was extracted with ethyl acetate. The combined organic layer were dried with sodium sulfate, filtered and concentrated in vacuo then purified by column chromatography (PE) to get the product **3n** (48.6 mg, 73%) as a pale yellow liquid. Spectroscopical data in accordance with the literature. [1] ^{1}H NMR (400 MHz, $CDCl_3$): δ ppm 7.81 (t, *J* = 7.4 Hz, 2H), 7.07 (t, *J* = 9.0 Hz, 2H), 1.34 (s, 12H). ^{13}C NMR (100 MHz, $CDCl_3$): δ ppm 166.3 (d, *J* = 249.0 Hz), 137.0 (d, *J* = 8.0 Hz), 114.9 (d, *J* = 20.0), 83.9, 24.8. Carbon bearing boron not observed. ^{19}F NMR (376.5 MHz, acetone-d_6) δ -108.4. IR (neat, cm^{-1}): 2956, 2923, 2852, 1716, 1361, 1269, 1144, 1108.

4,4,5,5-tetramethyl-2-(o-tolyl)-1,3,2-dioxaborolane (3o).

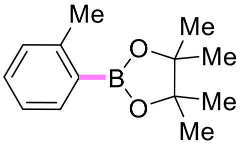

Following the general continuous metal-free borylation procedure using the set-up in **Figure S2**, syringe was loaded with a solution of 1-iodo-2-methylbenzene (218.0 mg, 1.0 mmol), B_2pin_2 (381.0 mg, 1.5 mmol), and TMDAM (70 μL, 0.5 mmol) in 10.0 mL volume. The flow rate was 52 μL/min. After steady state, a sample solution was collected into a graduated cylinder for 58 min (3.0 mL, 0.3 mmol). Then 0.5 mL brine was added, the mixture was extracted with ethyl acetate. The combined organic layer were dried with sodium sulfate, filtered and concentrated in vacuo then purified by column chromatography (PE) to get the product **3o** (45.1 mg, 69%) as a pale yellow liquid. Spectroscopical data in accordance with the literature. [2] ^{1}H NMR (400 MHz, $CDCl_3$): δ ppm 7.80 (d, *J* = 6.4 Hz, 1H), 7.36 (m, 1H), 7.21 (m, 2H), 2.57 (s, 3H), 1.37 (s, 12H). ^{13}C NMR (100 MHz, $CDCl_3$): δ ppm 144.8, 130.7, 129.7, 124.5, 83.3, 24.8, 22.2. Carbon bearing boron not observed. IR (neat, cm^{-1}): 2978, 2927, 1717, 1601, 1437, 1346, 1272, 1072.

2-(4,4,5,5-tetramethyl-1,3,2-dioxaborolan-2-yl)aniline (3p).

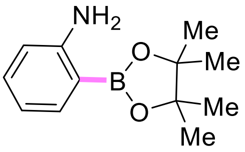

Following the general continuous metal-free borylation procedure using the set-up in **Figure S2**, syringe was loaded with a solution of 2-iodoaniline (219.0 mg, 1.0 mmol), B_2pin_2 (381.0 mg, 1.5 mmol), and TMDAM (70 μL, 0.5 mmol) in 10.0 mL volume. The flow rate was 52 μL/min. After steady state, a sample solution was collected into a graduated cylinder for 58 min (3.0 mL, 0.3 mmol). Then 0.5 mL brine was added, the mixture was extracted with ethyl acetate. The combined organic layer were dried with sodium sulfate, filtered and concentrated in vacuo then purified by column chromatography (PE/EA=10/1) to get the product **3p** (39.4 mg, 60%) as a white solide, Mp 67 - 69 ^{o}C. Spectroscopical data in accordance with the literature.[6] ^{1}H NMR (400 MHz, $CDCl_3$): δ ppm 7.61 (d, *J* = 8.8 Hz, 1H), 7.21 (t, *J* = 8.4 Hz, 1H), 6.67(t, *J* = 7.2 Hz, 1H), 6.60 (d, *J* = 8.4 Hz, 1H), 4.73 (s, 2H), 1.34 (s, 12H). ^{13}C NMR (100 MHz, $CDCl_3$): δ ppm 153.6, 136.8, 132.7, 116.8, 114.7, 83.5, 24.8. Carbon bearing boron not observed. IR (neat, cm-1): 3446, 2980, 1618, 1358, 1142, 1103.

1,4-bis(4,4,5,5-tetramethyl-1,3,2-dioxaborolan-2-yl)benzene (3q)

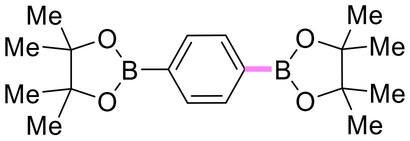

Following the general procedure for batch metal-free borylation using the set-up in **Figure S1**, a solution of the p-B(pin) iodobenzene (66.0 mg, 0.2 mmol), B_2pin_2 (101.6 mg, 0.4 mmol), TMDAM (14 μL, 0.5 eq), acetone (0.2 mL) and the additive reagents in MeCN/H_2O (4/1 v/v, 2.0 mL) was added into a quartz test tube containing a magnetic stirring bar and the mixture was purged with argon for 10 min. The tube was then capped with a septum. The reaction mixture was irradiated using a 300 W high-pressure mercury lamp through a water-cooled quartz immersion well for 4h. Then 0.5 mL brine was added, the mixture was extracted with ethyl acetate. The combined organic layer were dried with sodium sulfate, filtered and concentrated in vacuo then purified by column chromatography (EA/PE 1:100) to get the product **3q** (40.3 mg, 61%) as a white solid. Mp: 236 - 238 ^{o}C. Spectroscopical data in accordance with the literature. [3] ^{1}H NMR (400 MHz, $CDCl_3$): δ ppm 7.80 (s, 4H), 1.35 (s, 12H). ^{13}C NMR (100 MHz, $CDCl_3$): δ ppm 134.0, 83.8, 24.8. Carbon bearing boron not observed. IR (neat, cm^{-1}): 2976, 2926, 1739, 1522, 1355, 1257, 1142, 1101.

2-(4-(allyloxy)phenyl)-4,4,5,5-tetramethyl-1,3,2-dioxaborolane (3r)

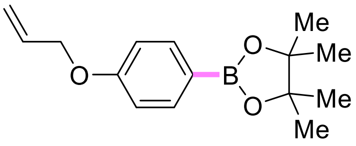

Following the general continuous metal-free borylation procedure using the set-up in **Figure S2**, syringe was loaded with a solution of 1-(allyloxy)-4-iodobenzene (260.1 mg, 1.0 mmol), B_2pin_2

(381.0 mg, 1.5 mmol), and TMDAM (70 μL, 0.5 mmol) in 10.0 mL volume. The flow rate was 52 μL/min. After steady state, a sample solution was collected into a graduated cylinder for 58 min (3.0 mL, 0.3 mmol). Then 0.5 mL brine was added, the mixture was extracted with ethyl acetate. The combined organic layer were dried with sodium sulfate, filtered and concentrated in vacuo then purified by column chromatography (PE/EA=100/1) to get the product **3r** (49.9 mg, 64%) as a pale yellow liquid. Spectroscopical data in accordance with the literature. [2] ^{1}H NMR (400 MHz, $CDCl_3$): δ ppm 7.76 (d, *J* = 8.4 Hz, 2H), 6.92 (d, *J* = 8.4 Hz, 2H), 6.09 (m, 1H), 5.43 (d, *J* = 17.2 Hz, 1H), 5.30 (d, *J* = 11.6 Hz, 1H), 4.57 (d, *J* = 5.2 Hz, 2H), 1.33 (s, 12H). ^{13}C NMR (100 MHz, $CDCl_3$): δ ppm 161.1, 136.5, 132.9, 117.4, 113.8, 83.5, 68.4, 24.8. Carbon bearing boron not observed. IR (neat, cm^{-1}): 2978, 2926, 2855, 1739, 1522, 1355, 1142, 1101.

5-(4,4,5,5-tetramethyl-1,3,2-dioxaborolan-2-yl)pyridin-2-amine (3s)

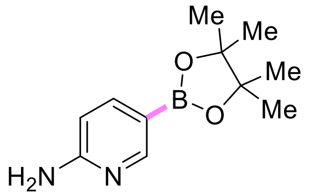

[1] Following the general procedure for batch metal-free borylation using the set-up in **Figure S1**, a solution of the 5-iodopyridin-2-amine (44.0 mg, 0.2 mmol), B_2pin_2 (101.6 mg, 0.4 mmol), TMDAM (14 μL, 0.5 eq), acetone (0.2 mL) and the additive reagents in $MeCN/H_2O$ (4/1 v/v, 2.0 mL) was added into a quartz test tube containing a magnetic stirring bar and the mixture was purged with argon for 10 min. The tube was then capped with a septum. The reaction mixture was irradiated using a 300 W high-pressure mercury lamp through a water-cooled quartz immersion well for 4h. Then 0.5 mL brine was added, the mixture was extracted with ethyl acetate. The H NMR yield determined by ^{1}H NMR with 1,3,5-trimethoxybenzene as an internal standard (**3s**, 40%).

[2] Following the general continuous metal-free borylation procedure using the set-up in **Figure S2**, syringe was loaded with a solution of 5-iodopyridin-2-amine (220.0 mg, 1.0 mmol), B_2pin_2 (381.0 mg, 1.5 mmol), and TMDAM (70 μL, 0.5 mmol) in 10.0 mL volume. The flow rate was 52 μL/min. After steady state, a sample solution was collected into a graduated cylinder for 58 min (3.0 mL, 0.3 mmol). Then 0.5 mL brine was added, the mixture was extracted with ethyl acetate. The combined organic layer were dried with sodium sulfate, filtered and concentrated in vacuo. The H NMR yield determined by ^{1}H NMR with 1,3,5-trimethoxybenzene as an internal standard (**3s**, 50%).

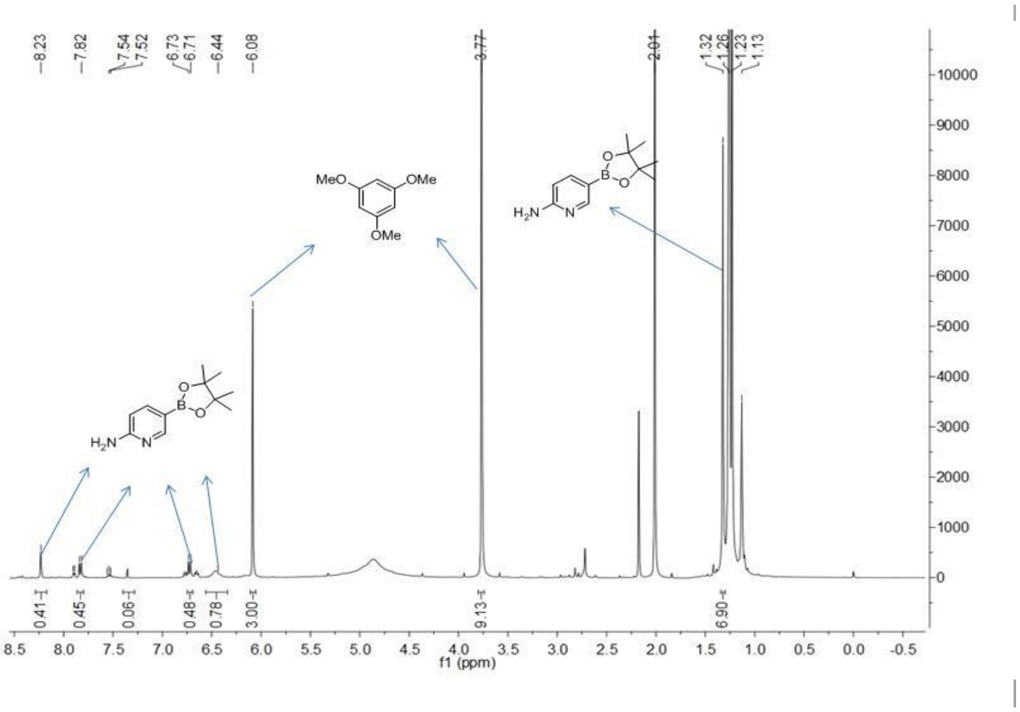

(The crude ^{1}H NMR spectrum with 1,3,5-trimethoxybenzene as an internal standard)

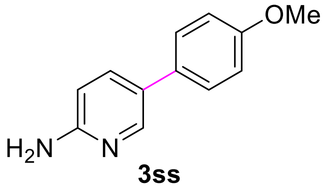

3ss

[3] Subsequent Pd-catalyzed SMC reactions：

(In order to prove the compound **3s** was obtained**)**

Following the general procedure for batch metal-free borylation using the set-up in **Figure S1**, a solution of the 5-iodopyridin-2-amine (44.0 mg, 0.2 mmol), B_2pin_2 (101.6 mg, 0.4 mmol), TMDAM (14 μL, 0.5 eq), acetone (0.2 mL) and the additive reagents in MeCN/H_2O (4/1 v/v, 2.0 mL) was added into a quartz test tube containing a magnetic stirring bar and the mixture was purged with argon for 10 min. The tube was then capped with a septum. The reaction mixture was irradiated using a 300 W high-pressure mercury lamp through a water-cooled quartz immersion well for 4 h. And then the solvent was removed under reduced pressure. $Pd(PPh_3)_4$ (5 mol%) and K_2CO_3 (0.4 mmol, 2eq) were loaded into a Schlenk tube equipped with a Teflon-coated magnetic bar. The tube was evacuated and back-filled with argon for three times. Pre-complexation was accomplished by adding 2.0 mL DMF, 4-iodoanisole (0.4 mmol, 2eq) into the above crude arylboronate product into the Schlenk tube and the solution was stirred and heated at 90 ^{o}C overnight. After completion of the reaction, the reaction tube was allowed to cool to room temperature, and the solution was filtered through a short column of silica gel and washed with EtOAc (~20 mL). The filtrate was concentrated and purified by flash column chromatography on silica gel to afford the final product **3ss** (18.0 mg, 45%), Spectroscopical data in accordance with the literature. [13] ^{1}H NMR (400 MHz, CDCl3): δ ppm 8.25 (s, 1H), 7.63 (d, J = 8.4 Hz, 1H), 7.42 (d, J = 8.4 Hz, 2H), 6.97 (d, J = 8.4 Hz, 2H), 6.58 (d, J = 8.4 Hz, 1H), 4.34 (s, 2H), 3.83 (s, 3H).

^{13}C NMR (100 MHz, CDCl3): δ ppm 158.8, 157.0, 145.3, 136.4, 130.7, 127.3, 127.1, 114.3, 108.6, 55.3.

methyl 4-(4,4,5,5-tetramethyl-1,3,2-dioxaborolan-2-yl)benzoate (3t)

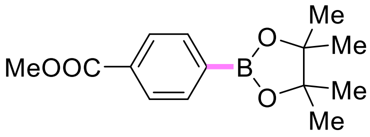

Following the general continuous metal-free borylation procedure using the set-up in **Figure S2**, syringe was loaded with a solution of methyl 4-bromobenzoate (215.0 mg, 1.0 mmol), B_2pin_2 (381.0 mg, 1.5 mmol), and TMDAM (70 µL, 0.5 mmol) in 10.0 mL volume. The flow rate was 52 µL/min. After steady state, a sample solution was collected into a graduated cylinder for 58 min (3.0 mL, 0.3 mmol). Then 0.5 mL brine was added, the mixture was extracted with ethyl acetate. The combined organic layer were dried with sodium sulfate, filtered and concentrated in vacuo then purified by column chromatography (PE/EA=20/1) to get the product **3t** (73.1 mg, 93%) as a white solide. Mp: 75 - 77 ^{o}C. Spectroscopical data in accordance with the literature. [7]
^{1}H NMR (400 MHz, $CDCl_3$): δ ppm 8.03 (d, J = 8.0 Hz, 1H), 7.87 (d, J = 8.4 Hz, 1H), 3.92 (s, 3H), 1.35 (s, 12H). ^{13}C NMR (100 MHz, $CDCl_3$): δ ppm 167.1, 134.6, 131.9, 128.5, 83.9, 52.1, 24.8. Carbon bearing boron not observed. IR (neat, cm^{-1}): 2981, 2926, 2854, 1732, 1362, 1271, 1143, 1020.

2-(4-chlorophenyl)-4,4,5,5-tetramethyl-1,3,2-dioxaborolane (3u)

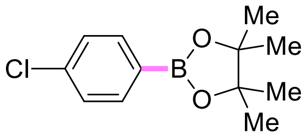

[1] Following the general procedure for batch metal-free borylation using the set-up in **Figure S1**, a solution of the 1-bromo-4-chlorobenzene (38.3 mg, 0.2 mmol), B_2pin_2 (101.6 mg, 0.4 mmol), TMDAM (14 µL, 0.5 eq), acetone (0.2 mL) and the additive reagents in $MeCN/H_2O$ (4/1 v/v, 2.0 mL) was added into a quartz test tube containing a magnetic stirring bar and the mixture was purged with argon for 10 min. The tube was then capped with a septum. The reaction mixture was irradiated using a 300 W high-pressure mercury lamp through a water-cooled quartz immersion well for 4h. Then 0.5 mL brine was added, the mixture was extracted with ethyl acetate. The combined organic layer were dried with sodium sulfate, filtered and concentrated in vacuo then purified by column chromatography (EA/PE = 1:100) to get the product **3u** (26.7 mg, 56%) as a pale yellow liquid.
[2] Following the general continuous metal-free borylation procedure using the set-up in **Figure S2**, syringe was loaded with a solution of 1-bromo-4-chlorobenzene (191.0 mg, 1.0 mmol), B_2pin_2 (381.0 mg, 1.5 mmol), and TMDAM (70 µL, 0.5 mmol) in 10.0 mL volume. The flow rate was 26 µL/min. After steady state, a sample solution was collected into a graduated cylinder for 116 min (3.0 mL, 0.3 mmol). Then 0.5 mL brine was added, the mixture was extracted with ethyl acetate. The combined organic layer were dried with sodium sulfate, filtered and concentrated in vacuo then purified by column chromatography (EA/PE = 1:100) to get the product **3u** (41.5 mg, 58%) as a pale yellow liquid, Spectroscopical data in accordance with the literature.[3] ^{1}H NMR (400

MHz, $CDCl_3$): δ ppm 7.74 (d, *J* = 7.6 Hz, 2H), 7.35 (d, *J* = 8.0 Hz, 2H), 1.34 (s, 12H). ^{13}C NMR (100 MHz, $CDCl_3$): δ ppm 137.5, 136.1, 127.9, 83.9, 24.8. Carbon bearing boron not observed. IR (neat, cm^{-1}): 2979, 2929, 1716, 1596, 1489, 1393, 1359, 1143, 1092.

4,4,5,5-tetramethyl-2-(p-tolyl)-1,3,2-dioxaborolane (3v)

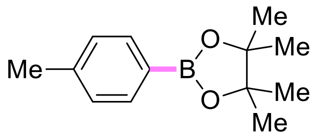

[1] Following the general procedure for batch metal-free borylation using the set-up in **Figure S1**, a solution of the 1-bromo-4-methylbenzene (34.2 mg, 0.2 mmol), B_2pin_2 (101.6 mg, 0.4 mmol), TMDAM (14 μL, 0.5 eq), acetone (0.2 mL) and the additive reagents in MeCN/H_2O (4/1 v/v, 2.0 mL) was added into a quartz test tube containing a magnetic stirring bar and the mixture was purged with argon for 10 min. The tube was then capped with a septum. The reaction mixture was irradiated using a 300 W high-pressure mercury lamp through a water-cooled quartz immersion well for 4h. Then 0.5 mL brine was added, the mixture was extracted with ethyl acetate. The combined organic layer were dried with sodium sulfate, filtered and concentrated in vacuo then purified by column chromatography (PE) to get the product **3v** (26.7 mg, 31%) as a pale yellow liquid.

[2] Following the general continuous metal-free borylation procedure using the set-up in **Figure S2**, syringe was loaded with a solution of 1-bromo-4-methylbenzene (171.0 mg, 1.0 mmol), B_2pin_2 (381.0 mg, 1.5 mmol), and TMDAM (70 μL, 0.5 mmol) in 10.0 mL volume. The flow rate was 26 μL/min. After steady state, a sample solution was collected into a graduated cylinder for 116 min (3.0 mL, 0.3 mmol). Then 0.5 mL brine was added, the mixture was extracted with ethyl acetate. The combined organic layer were dried with sodium sulfate, filtered and concentrated in vacuo then purified by column chromatography (PE) to get the product **3v** (41.5 mg, 50%) as a pale yellow liquid, Spectroscopical data in accordance with the literature.[3] ^{1}H NMR (400 MHz, $CDCl_3$): δ ppm 7.72 (d, *J* = 8.0 Hz, 2H), 7.20 (d, *J* = 7.6 Hz, 2H), 2.37 (s, 3H), 1.34 (s, 12H). ^{13}C NMR (100 MHz, $CDCl_3$): δ ppm 137.5, 136.1, 127.9, 83.9, 24.8. Carbon bearing boron not observed. IR (neat, cm^{-1}): 2956, 2853, 1734, 1457, 1374, 1361, 1244, 1047..

2-(benzo[d][1,3]dioxol-5-yl)-4,4,5,5-tetramethyl-1,3,2-dioxaborolane (3w)

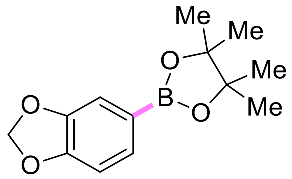

[1] Following the general procedure for batch metal-free borylation using the set-up in **Figure S1**, a solution of the 5-bromobenzo[d][1,3]dioxole (40.2 mg, 0.2 mmol), B_2pin_2 (101.6 mg, 0.4 mmol), TMDAM (14 μL, 0.5 eq), acetone (0.2 mL) and the additive reagents in MeCN/H_2O (4/1 v/v, 2.0 mL) was added into a quartz test tube containing a magnetic stirring bar and the mixture was purged with argon for 10 min. The tube was then capped with a septum. The reaction mixture was irradiated using a 300 W high-pressure mercury lamp through a water-cooled quartz immersion well for 4h. Then 0.5 mL brine was added, the mixture was extracted with ethyl acetate. The combined organic layer were dried with sodium sulfate, filtered and concentrated in vacuo then

purified by column chromatography (EA/PE = 1:100) to get the product **3w** (26.7 mg, 15%) as a pale yellow liquid.

[2] Following the general continuous metal-free borylation procedure using the set-up in **Figure S2**, syringe was loaded with a solution of 5-bromobenzo[d][1,3]dioxole (201.0 mg, 1.0 mmol), B_2pin_2 (381.0 mg, 1.5 mmol), and TMDAM (70 μL, 0.5 mmol) in 10.0 mL volume. The flow rate was 26 μL/min. After steady state, a sample solution was collected into a graduated cylinder for 116 min (3.0 mL, 0.3 mmol). Then 0.5 mL brine was added, the mixture was extracted with ethyl acetate. The combined organic layer were dried with sodium sulfate, filtered and concentrated in vacuo then purified by column chromatography (EA/PE = 1:100) to get the product **3w** (41.7 mg, 56%) as a pale yellow liquid, Spectroscopical data in accordance with the literature.[3] ^{1}H NMR (400 MHz, CDCl3): δ ppm 7.36 (d, J = 7.6 Hz, 1H), 7.24 (s, 1H), 6.83 (d, J = 8.0 Hz, 1H), 5.95 (s, 2H), 1.33 (s, 12H). ^{13}C NMR (100 MHz, CDCl3): δ ppm 150.1, 147.2, 129.7, 113.9, 108.2, 100.7, 83.7, 24.8.Carbon bearing boron not observed. IR (neat, cm-1): 2979, 1605, 1356, 1144, 1106, 1039.

N,N-dimethyl-4-(4,4,5,5-tetramethyl-1,3,2-dioxaborolan-2-yl)aniline (3x)

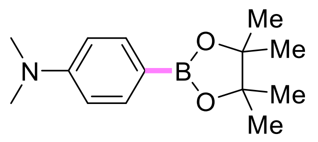

Following the general continuous metal-free borylation procedure using the set-up in **Figure S2**, syringe was loaded with a solution of 4-bromo-N,N-dimethylaniline (200.0 mg, 1.0 mmol), B_2pin_2 (381.0 mg, 1.5 mmol), and TMDAM (70 μL, 0.5 mmol) in 10.0 mL volume. The flow rate was 26 μL/min. After steady state, a sample solution was collected into a graduated cylinder for 116 min (3.0 mL, 0.3 mmol). Then 0.5 mL brine was added, the mixture was extracted with ethyl acetate. The combined organic layer were dried with sodium sulfate, filtered and concentrated in vacuo then purified by column chromatography (EA/PE = 1:100) to get the product **3x** (27.4 mg, 37%) as a white solid. Spectroscopical data in accordance with the literature.[2] ^{1}H NMR (400 MHz, $CDCl_3$): δ ppm 7.69 (d, *J* = 8.8 Hz, 2H), 6.69 (d, *J* = 8.8 Hz, 2H), 2.98 (s, 6H), 1.32 (s, 12H). ^{13}C NMR (100 MHz, $CDCl_3$): δ ppm 152.5, 136.1, 111.2, 83.1, 40.1, 24.8. Carbon bearing boron not observed. IR (neat, cm^{-1}):2978, 1605, 1361, 1247, 1143, 1030.

4-(5,5-dimethyl-1,3,2-dioxaborinan-2-yl)phenol (3y)

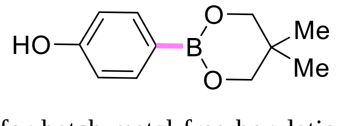

Following the general procedure for batch metal-free borylation using the set-up in Figure S3, a solution of the 4-iodophenol (44.0 mg, 0.2 mmol), B_2neop_2 (90.3 mg, 0.4 mmol), TMDAM (14 μL, 0.5 eq), acetone (0.2 mL) and the additive reagents in $MeCN/H_2O$ (4/1 v/v, 2.0 mL) was added into a quartz test tube containing a magnetic stirring bar and the mixture was purged with argon for 10 min. The tube was then capped with a septum. The reaction mixture was irradiated using a 300 W high-pressure mercury lamp through a water-cooled quartz immersion well for 4h. Then 0.5 mL brine was added, the mixture was extracted with ethyl acetate. The combined organic layer were dried with sodium sulfate, filtered and concentrated in vacuo then purified by column

chromatography (EA/PE 1:30) to get the product **3u** (30.9 mg, 75%) as a white solide, Mp 81 - 83 ^{o}C. Spectroscopical data in accordance with the literature. [8] ^{1}H NMR (400 MHz, $CDCl_3$): δ ppm 7.71 (d, *J* = 7.6 Hz, 2H), 6.81 (d, *J* = 7.2 Hz, 2H), 4.95 (s, 1H), 3.75 (s, 4H), 1.01 (s, 6H). ^{13}C NMR (100 MHz, $CDCl_3$): δ ppm 157.8, 135.8, 114.6, 72.2, 31.9, 21.9. Carbon bearing boron not observed. IR (neat, cm-1): 3392, 2962, 1606, 1344, 1171, 1134.

4-(5,5-dimethyl-1,3,2-dioxaborinan-2-yl)benzonitrile (3z)

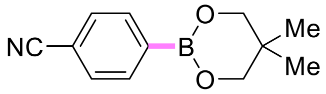

Following the general procedure for batch metal-free borylation using the set-up in Figure S3, a solution of the 4-iodobenzonitrile (45.8 mg, 0.2 mmol), B_2neop_2 (90.3 mg, 0.4 mmol), TMDAM (14 μL, 0.5 eq), acetone (0.2 mL) and the additive reagents in MeCN/H_2O (4/1 v/v, 2.0 mL) was added into a quartz test tube containing a magnetic stirring bar and the mixture was purged with argon for 10 min. The tube was then capped with a septum. The reaction mixture was irradiated using a 300 W high-pressure mercury lamp through a water-cooled quartz immersion well for 4h. Then 0.5 mL brine was added, the mixture was extracted with ethyl acetate. The combined organic layer were dried with sodium sulfate, filtered and concentrated in vacuo then purified by column chromatography (EA/PE 1:50) to get the product **3v** (34.0 mg, 79%) as a white solide. Mp: 113 - 115 ^{o}C. Spectroscopical data in accordance with the literature. [9] ^{1}H NMR (400 MHz, $CDCl_3$): δ ppm 7.88 (d, *J* = 7.6 Hz, 2H), 7.62 (d, *J* = 7.6 Hz, 2H), 3.78 (s, 4H), 1.02 (s, 6H). ^{13}C NMR (100 MHz, $CDCl_3$): δ ppm 134.2, 131.0, 119.1, 114.0, 72.4, 31.9, 21.7. Carbon bearing boron not observed. IR (neat, cm^{-1}): 2960, 2935, 2227, 1734, 1603, 1476, 1206, 1151.

2-(4-methoxyphenyl)-2,3-dihydro-1H-naphtho[1,8-de][1,3,2]diazaborinine (3aa)

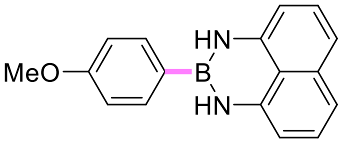

Following the general procedure for batch metal-free borylation using the set-up in Figure S3, a solution of the 1-iodo-4-methoxybenzene (46.8 mg, 0.2 mmol), Bpin-Bdan (117.6 mg, 0.4 mmol), TMDAM (14 μL, 0.5 eq), acetone (0.2 mL) and the additive reagents in MeCN/H_2O (4/1 v/v, 2.0 mL) was added into a quartz test tube containing a magnetic stirring bar and the mixture was purged with argon for 10 min. The tube was then capped with a septum. The reaction mixture was irradiated using a 300 W high-pressure mercury lamp through a water-cooled quartz immersion well for 4h. Then 0.5 mL brine was added, the mixture was extracted with ethyl acetate. The combined organic layer were dried with sodium sulfate, filtered and concentrated in vacuo then purified by column chromatography (EA/PE 1:30) to get the product **3w** (21.9 mg, 40%) as a white solide. Mp163 - 165 ^{o}C. Spectroscopical data in accordance with the literature. [10] ^{1}H NMR (400 MHz, $CDCl_3$) δ 7.60 (d, *J* = 8.8 Hz, 2H), 7.16 (t, *J* = 8.0 Hz, 2H), 7.06 (d, *J* = 8.0 Hz, 2H), 6.99 (d, *J* = 8.4 Hz, 2H), 6.42 (d, *J* = 7.2 Hz, 2H), 5.99 (s, 2H), 3.86 (s, 3H). ^{13}C NMR (100 MHz, $CDCl_3$) δ 161.5, 141.3, 136.4, 133.1, 127.7, 119.8, 117.8, 114.0, 106.0, 55.3. ^{11}B NMR (128 MHz, $CDCl_3$) δ 29.1. HRMS (APCI) m/z calcd for $C_{17}H_{14}BN_2O$ (M-): 273.1205, found: 273.1203. IR

(cm^{-1}): 3407, 1594, 1495, 1407, 1224, 1181, 1029.

2-(4-methoxyphenyl)-2,3-dihydro-1H-naphtho[1,8-de][1,3,2]diazaborinine (3ab)

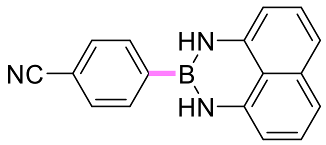

Following the general procedure for batch metal-free borylation using the set-up in Figure S3, a solution of the 4-iodobenzonitrile (45.8 mg, 0.2 mmol), Bpin-Bdan (117.6 mg, 0.4 mmol), TMDAM (14 μL, 0.5 eq), acetone (0.2 mL) and the additive reagents in MeCN/H_2O (4/1 v/v, 2.0 mL) was added into a quartz test tube containing a magnetic stirring bar and the mixture was purged with argon for 10 min. The tube was then capped with a septum. The reaction mixture was irradiated using a 300 W high-pressure mercury lamp through a water-cooled quartz immersion well for 4h. Then 0.5 mL brine was added, the mixture was extracted with ethyl acetate. The combined organic layer were dried with sodium sulfate, filtered and concentrated in vacuo then purified by column chromatography (EA/PE 1:14) to get the product **3x** (24.2 mg, 45%) as a white solide. Mp: 220 - 225 ^oC. Spectroscopical data in accordance with the literature. [10] ^1H NMR (400 MHz, $CDCl_3$) δ 7.72 (dd, *J* = 8.0, 3.2 Hz, 4H), 7.17 (t, *J* = 7.8, 2H), 7.09 (d, *J* = 8.0 Hz, 2H), 6.44 (d, *J* = 7.2 Hz, 2H), 5.99 (br, 2H). ^{13}C NMR (100 MHz, $CDCl_3$) δ 140.5, 136.4, 132.1, 131.8, 127.7, 120.1, 118.8, 118.6, 113.8, 106.5. HRMS (ESI) m/z calcd for $C_{17}H_{12}BN_3Na$ (M+): 292.1022, found: 292.1014. ^{11}B NMR (128 MHz, $CDCl_3$) δ 28.8. IR (cm^{-1}): 3383, 2924, 2231, 1595, 1527, 1408, 1397, 1084.

C）Typical procedure for the synthesis of trifluoroborates

An oven-dried screwcapping volumetric flask (10.0 mL) was charged with aryl halide **3** (1.0 mmol, if solid, 1 eq) and $B_2(OH)_4$ (1.5 mmol, 1.5 eq) then capped with a septum. The vessel was evacuated and back-filled with argon (this process was carried out a total of 3 times). **3** (1.0 mmol, if liquid, 1 eq), H_2O (2.0 mL) were added by syringes and MeOH was added to dissolve the solids and filled up to volume. Before reaction all the solutions were prepared under argon atmosphere and the reactors tubing were purged with MeOH three times at least. After that, all reactors and connecting tubings were filled with MeOH. A 10 mL disposable syringe was used to pump the reaction solution through the continuous-flow reactor (the set-up in **Figure S2**) at controlled flow rates. A cooling liquid circulating pump is used to maintain the temperature around the tubing is -5 ^oC. After reaching steady state, a sample of the reaction mixture was collected which contained theoretical yield of 0.8 mmol based on flow rate and collecting time. The crude reaction was cooled to 0 ^oC and 6.5 equivalents of a 4.5 M aqueous KHF_2 (2.8 mmol) solution was added. The reaction was stirred for 10 min at 0 ^oC before removing the bath and allowing the mixture to stir at rt until the conversion to the corresponding trifluoroborate was achieved as determined by 11B NMR. After conversion, the mixture was concentrated and further dried under high vacuum Soxhlet extraction with acetone (50 mL) for 12 hours. The collected solvent was concentrated and then dissolved in a minimal volume of acetone (~ 2 mL). The addition of Et_2O (~30 mL) led to the precipitation of the desired product. At last, the product was collected by filtration and washed with Et_2O (2*10 mL).

Potassium (4-Methoxyphenyl) trifluoroborate (8a)

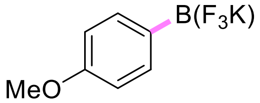

Following the general procedure **C**, syringe was loaded with a solution of 1-iodo-4-methoxybenzene (234.0 mg, 1.0 mmol), $B_2(OH)_2$ (134.4 mg, 1.5 mmol), in 10.0 mL volume (MeOH/H_2O = 4/1). The flow rate was 78 µL/min. After steady state, a sample solution was collected into a graduated cylinder for 102.6 min (8.0 mL, 0.8 mmol). The crude reaction was moved to a 25 mL round bottom flask and cooled to 0 ^{o}C, then KHF_2 was added (1.2 mL of a 4.5 M aqueous solution, 6.5 equiv). The title compound was obtained as a white solid in 93% yield (159.2 mg). Mp: > 250 ^{o}C. Spectral data were in accordance with those published. [11] ^{1}H NMR (400 MHz, DMSO-d_6) δ 7.22 (d, *J* = 7.2 Hz, 2H), 6.66 (d, *J* = 7.6 Hz, 2H), 3.66 (s, 3H). ^{13}C NMR (100 MHz, DMSO-d_6) δ 157.2, 132.2, 111.8, 54.4. ^{11}B NMR (128 MHz, DMSO-d_6) δ 2.76. ^{19}F NMR (376 MHz, DMSO-d_6) δ -138.2. IR (neat, cm^{-1}): 3016, 2955, 2837, 1918, 1605, 1281, 1178, 1032.

Potassium O-Tolyltrifluoroborate (8b)

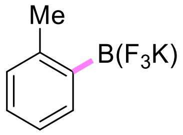

Following the general procedure **C**, syringe was loaded with a solution of 1-iodo-2-methylbenzene (218.0 mg, 1.0 mmol), $B_2(OH)_2$ (134.4 mg, 1.5 mmol), in 10.0 mL volume (MeOH/H_2O = 4/1). The flow rate was 78 µL/min. After steady state, a sample solution was collected into a graduated cylinder for 102.6 min (8.0 mL, 0.8 mmol). The crude reaction was moved to a 25 mL round bottom flask and cooled to 0 ^{o}C, then KHF_2 was added (1.2 mL of a 4.5 M aqueous solution, 6.5 equiv). The title compound was obtained as a white solid in 85% yield (134.7 mg). Mp: 227 - 229 ^{o}C. Spectral data were in accordance with those published. [11] ^{1}H NMR (400 MHz, acetone-d_6) δ 7.47 (d, *J* = 5.6 Hz, 1H), 6.89 (s, 3H), 2.38 (s, 3H). ^{13}C NMR (100 MHz, acetone-d_6) δ 141.9, 132.8, 129.2, 126.4, 124.3, 22.1. ^{11}B NMR (128 MHz, acetone-d_6) δ 3.44. ^{19}F NMR (376 MHz, acetone-d_6) δ -140.5. IR (neat, cm^{-1}): 3054, 3015, 2724, 1615, 1540, 1274, 1212, 952.

Potassium (4-Cyanophenyl) trifluoroborate (8c)

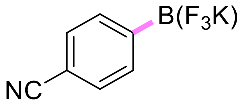

Following the general procedure **C**, syringe was loaded with a solution of 4-iodobenzonitrile (229.0 mg, 1.0 mmol), $B_2(OH)_2$ (134.4 mg, 1.5 mmol), in 10.0 mL volume (MeOH/H_2O = 4/1). The flow rate was 78 µL/min. After steady state, a sample solution was collected into a graduated cylinder for 102.6 min (8.0 mL, 0.8 mmol). The crude reaction was moved to a 25 mL round bottom flask and cooled to 0 ^{o}C, then KHF_2 was added (1.2 mL of a 4.5 M aqueous solution, 6.5 equiv). The title compound was obtained as a white solid in 75% yield (125.3 mg). Mp: > 250 ^{o}C.

Spectral data were in accordance with those published. [11] ^{1}H NMR (400 MHz, acetone-d_6) δ 7.63 (d, *J* = 7.2 Hz, 2H), 7.46 (d, *J* = 7.6 Hz, 2H). ^{13}C NMR (100 MHz, DMSO-d_6) δ 132.0, 129.9, 120.0, 107.6. ^{11}B NMR (128 MHz, DMSO-d_6) δ 2.34. ^{19}F NMR (376 MHz, DMSO-d_6) δ -140.4. IR (neat, cm^{-1}): 3078, 3051, 3032, 2231, 1393, 1211, 1190, 945.

Potassium (4-Fluorophenyl) trifluoroborate (8d)

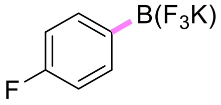

Following the general procedure **C**, syringe was loaded with a solution of 1-fluoro-4-iodobenzene (222.0 mg, 1.0 mmol), $B_2(OH)_2$ (134.4 mg, 1.5 mmol), in 10.0 mL volume ($MeOH/H_2O$ = 4/1). The flow rate was 78 μL/min. After steady state, a sample solution was collected into a graduated cylinder for 102.6 min (8.0 mL, 0.8 mmol). The crude reaction was moved to a 25 mL round bottom flask and cooled to 0 ^{o}C, then KHF_2 was added (1.2 mL of a 4.5 M aqueous solution, 6.5 equiv). The title compound was obtained as a white solid in 95% yield (153.5 mg). Mp: > 250 ^{o}C. Spectral data were in accordance with those published. [11] ^{1}H NMR (400 MHz, DMSO-d_6) δ 7.32 (t, *J* = 6.8 Hz, 2H), 6.88 (t, *J* = 8.8 Hz, 2H). ^{13}C NMR (100 MHz, DMSO-d_6) δ 162.0, 159.7, 132.8 (d, *J* = 8.0 Hz), 112.7 (d, *J* = 19.0 Hz). ^{11}B NMR (128 MHz, DMSO-d_6) δ 2.59. ^{19}F NMR (376 MHz, DMSO-d_6) δ - 118.6, -139.2. IR (neat, cm^{-1}): 3027, 1917, 1607, 1396, 1226, 1212, 969.

Potassium Pyridine-3-yl trifluoroborate (8e)

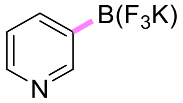

Following the general procedure **C**, syringe was loaded with a solution of 3-iodopyridine (205.0 mg, 1.0 mmol), $B_2(OH)_2$ (134.4 mg, 1.5 mmol), in 10.0 mL volume ($MeOH/H_2O$ = 4/1). The flow rate was 78 μL/min. After steady state, a sample solution was collected into a graduated cylinder for 102.6 min (8.0 mL, 0.8 mmol). The crude reaction was moved to a 25 mL round bottom flask and cooled to 0 ^{o}C, then KHF_2 was added (1.2 mL of a 4.5 M aqueous solution, 6.5 equiv). The title compound was obtained as a white solid in 89% yield (132.0 mg). Mp: 222 - 225 ^{o}C. ^{1}H NMR (400 MHz, DMSO-d_6) δ 8.46 (s, 1H), 8.25 (d, *J* = 4.0 Hz, 1H), 7.61 (d, *J* = 6.8 Hz, 1H), 7.10 (t, *J* = 5.8 Hz, 1H). ^{13}C NMR (100 MHz, DMSO-d_6) δ 152.5, 146.3, 138.6, 122.2. ^{11}B NMR (128 MHz, DMSO-d_6) δ 2.62. ^{19}F NMR (376 MHz, DMSO-d_6) δ -139.0. HRMS (ESI$^-$): Calculated for $C_5H_4BF_3N$ $(M-K)^-$: 146.0389, Found: 146.0394. IR (neat, cm^{-1}): 3039, 3023, 1590, 1404, 1224, 1190, 1049, 926.

Potassium (4-Ethoxycarbonyl)phenyl trifluoroborate (8f)

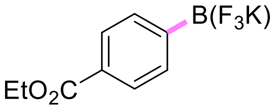

Following the general procedure **C**, syringe was loaded with a solution of ethyl 4-bromobenzoate (229.0 mg, 1.0 mmol), $B_2(OH)_2$ (134.4 mg, 1.5 mmol), in 10.0 mL volume ($MeOH/H_2O$ = 4/1). The flow rate was 78 μL/min. After steady state, a sample solution was collected into a graduated

cylinder for 102.6 min (8.0 mL, 0.8 mmol). The crude reaction was moved to a 25 mL round bottom flask and cooled to 0 ^{o}C, then KHF_2 was added (1.2 mL of a 4.5 M aqueous solution, 6.5 equiv). The title compound was obtained as a white solid in 54% yield (110.6 mg). Mp: 207 – 209 ^{o}C.

^{1}H NMR (400 MHz, DMSO-d_6) δ 7.72 (d, J = 7.2 Hz, 2H), 7.45 (d, J = 7.2 Hz, 2H). 4.29 (q, J_1 = 6.8 Hz, J_2 = 6.8 Hz, 2H), 1.32 (t, J = 6.8 Hz, 3H). ^{13}C NMR (100 MHz, DMSO-d_6) δ 166.6, 131.3, 127.1, 126.6, 59.9, 14.2. ^{11}B NMR (128 MHz, DMSO-d_6) δ 2.35. ^{19}F NMR (376 MHz, DMSO-d_6) δ -139.7. HRMS (ESI$^-$): Calculated for $C_9H_9BF_3O_2$ (M-K)$^-$: 217.0648, Found: 217.0656. IR (neat, cm^{-1}): 3039, 2983, 1716, 1592, 1367, 1267, 1123, 958.

6. References

[1] H. Kinuta, M. Tobisu, N. Chatani, *J. Am. Chem. Soc*. 2015, **137**, 1593.

[2] E. Yamamoto, K. Izumi, Y. Horita, H. Ito, *J. Am. Chem. Soc*. 2012, **134**, 19997.

[3] D. Qiu, L. Jin, Z. Zheng, H. Meng, F. Mo, X. Wang, Y. Zhang, J. Wang, *J. Org. Chem.* 2013, **78**, 1923.

[4] F. Mo, Y. Jiang, D. Qiu, Y. Zhang, J. Wang, *Angew. Chem. Int. Ed.* 2010, **49**, 1846.

[5] W. K. Chow, O. Y. Yuen, C. M. So, W. T. Wong, F. Y. Kwong, *J. Org. Chem.* 2012, **77**, 3543.

[6] T. Yamamoto, T. Morita, J. Takagi, T. Yamakawa, *Org. Lett.* 2011, **13**, 5766.

[7] Y. Nagashima, R. Takita, K. Yoshida, K. Hirano, M. Uchiyama, *J. Am. Chem. Soc*. 2013, **135**, 18730.

[8] C. Moldoveanu, D. A. Wilson, C. J. Wilson, P. Corcoran, B. M. Rosen, V, Percec, *Org. Lett.* 2009, **11**, 4974.

[9] D. A. Wilson, C. J. Wilson, C. Moldoveanu, A. M. Resmerita, P. Corcoran, L. M. Hoang, B. M. Rosen, V. Percec, *J. Am. Chem. Soc*. 2010, **132**, 1800.

[10] L. Xu and P. Li, *Chem. Commun.* 2015, **51**, 5656.

[11] G. A. Molander, S. L. J. Trice, S. D. Dreher, *J. Am. Chem. Soc*. 2010, **132**, 17701.

[12] M. G. Chini, R. D. Simone, I. Bruno, R. Riccio, F. Dehm, C. Weinigel, D. Barz, O. Werz, G. Bifulco, *European Journal of Medicinal Chemistry.* 2012, **54**, 311.

[13] T. Hoshi, T. Honma, A. Mori, M. Konishi, T. Sato, H. Hagiwara, T. Suzuki, *J. Org. Chem.* 2013, **78**, 11513.

7. Copies of NMR

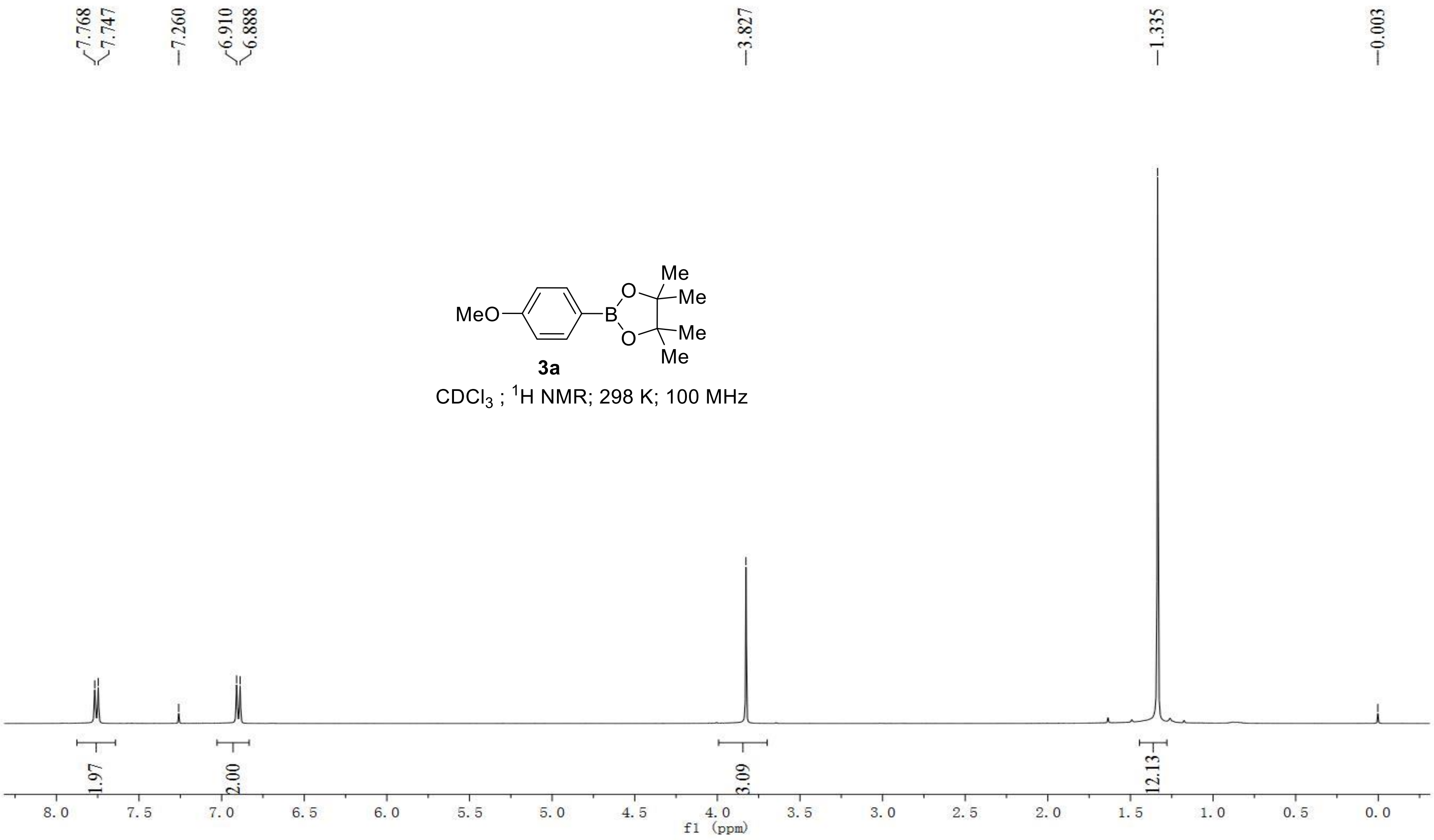
7.768
7.747
7.260
6.910
6.888
3.827
1.335
-0.003
MeO
B
O
O
Me
Me
Me
Me
3a
CDCl3 ; 1H NMR; 298 K; 100 MHz
1.97
2.00
3.09
12.13
8.0
7.5
7.0
6.5
6.0
5.5
5.0
4.5
4.0
3.5
3.0
2.5
2.0
1.5
1.0
0.5
0.0
f1 (ppm)

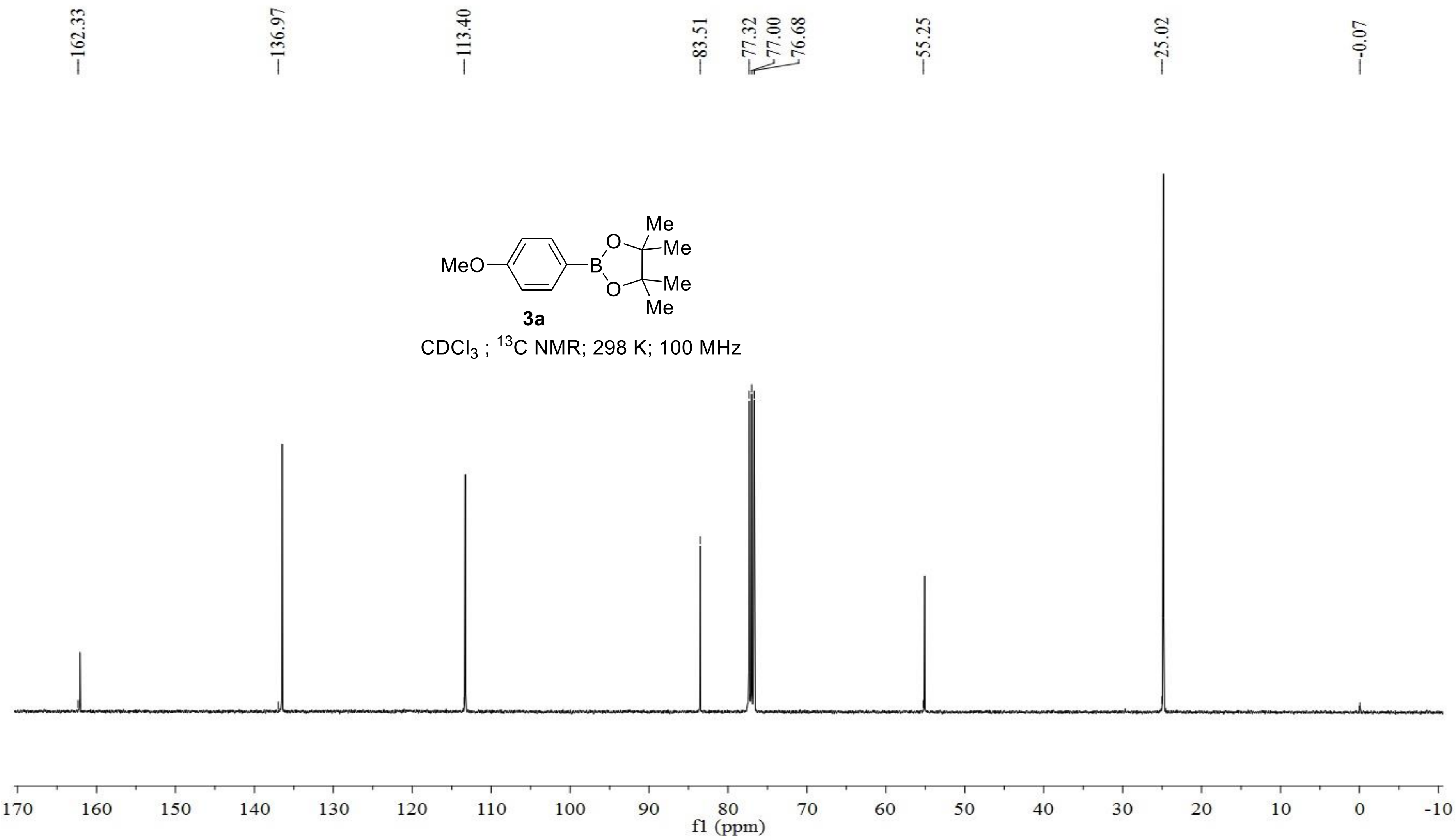

162.33
136.97
113.40
83.51
77.32
77.00
76.68
55.25
25.02
-0.07
MeO
B
O
O
Me
Me
Me
Me
3a
CDCl3 ; 13C NMR; 298 K; 100 MHz
170
160
150
140
130
120
110
100
90
80
70
60
50
40
30
20
10
0
-10
f1 (ppm)

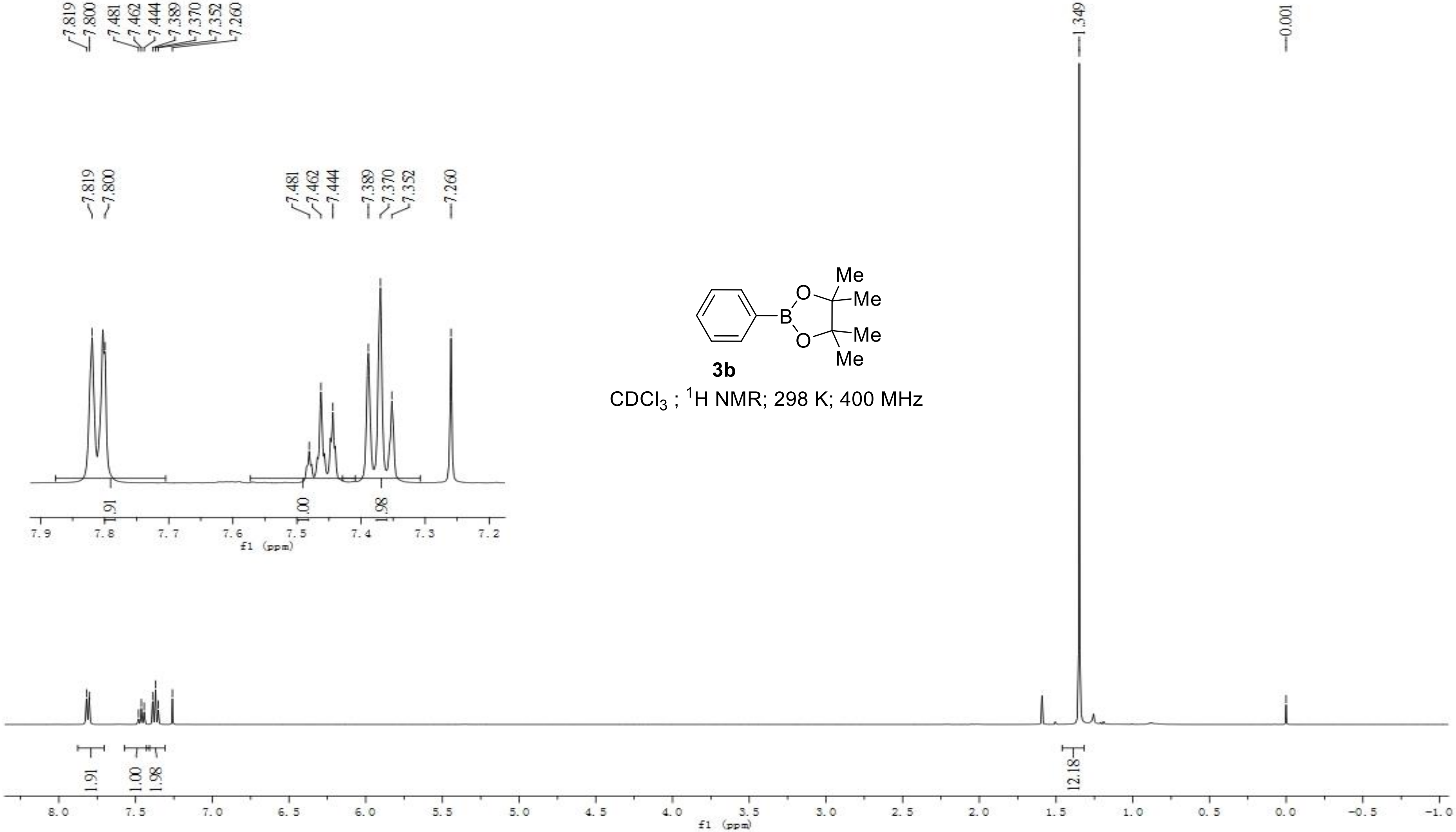
7.819
7.800
7.481
7.462
7.444
7.389
7.370
7.352
7.260
1.349
-0.001
1.91
1.00
1.98
12.18
Me
Me
Me
Me
O
O
B
3b
CDCl$_3$; ^{1}H NMR; 298 K; 400 MHz
f1 (ppm)

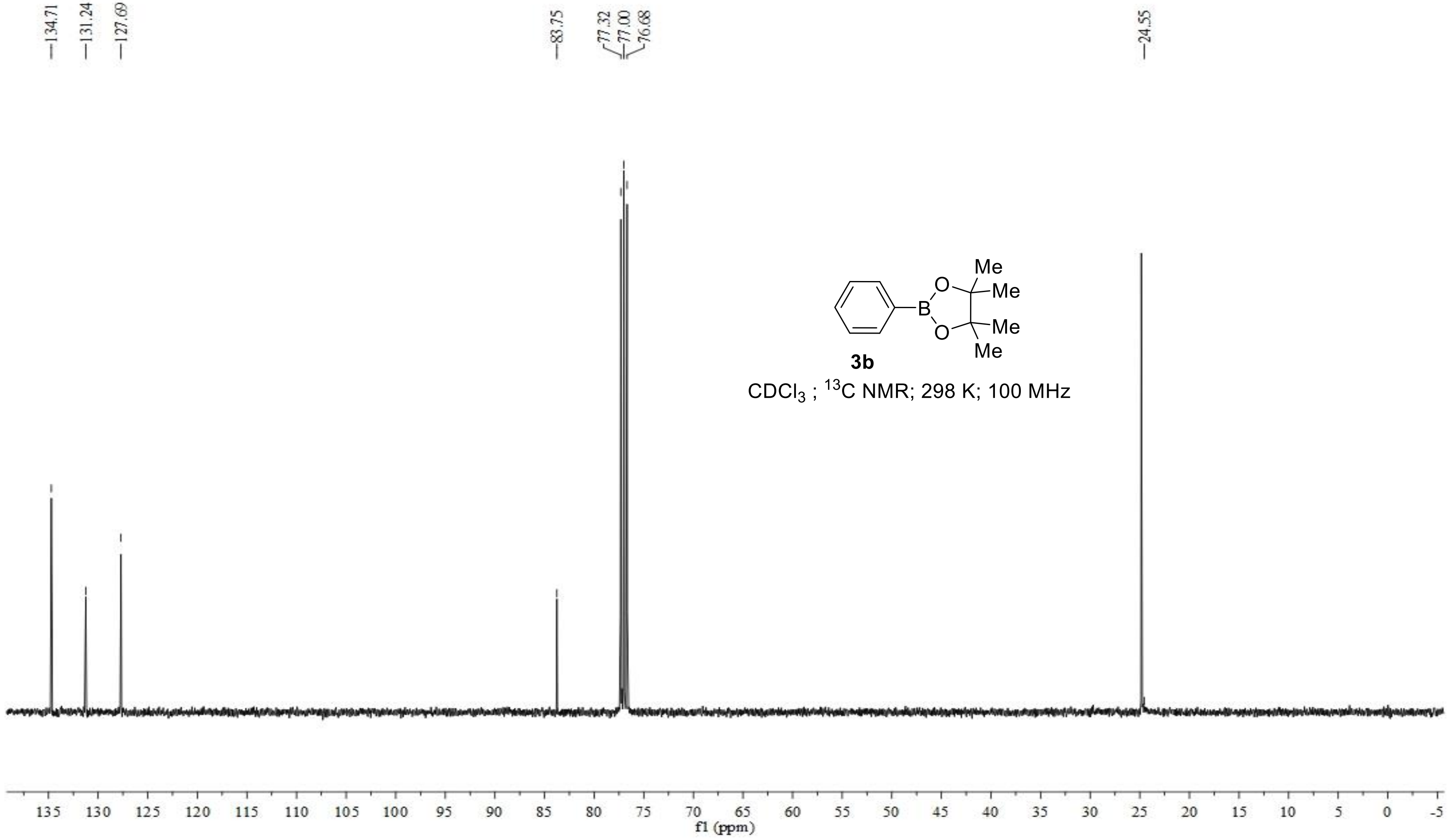
134.71
131.24
127.69
83.75
77.32
77.00
76.68
24.55
Me
O
Me
B
Me
O
Me
3b
CDCl3 ; 13C NMR; 298 K; 100 MHz
135 130 125 120 115 110 105 100 95 90 85 80 75 70 65 60 55 50 45 40 35 30 25 20 15 10 5 0 -5
f1 (ppm)

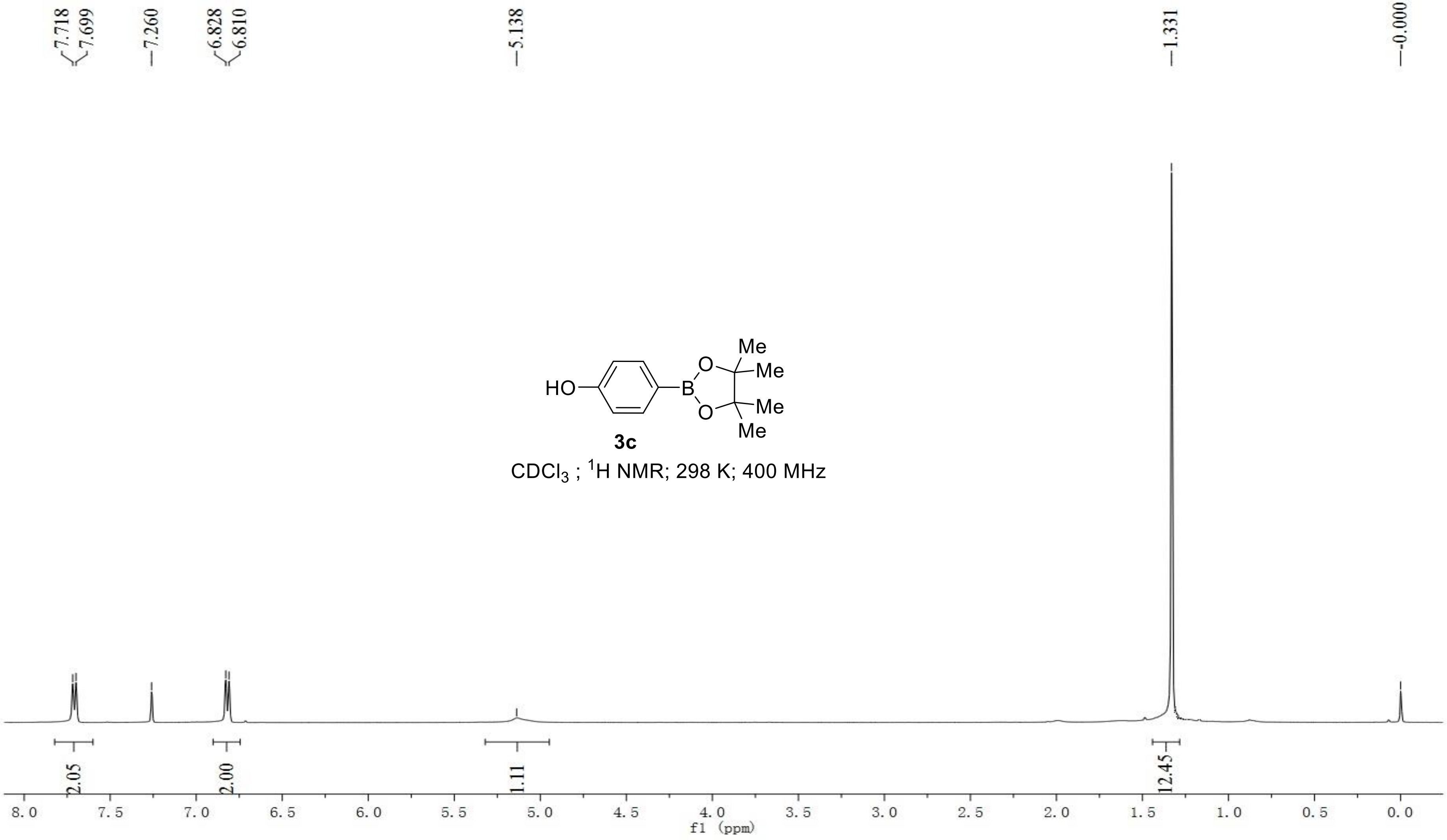
7.718
7.699
7.260
6.828
6.810
5.138
1.331
-0.000
HO
B
O
O
Me
Me
Me
Me
3c
CDCl3 ; 1H NMR; 298 K; 400 MHz
2.05
2.00
1.11
12.45
8.0
7.5
7.0
6.5
6.0
5.5
5.0
4.5
4.0
3.5
3.0
2.5
2.0
1.5
1.0
0.5
0.0
f1 (ppm)

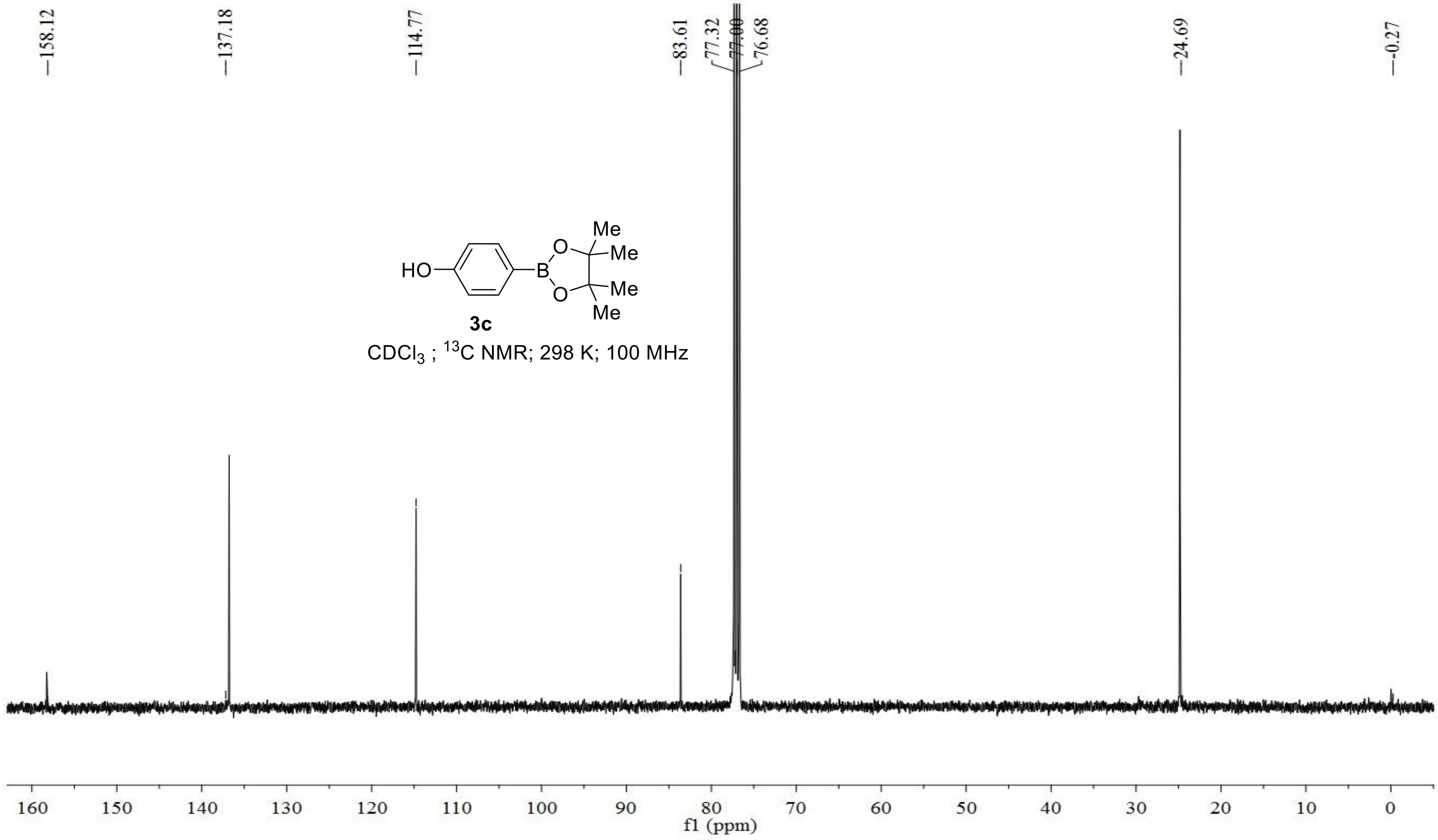

158.12
137.18
114.77
83.61
77.32
77.00
76.68
24.69
-0.27
HO
B
O
O
Me
Me
Me
Me
3c
CDCl$_3$; ^{13}C NMR; 298 K; 100 MHz
160 150 140 130 120 110 100 90 80 70 60 50 40 30 20 10 0
f1 (ppm)

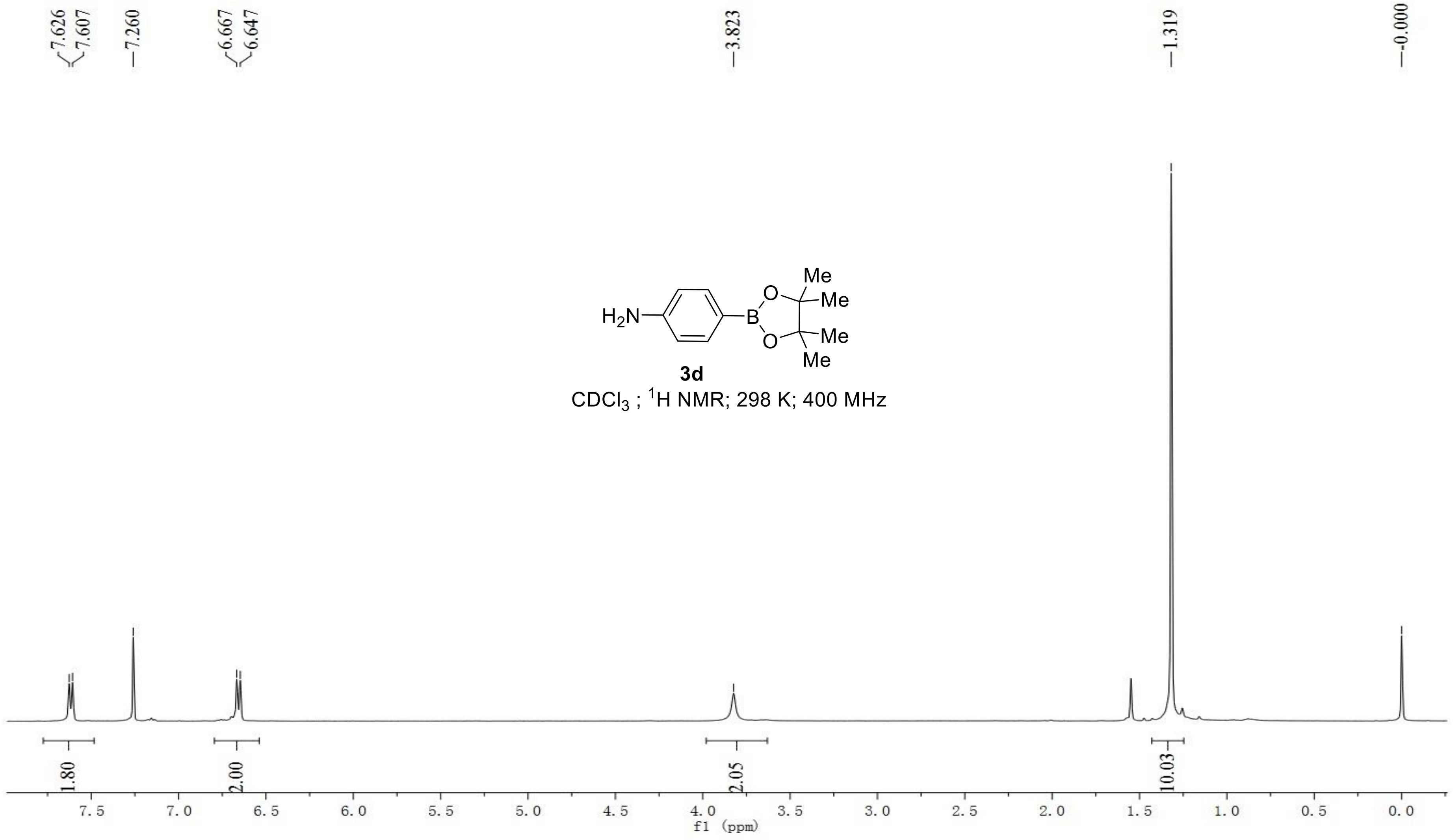
7.626
7.607
7.260
6.667
6.647
3.823
1.319
-0.000
H2N
B
O
Me
3d
CDCl3 ; 1H NMR; 298 K; 400 MHz
1.80
2.00
2.05
10.03
7.5 7.0 6.5 6.0 5.5 5.0 4.5 4.0 3.5 3.0 2.5 2.0 1.5 1.0 0.5 0.0
f1 (ppm)

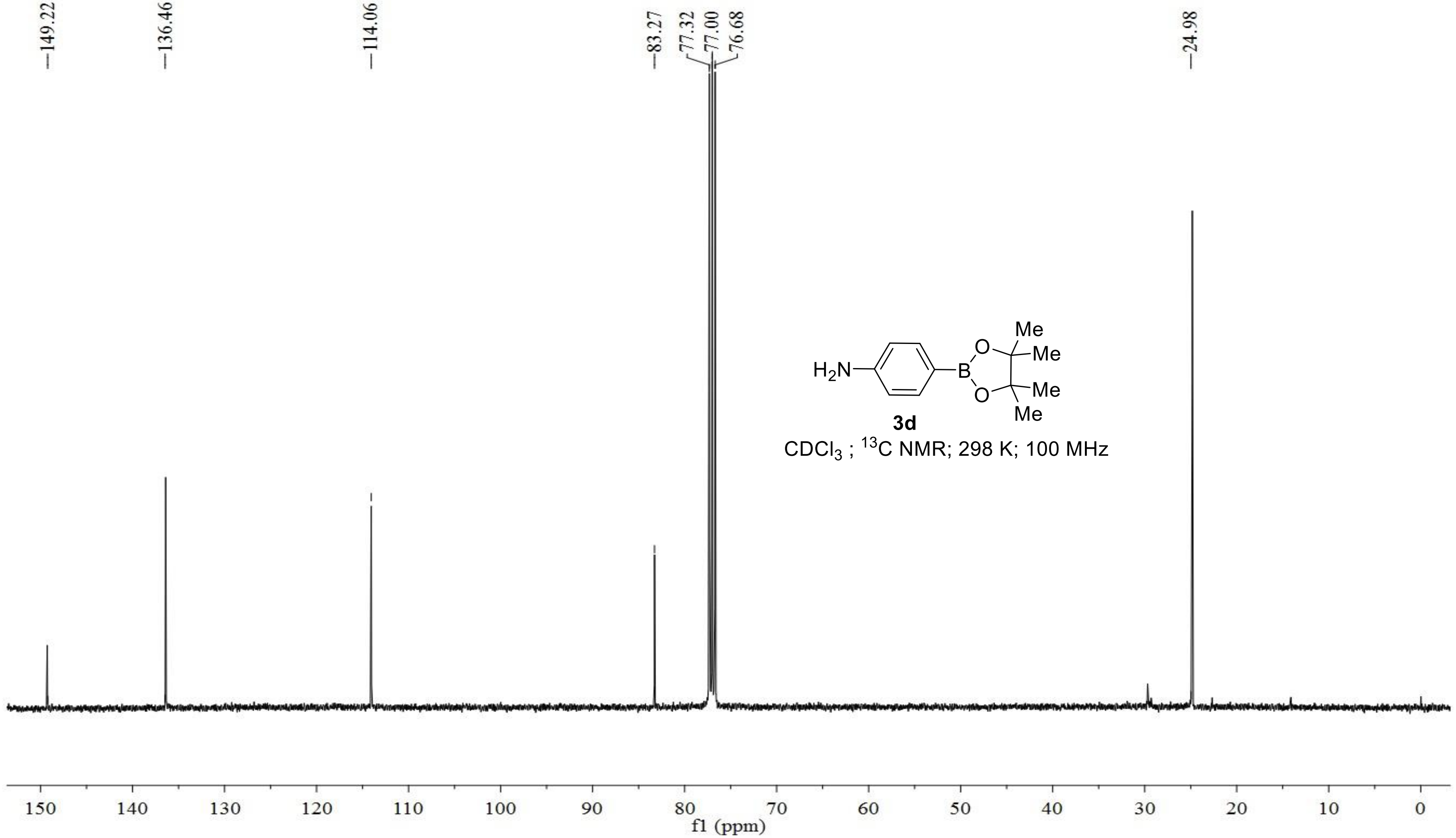
149.22
136.46
114.06
83.27
77.32
77.00
76.68
24.98
H2N
B
O
Me
3d
CDCl3 ; 13C NMR; 298 K; 100 MHz
150
140
130
120
110
100
90
80
70
60
50
40
30
20
10
0
f1 (ppm)

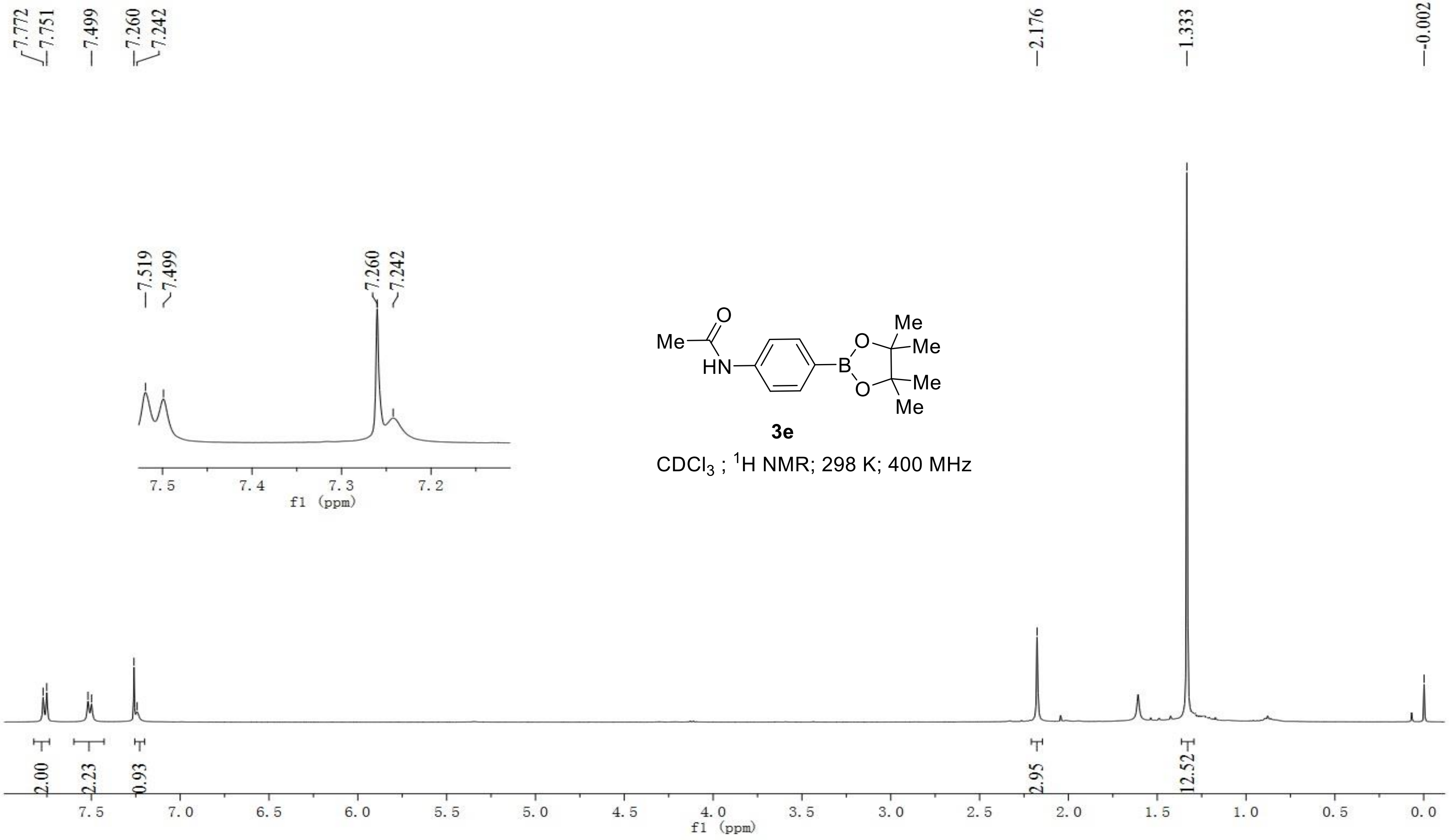
7.772
7.751
7.499
7.260
7.242
2.176
1.333
-0.002
7.519
7.499
7.260
7.242
Me
O
HN
B
O
O
Me
Me
Me
Me
3e
CDCl3 ; 1H NMR; 298 K; 400 MHz
2.00
2.23
0.93
2.95
12.52
f1 (ppm)

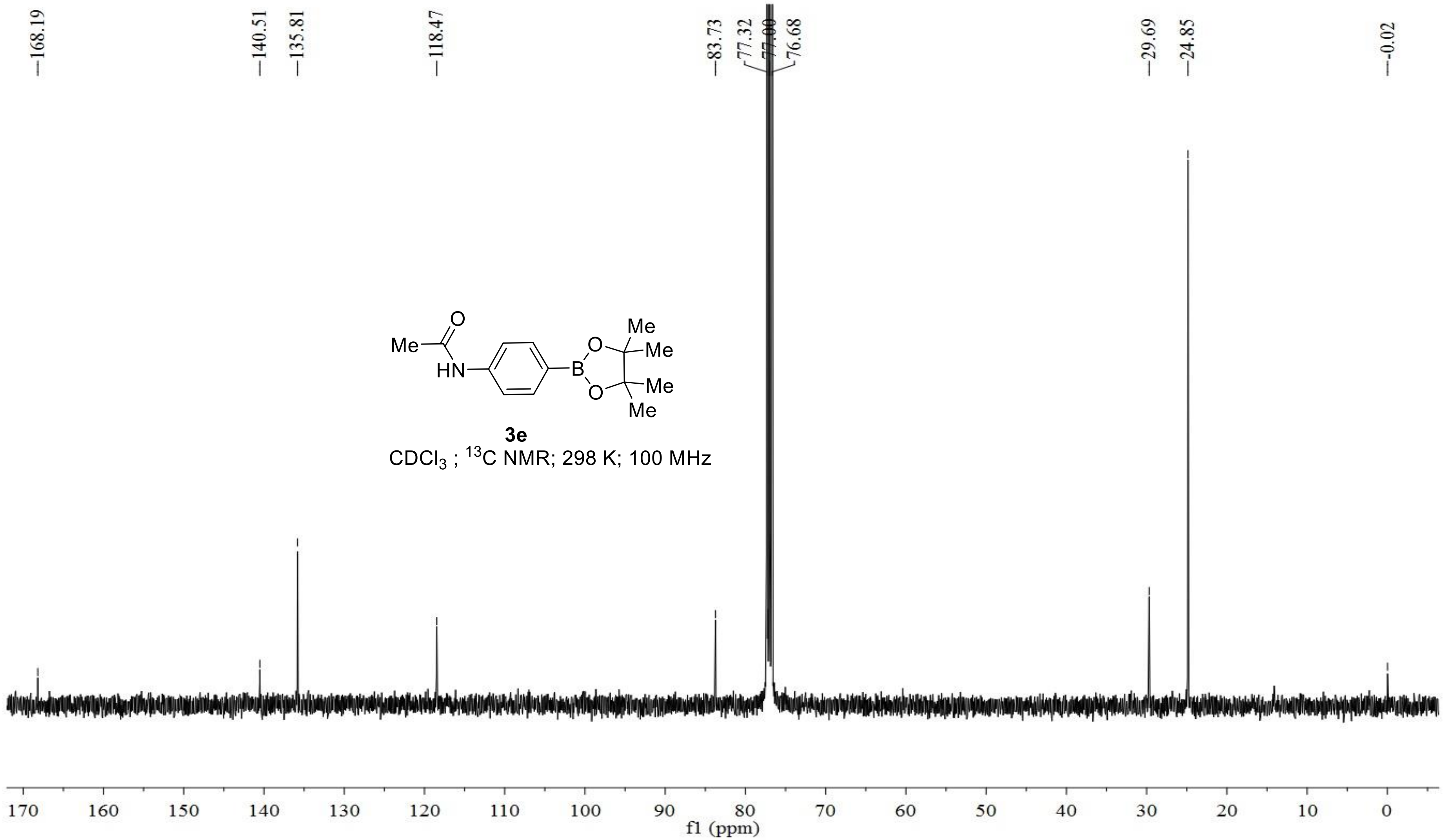

168.19
140.51
135.81
118.47
83.73
77.32
77.00
76.68
29.69
24.85
-0.02
Me
O
HN
B
O
O
Me
Me
Me
Me
3e
$CDCl_3$; ^{13}C NMR; 298 K; 100 MHz
170 160 150 140 130 120 110 100 90 80 70 60 50 40 30 20 10 0
f1 (ppm)

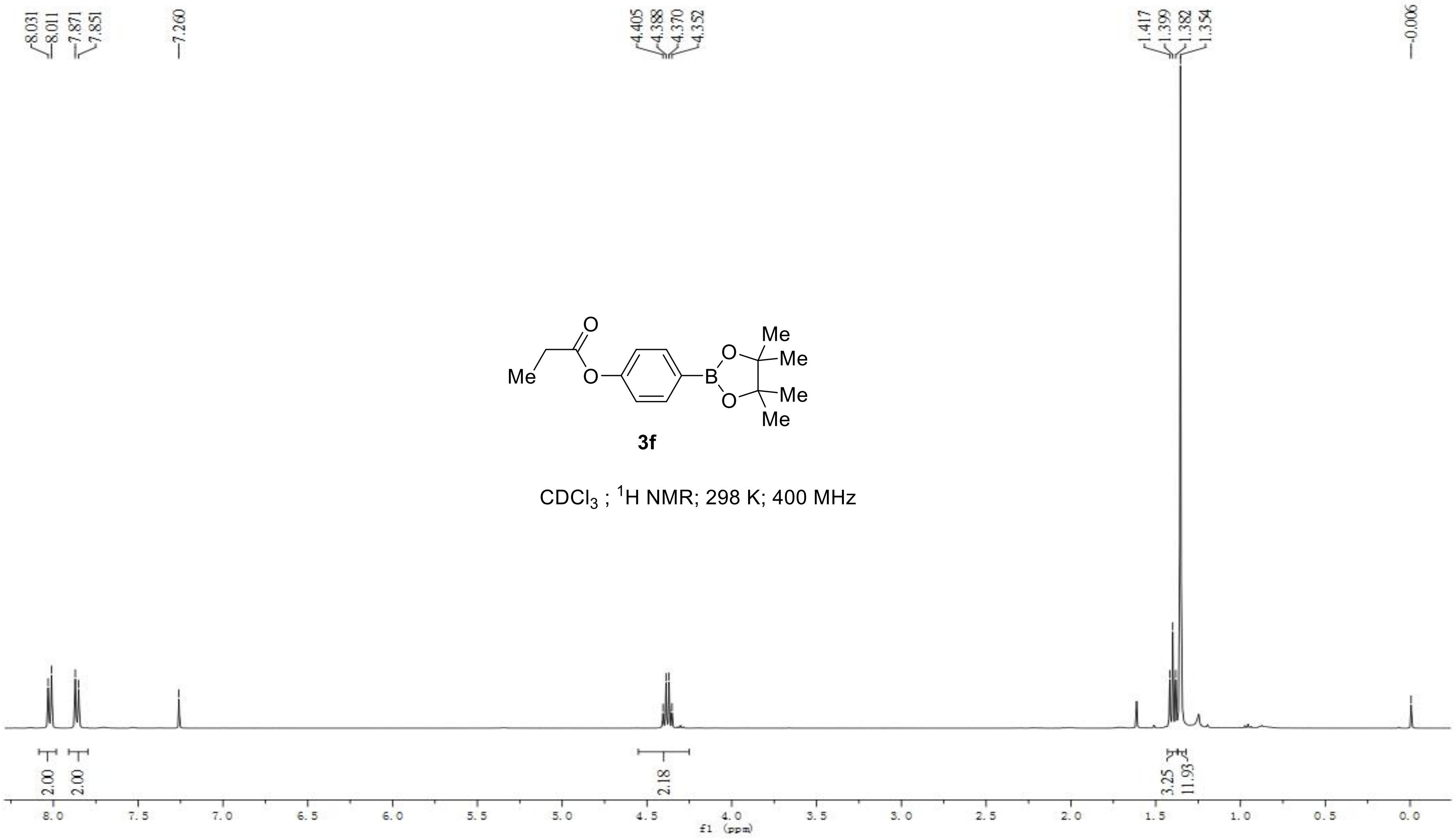
8.031
8.011
7.871
7.851
7.260
4.405
4.388
4.370
4.352
1.417
1.399
1.382
1.354
-0.006
Me
O
O
B
O
O
Me
Me
Me
Me
3f
CDCl3 ; 1H NMR; 298 K; 400 MHz
2.00
2.00
2.18
3.25
11.93
8.0
7.5
7.0
6.5
6.0
5.5
5.0
4.5
4.0
3.5
3.0
2.5
2.0
1.5
1.0
0.5
0.0
f1 (ppm)

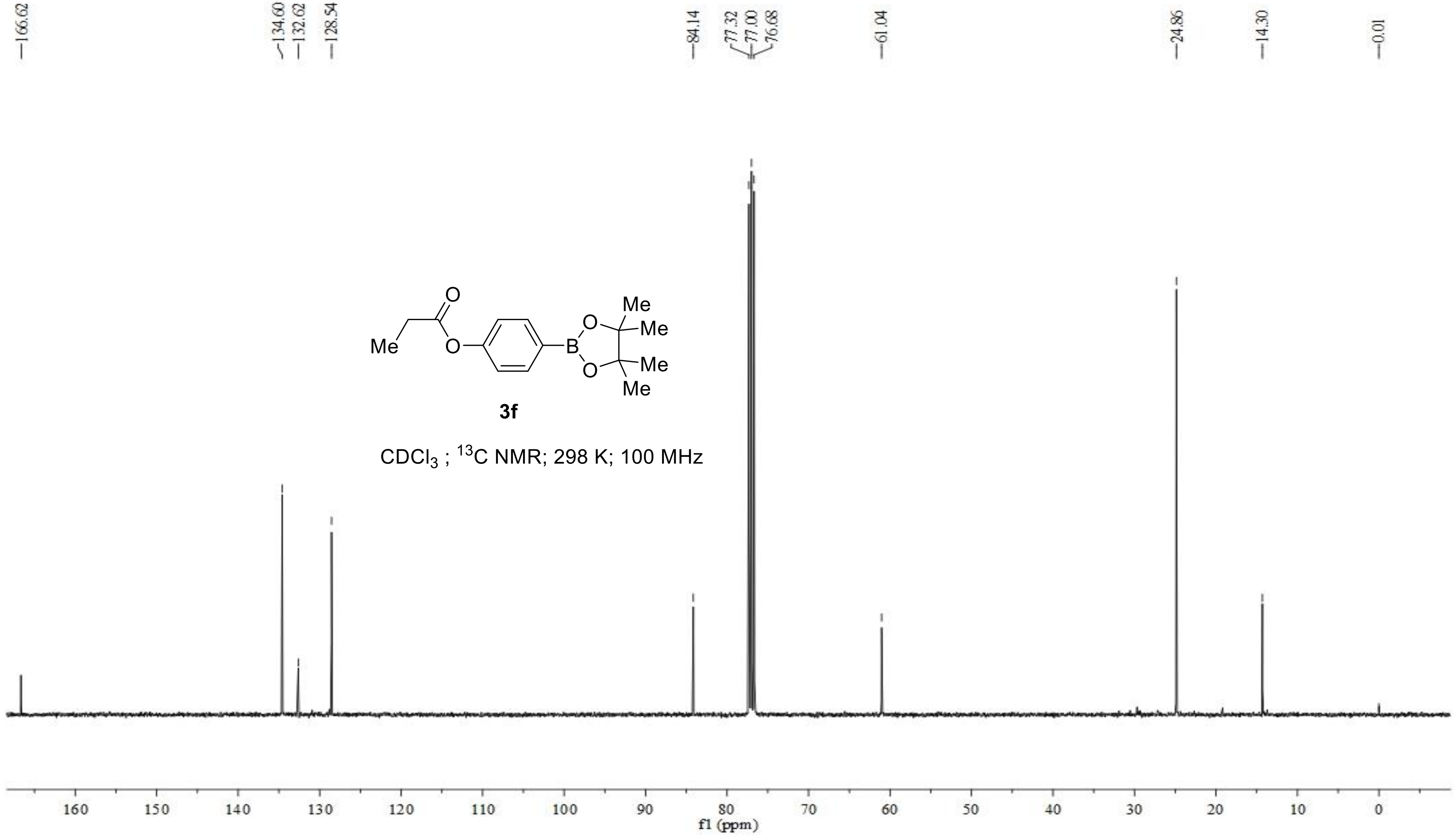
166.62
134.60
132.62
128.54
84.14
77.32
77.00
76.68
61.04
24.86
14.30
0.01
O
Me
O
B
O
O
Me
Me
Me
Me
3f
CDCl3 ; 13C NMR; 298 K; 100 MHz
160 150 140 130 120 110 100 90 80 70 60 50 40 30 20 10 0
f1 (ppm)

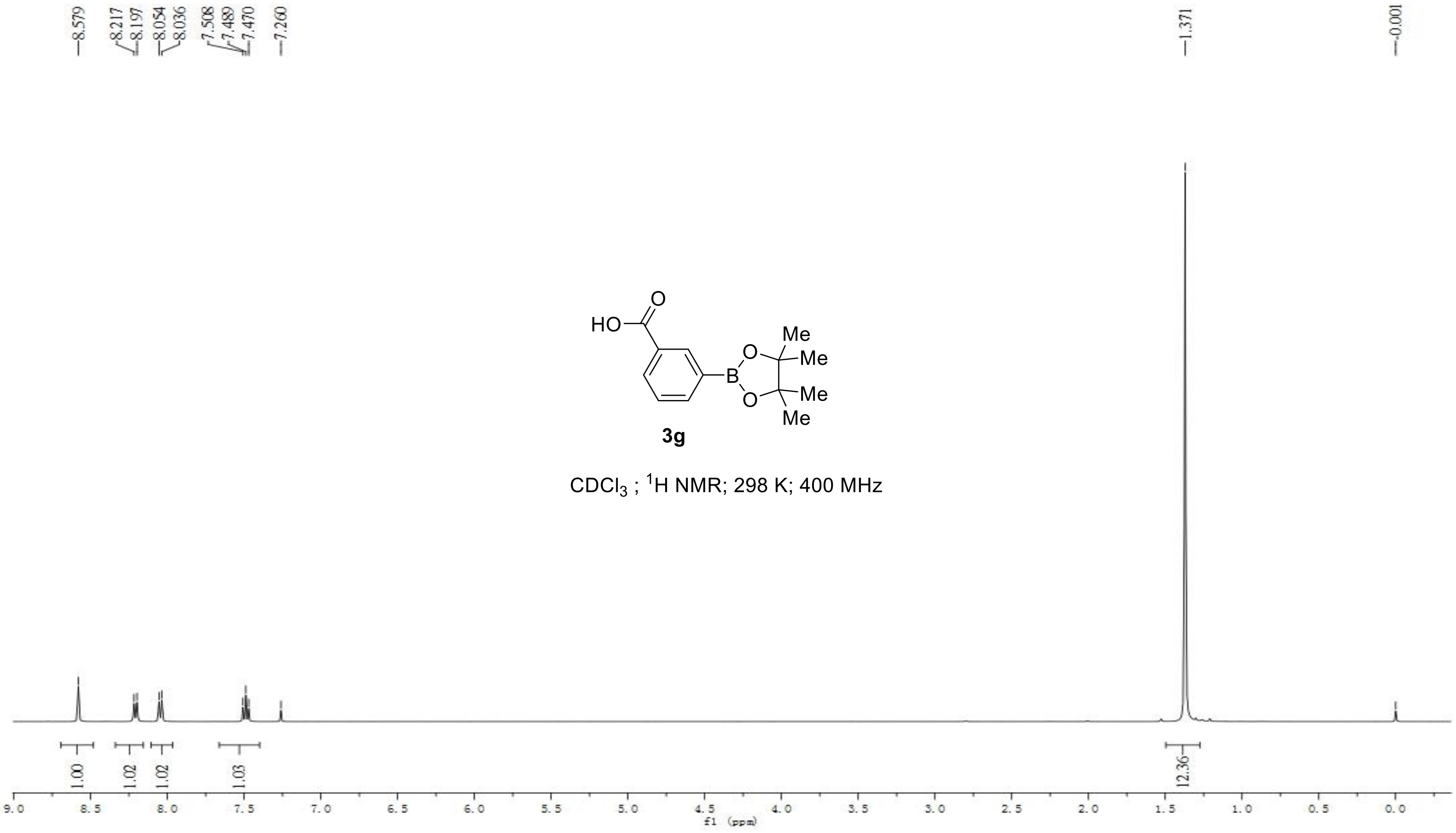
8.579
8.217
8.197
8.054
8.036
7.508
7.489
7.470
7.260
1.371
-0.001
HO
O
B
Me
3g
CDCl3 ; 1H NMR; 298 K; 400 MHz
1.00
1.02
1.02
1.03
12.36
f1 (ppm)

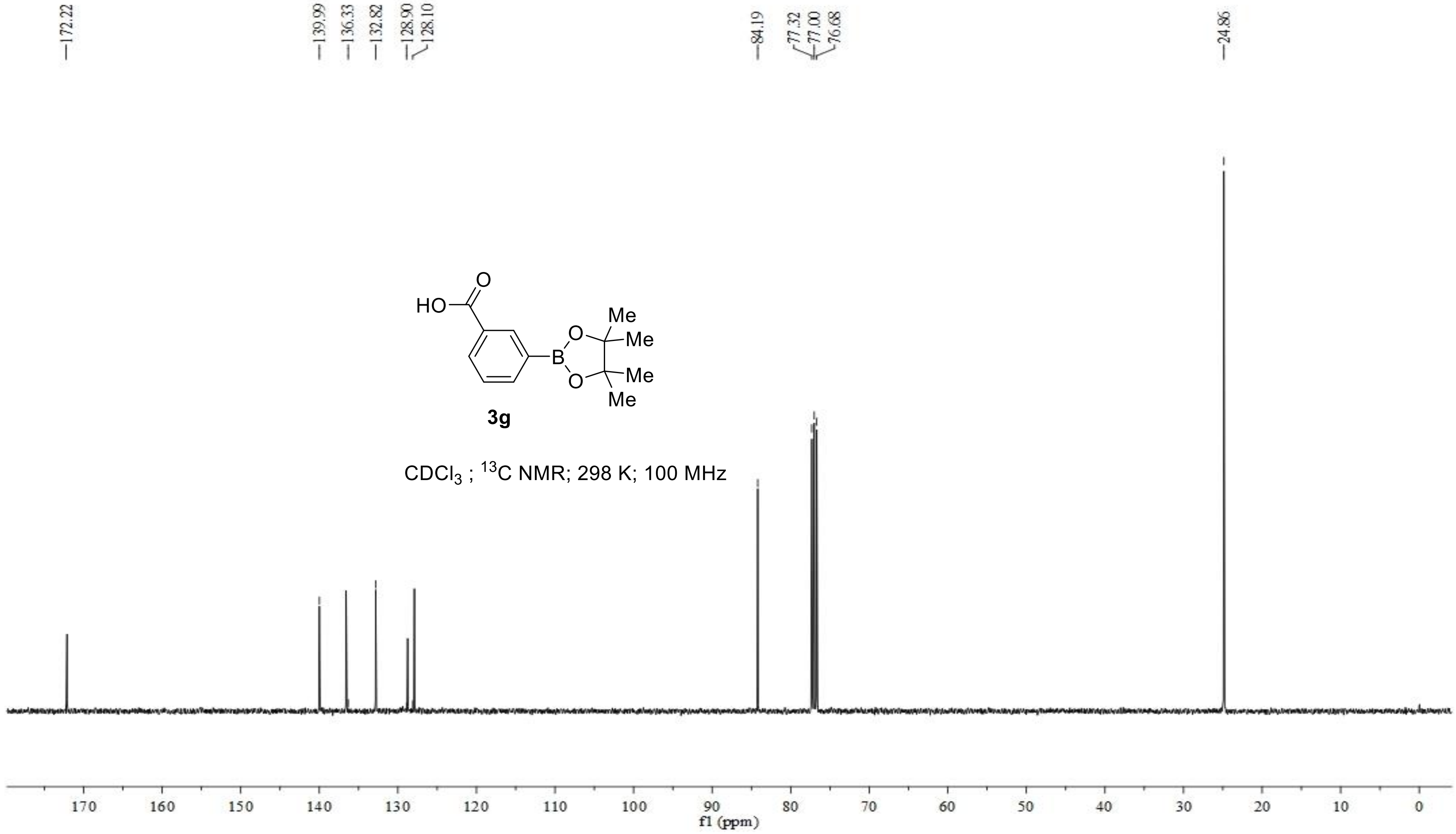
172.22
139.99
136.33
132.82
128.90
128.10
84.19
77.32
77.00
76.68
24.86
HO
O
B
O
O
Me
Me
Me
Me
3g
CDCl3 ; 13C NMR; 298 K; 100 MHz
170 160 150 140 130 120 110 100 90 80 70 60 50 40 30 20 10 0
f1 (ppm)

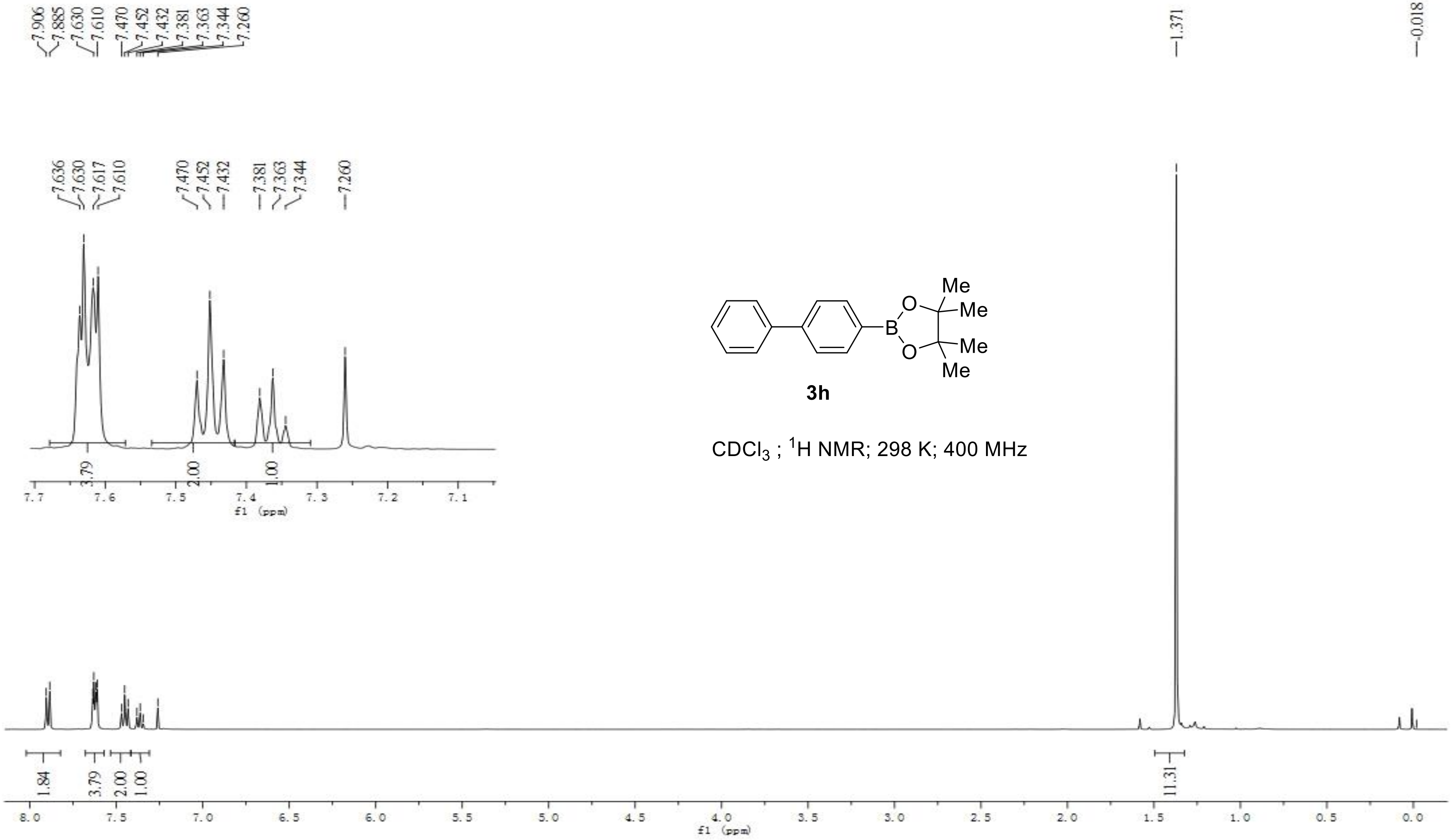
7.906
7.885
7.630
7.610
7.470
7.452
7.432
7.381
7.363
7.344
7.260
1.371
-0.018
7.636
7.630
7.617
7.610
7.470
7.452
7.432
7.381
7.363
7.344
7.260
3.79
2.00
1.00
f1 (ppm)
Me
Me
Me
Me
O
O
B
3h
CDCl3 ; 1H NMR; 298 K; 400 MHz
1.84
3.79
2.00
1.00
11.31
f1 (ppm)

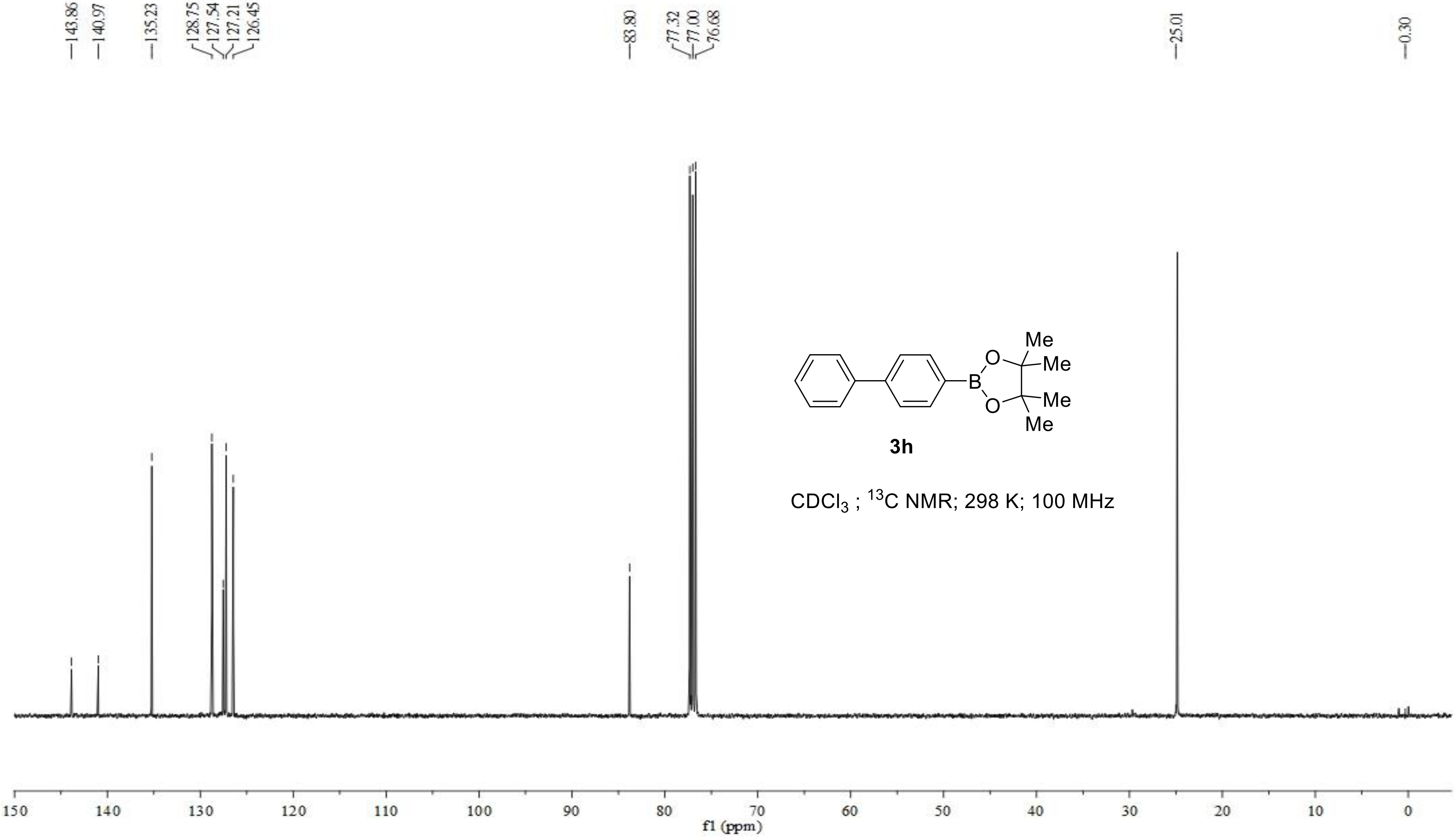
143.86
140.97
135.23
128.75
127.54
127.21
126.45
83.80
77.32
77.00
76.68
25.01
0.30
Me
O
Me
B
Me
O
Me
3h
CDCl3 ; 13C NMR; 298 K; 100 MHz
150
140
130
120
110
100
90
80
70
60
50
40
30
20
10
0
f1 (ppm)

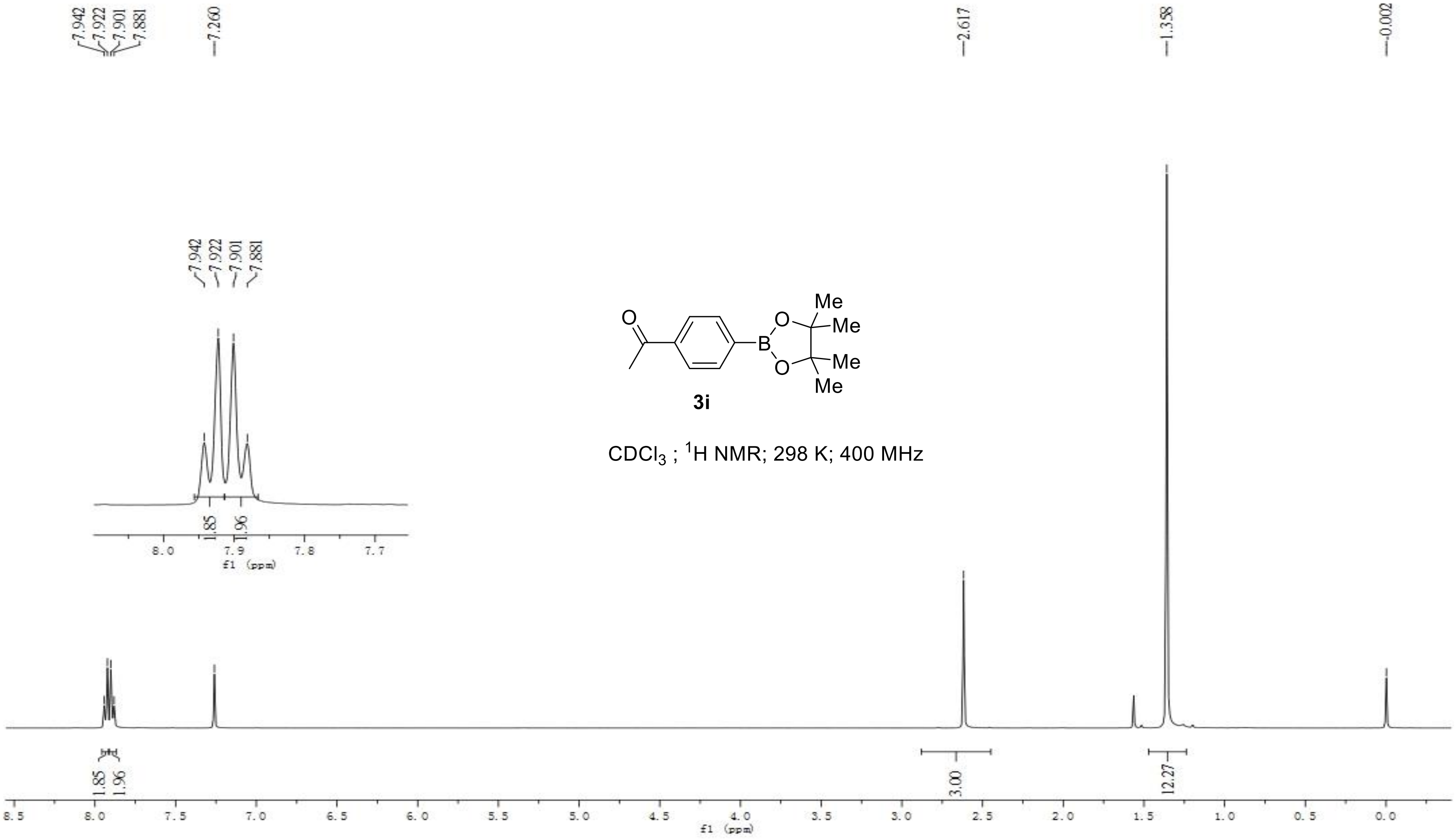
7.942
7.922
7.901
7.881
7.260
2.617
1.358
-0.002
Me
Me
Me
Me
O
O
B
O
3i
CDCl3 ; 1H NMR; 298 K; 400 MHz
1.85
1.96
3.00
12.27
8.0
7.9
7.8
7.7
f1 (ppm)
8.5
7.5
7.0
6.5
6.0
5.5
5.0
4.5
4.0
3.5
3.0
2.5
2.0
1.5
1.0
0.5
0.0

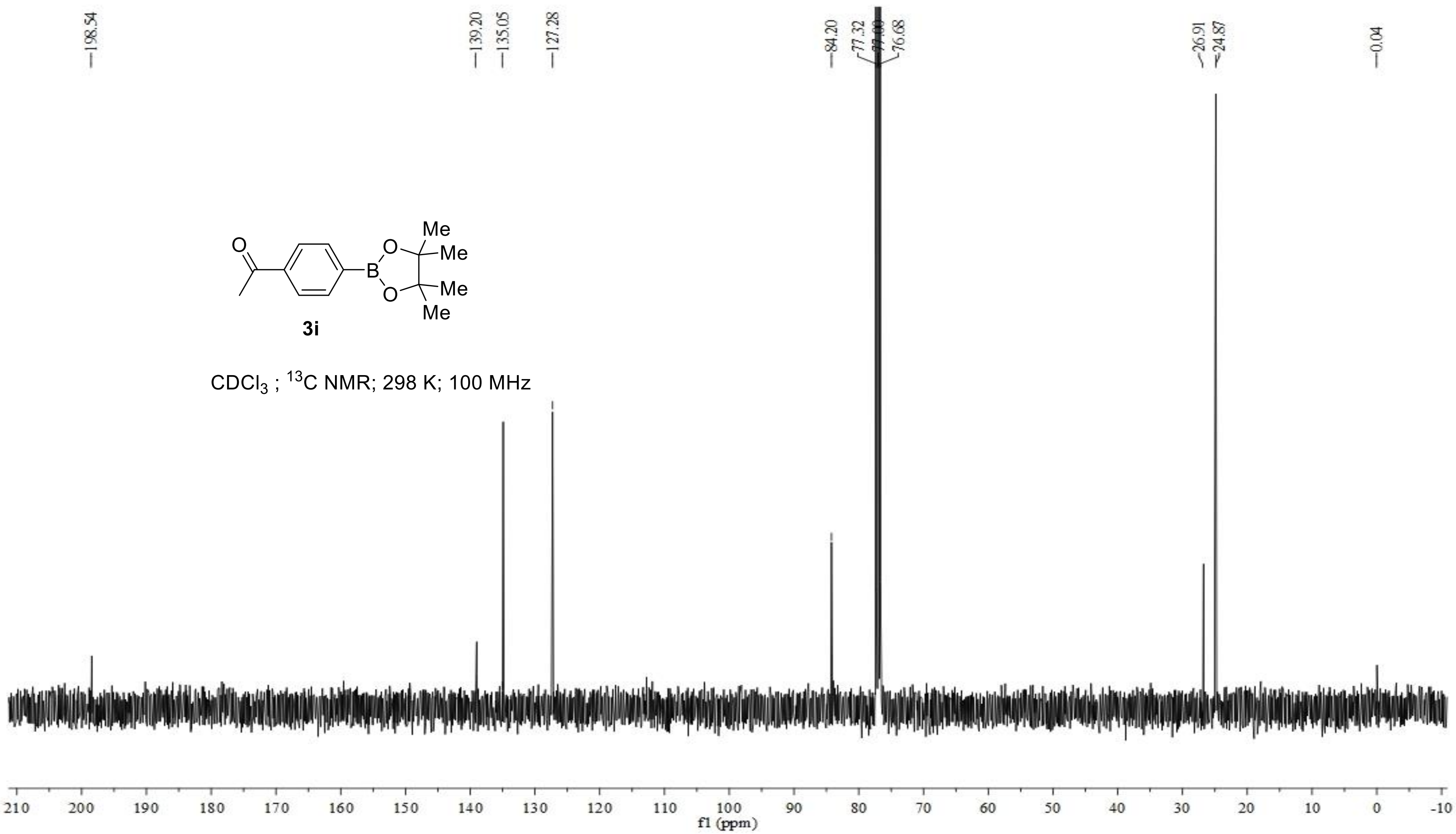
198.54
139.20
135.05
127.28
84.20
77.32
77.00
76.68
26.91
24.87
0.04
Me
Me
Me
Me
O
O
O
B
3i
CDCl3 ; 13C NMR; 298 K; 100 MHz
210 200 190 180 170 160 150 140 130 120 110 100 90 80 70 60 50 40 30 20 10 0 -10
f1 (ppm)

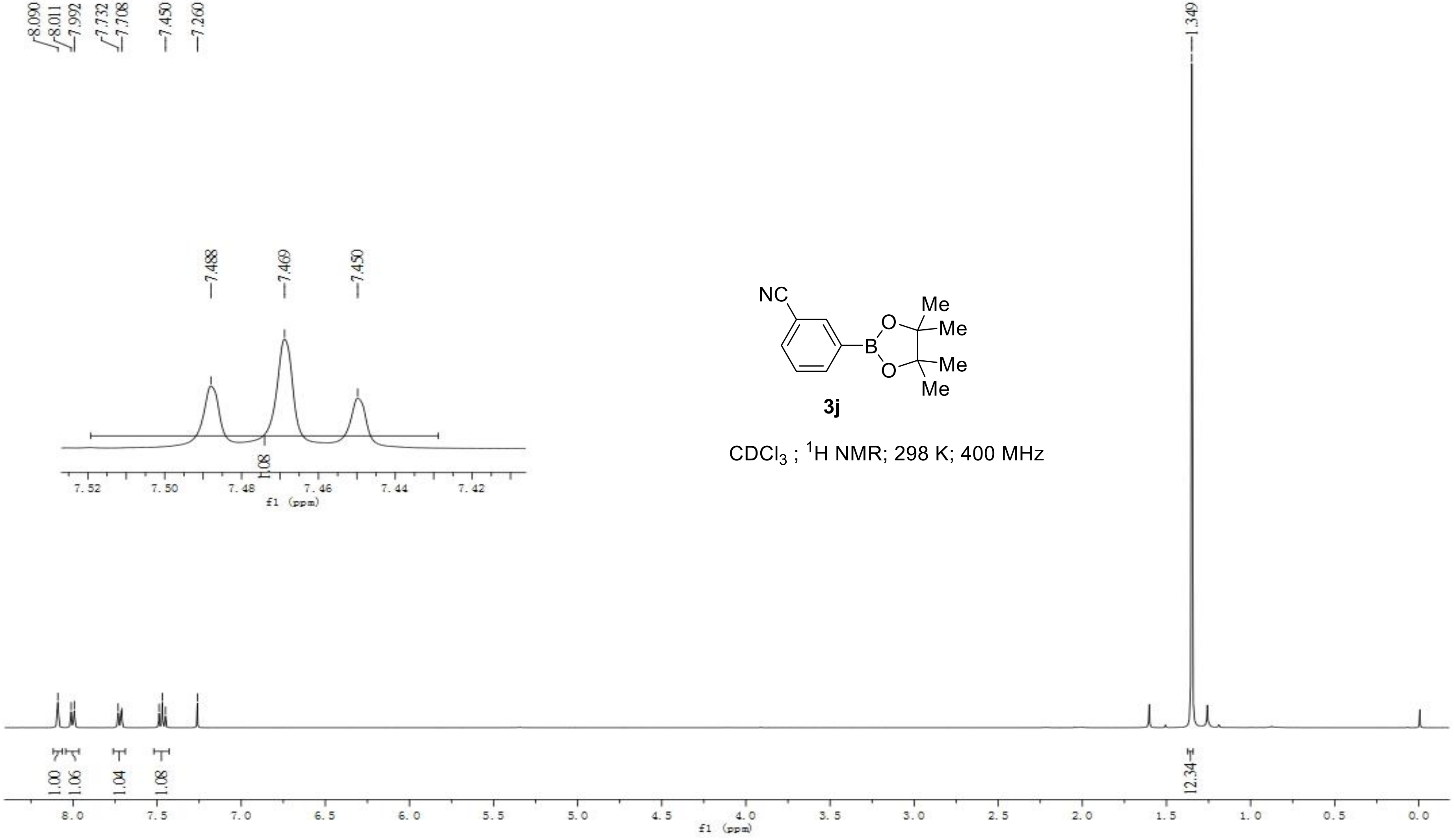
8.090
8.011
7.992
7.732
7.708
7.450
7.260
1.349
7.488
7.469
7.450
1.08
NC
O
B
O
Me
Me
Me
Me
3j
CDCl3 ; 1H NMR; 298 K; 400 MHz
1.00
1.06
1.04
1.08
12.34
f1 (ppm)

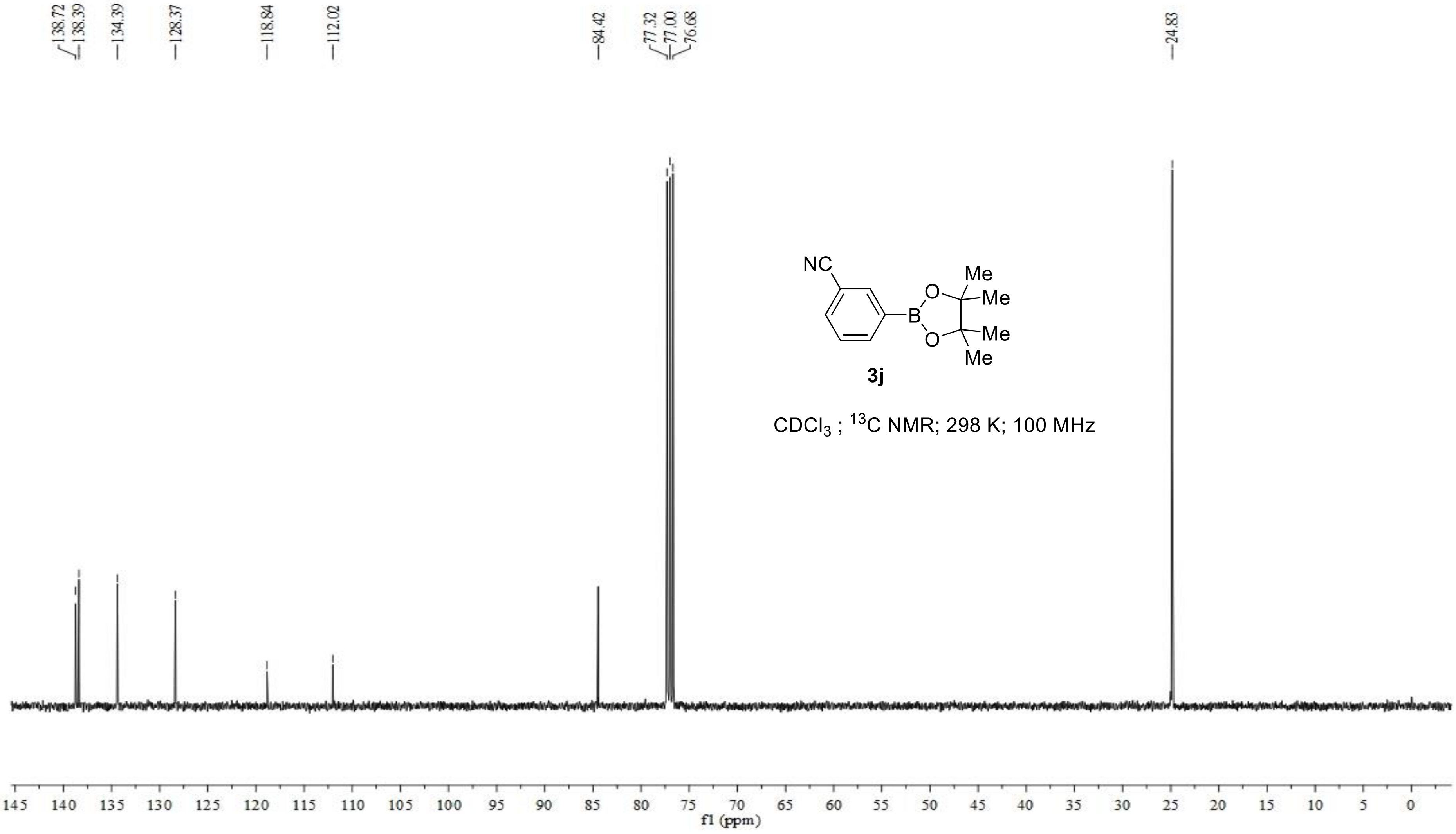
138.72
138.39
134.39
128.37
118.84
112.02
84.42
77.32
77.00
76.68
24.83
NC
Me
Me
Me
Me
O
B
O
3j
CDCl3 ; 13C NMR; 298 K; 100 MHz
145 140 135 130 125 120 115 110 105 100 95 90 85 80 75 70 65 60 55 50 45 40 35 30 25 20 15 10 5 0
f1 (ppm)

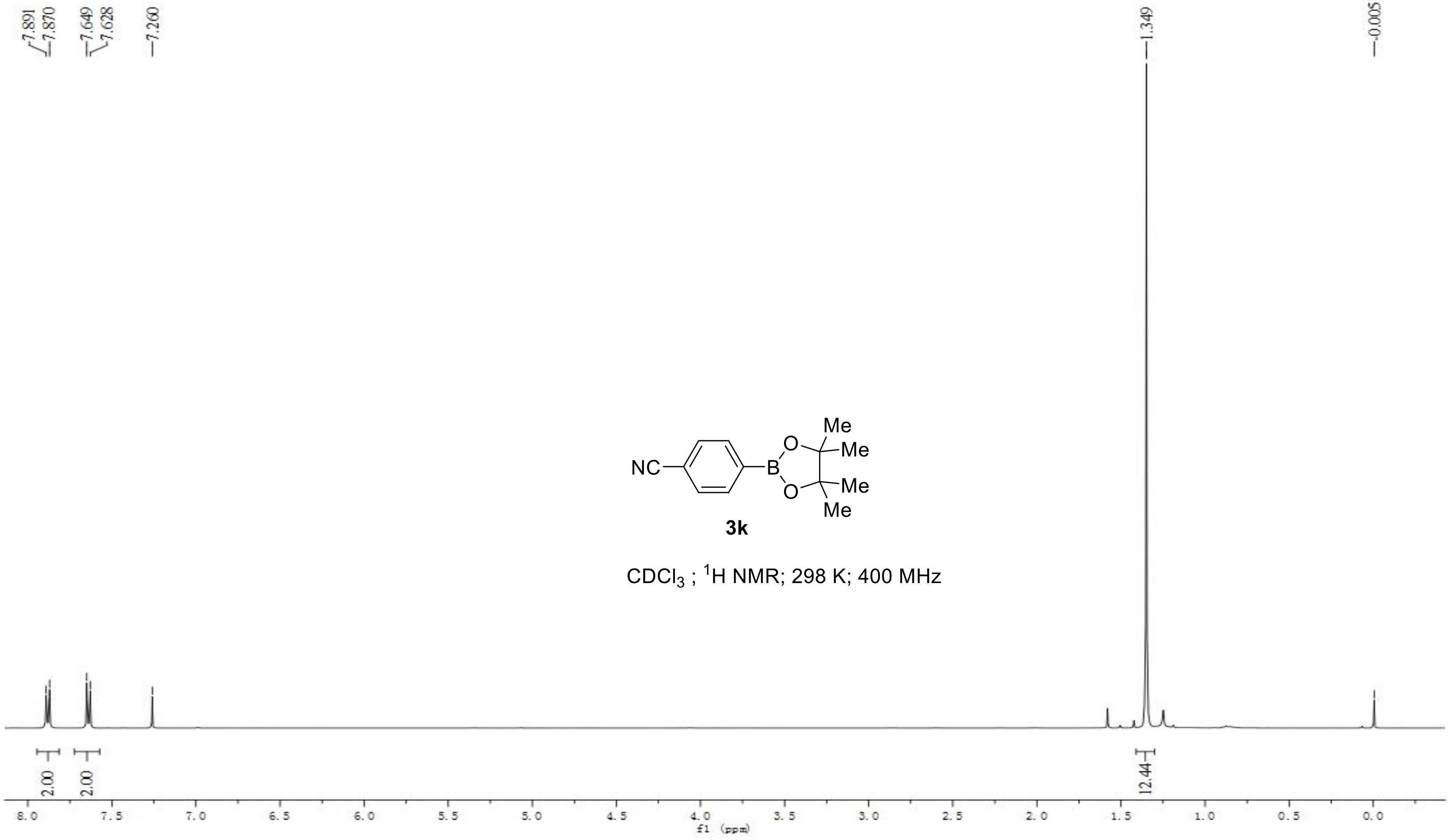
7.891
7.870
7.649
7.628
7.260
1.349
-0.005
NC
B
O
O
Me
Me
Me
Me
3k
CDCl3 ; 1H NMR; 298 K; 400 MHz
2.00
2.00
12.44
8.0
7.5
7.0
6.5
6.0
5.5
5.0
4.5
4.0
3.5
3.0
2.5
2.0
1.5
1.0
0.5
0.0
f1 (ppm)

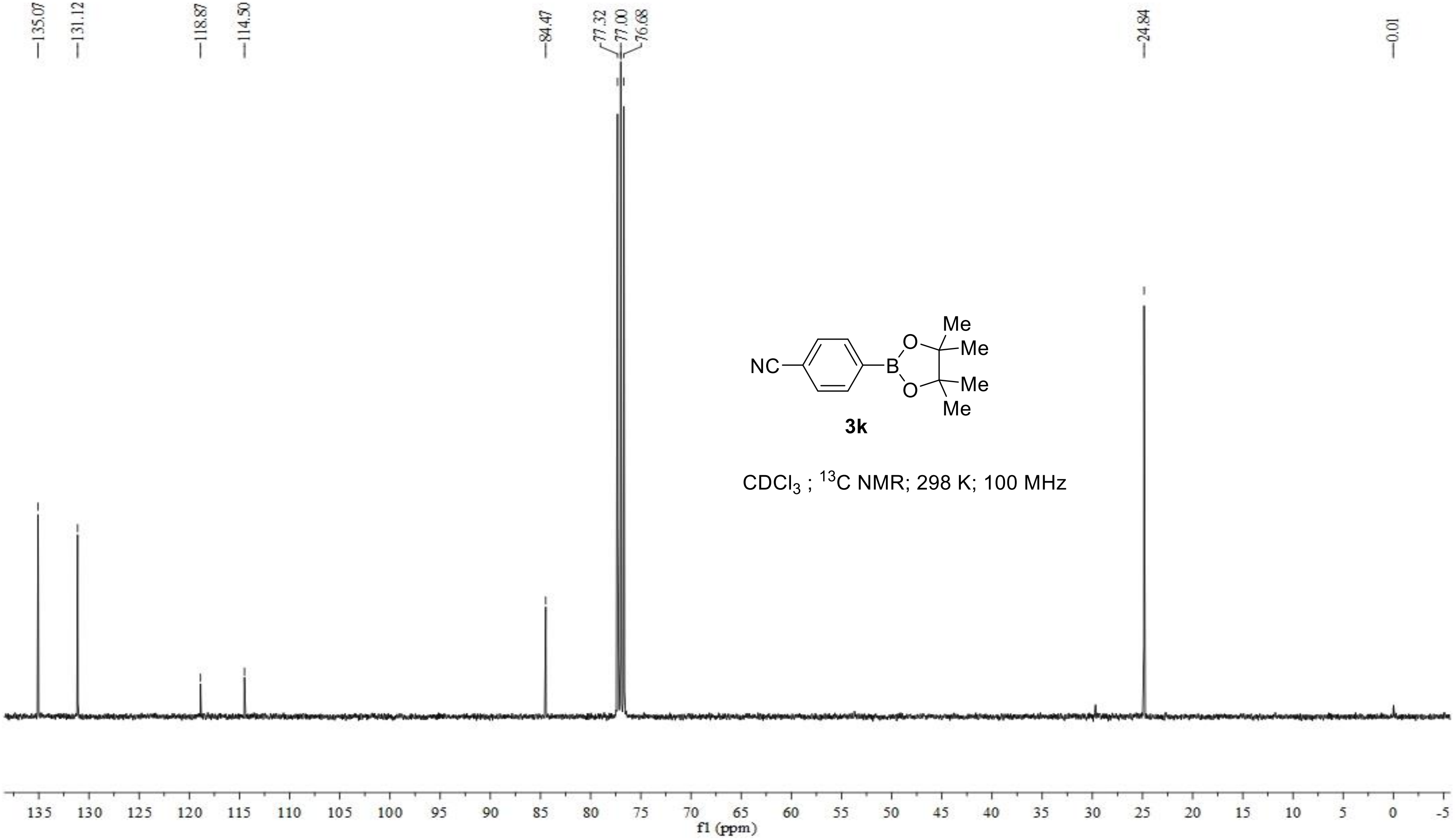
135.07
131.12
118.87
114.50
84.47
77.32
77.00
76.68
24.84
0.01
NC
B
O
O
Me
Me
Me
Me
3k
CDCl3 ; 13C NMR; 298 K; 100 MHz
135 130 125 120 115 110 105 100 95 90 85 80 75 70 65 60 55 50 45 40 35 30 25 20 15 10 5 0 -5
f1 (ppm)

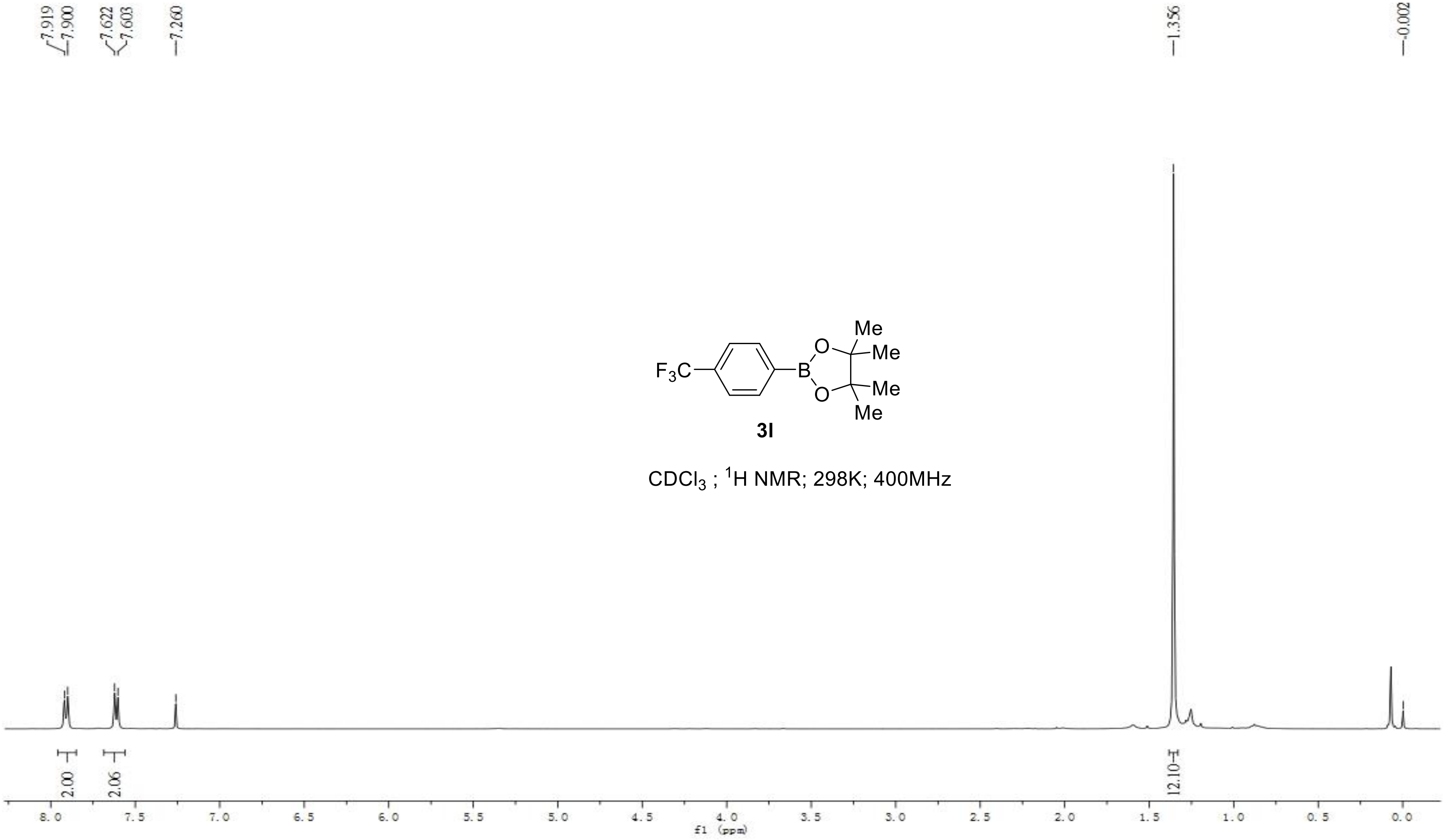

7.919
7.900
7.622
7.603
7.260
1.356
-0.002
F3C
B
O
O
Me
Me
Me
Me
3l
CDCl3 ; 1H NMR; 298K; 400MHz
2.00
2.06
12.10
8.0
7.5
7.0
6.5
6.0
5.5
5.0
4.5
4.0
3.5
3.0
2.5
2.0
1.5
1.0
0.5
0.0
f1 (ppm)

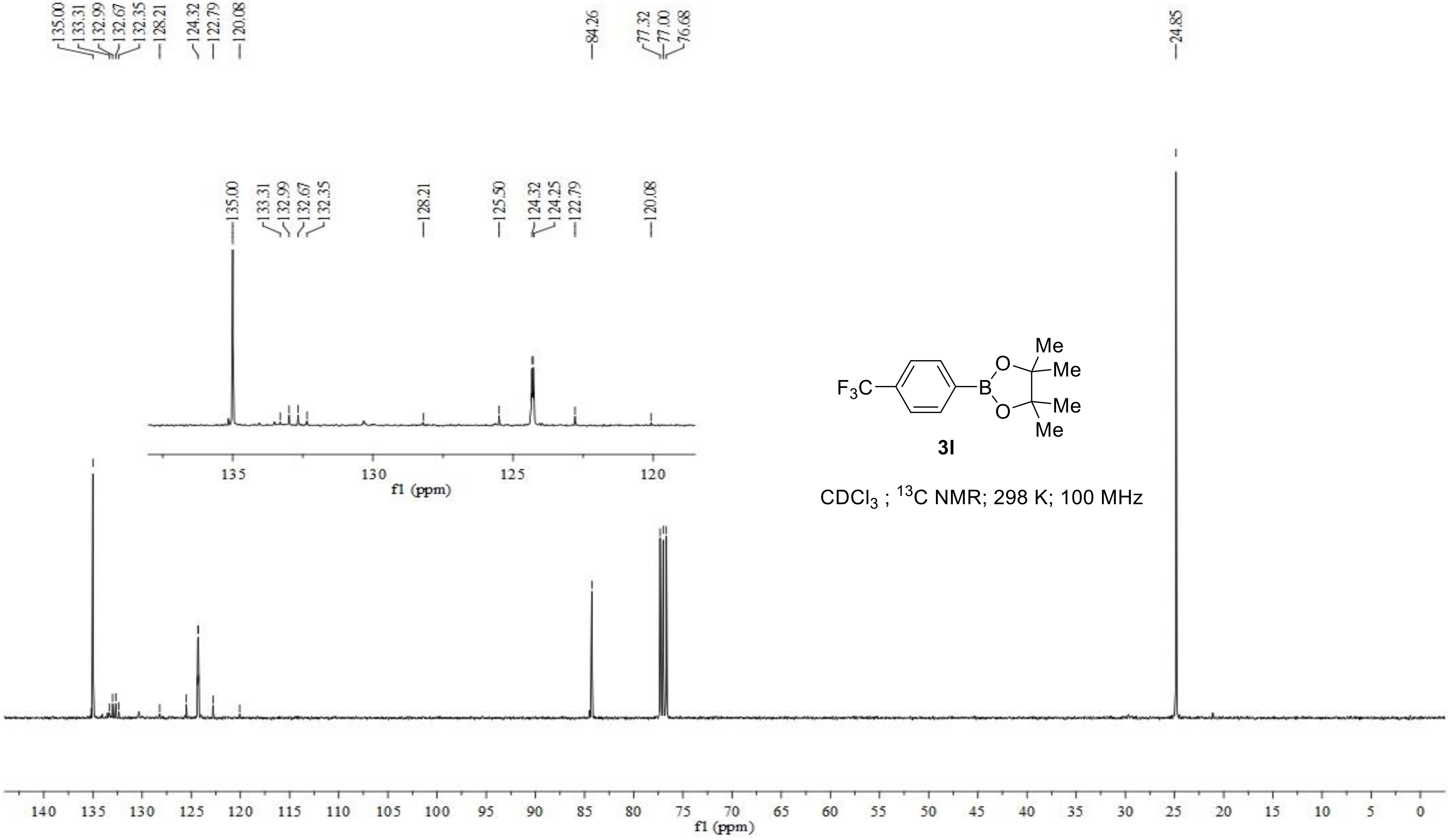
135.00
133.31
132.99
132.67
132.35
128.21
124.32
122.79
120.08
84.26
77.32
77.00
76.68
24.85
135.00
133.31
132.99
132.67
132.35
128.21
125.50
124.32
124.25
122.79
120.08
135
130
125
120
f1 (ppm)
F3C
B
O
O
Me
Me
Me
Me
3l
CDCl3 ; 13C NMR; 298 K; 100 MHz
140
135
130
125
120
115
110
105
100
95
90
85
80
75
70
65
60
55
50
45
40
35
30
25
20
15
10
5
0
f1 (ppm)

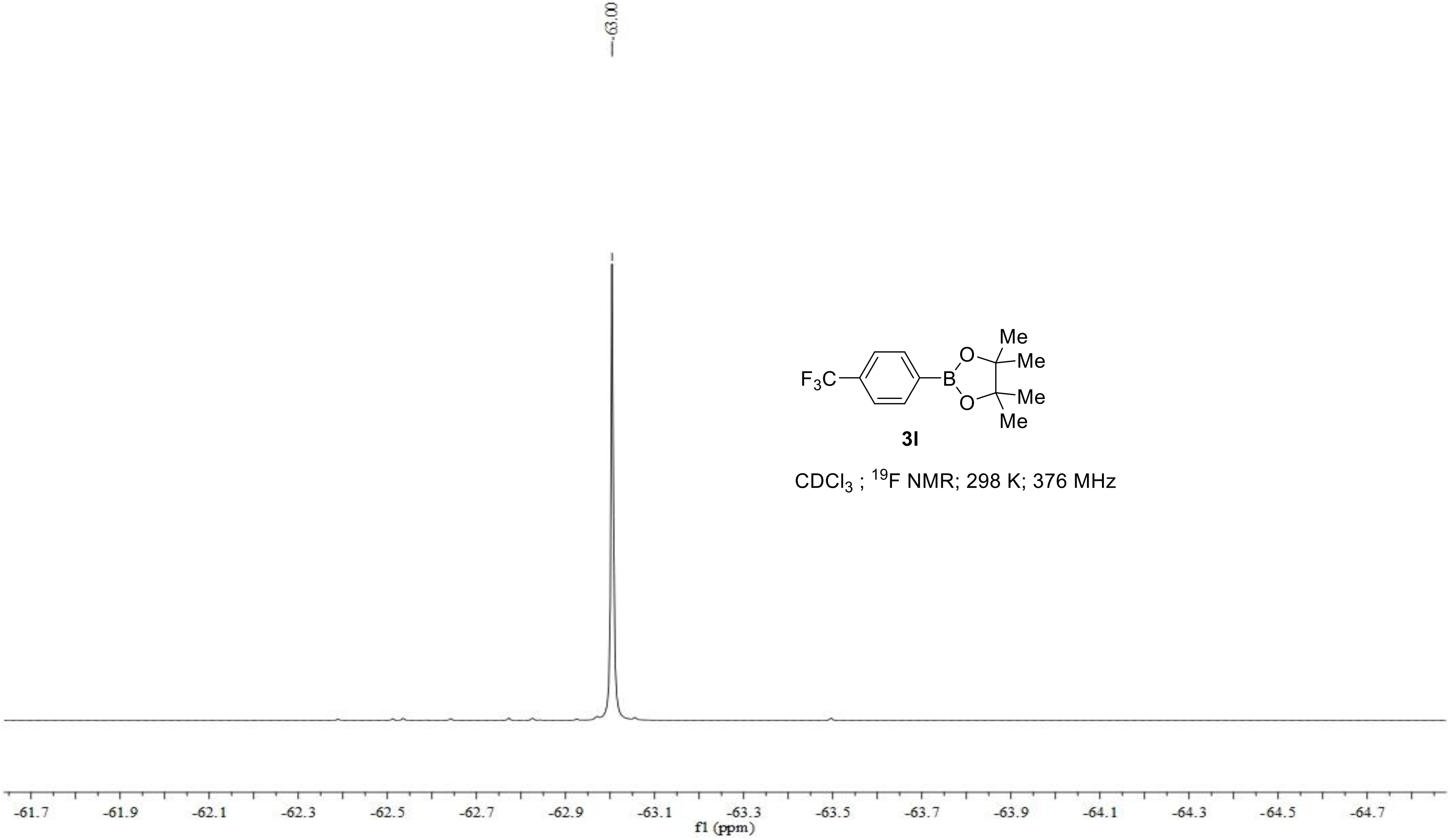
-63.00
F3C
B
O
O
Me
Me
Me
Me
3l
CDCl3 ; 19F NMR; 298 K; 376 MHz
-61.7
-61.9
-62.1
-62.3
-62.5
-62.7
-62.9
-63.1
-63.3
-63.5
-63.7
-63.9
-64.1
-64.3
-64.5
-64.7
f1 (ppm)

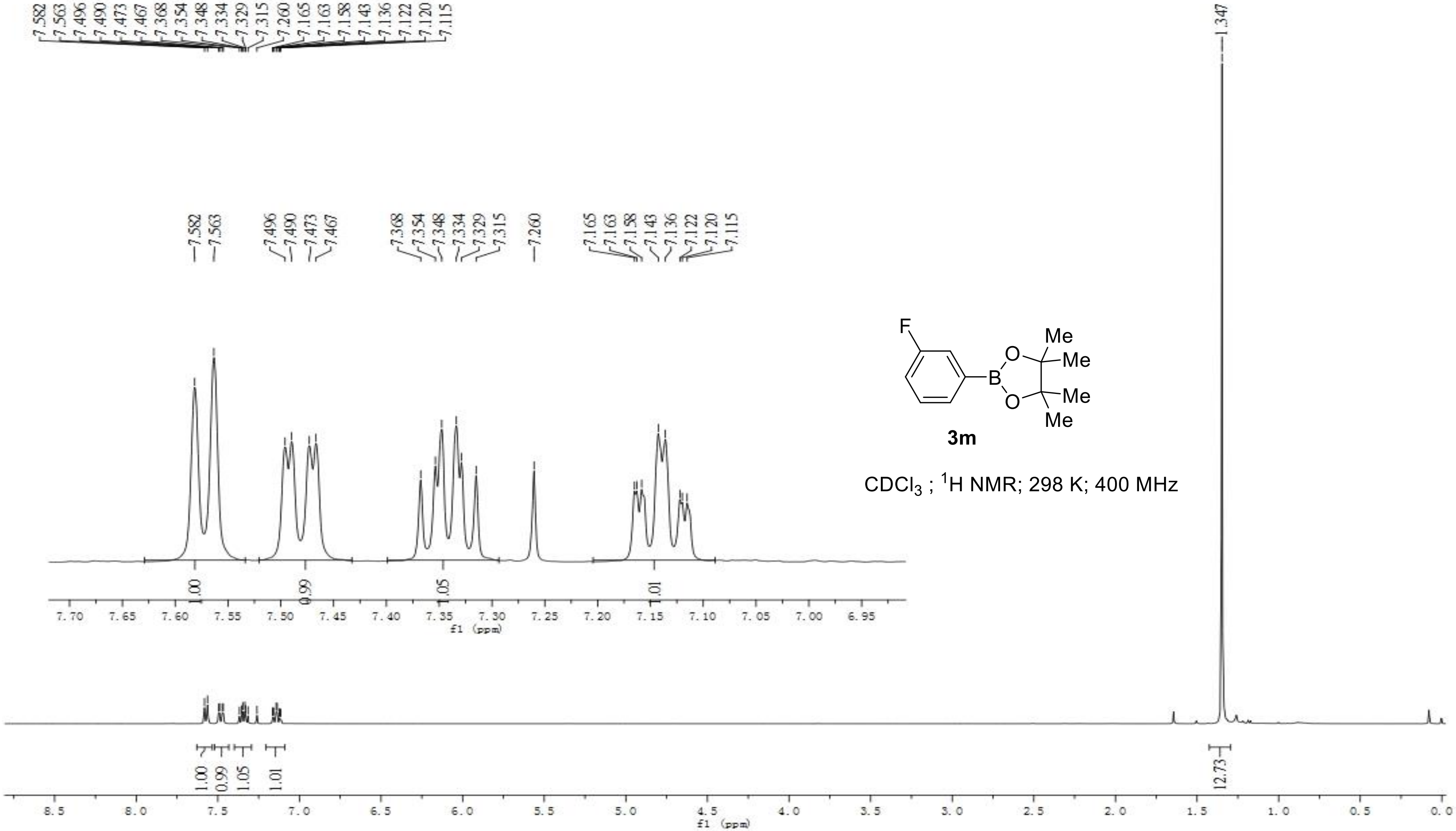
7.582 7.563 7.496 7.490 7.473 7.467 7.368 7.354 7.348 7.334 7.329 7.315 7.260 7.165 7.163 7.158 7.143 7.136 7.122 7.120 7.115
1.347
F
B
O
O
Me
Me
Me
Me
3m
CDCl3 ; 1H NMR; 298 K; 400 MHz
1.00
0.99
1.05
1.01
12.73
f1 (ppm)

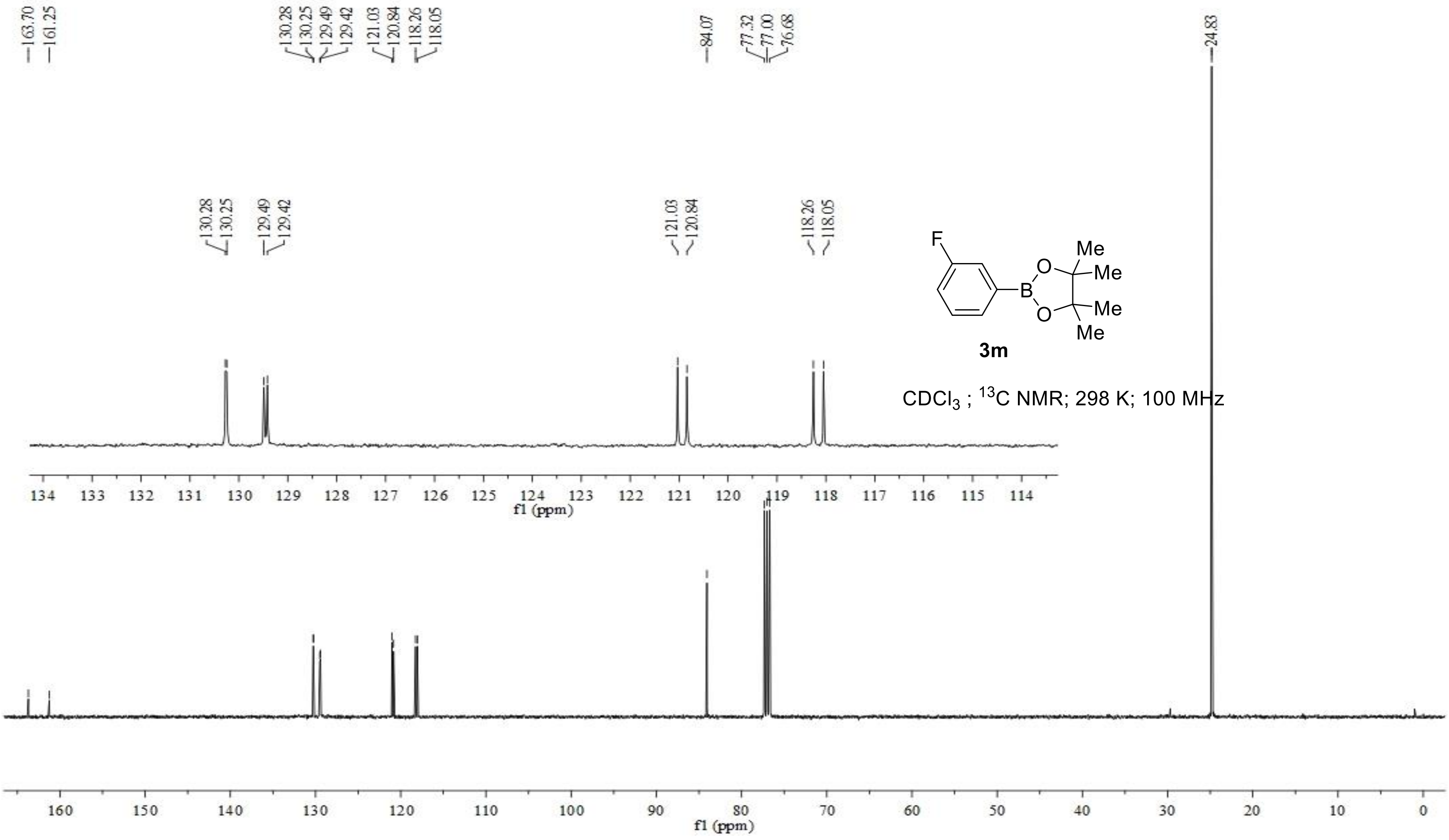

163.70
161.25
130.28
130.25
129.49
129.42
121.03
120.84
118.26
118.05
84.07
77.32
77.00
76.68
24.83
F
B
O
O
Me
Me
Me
Me
3m
CDCl3 ; 13C NMR; 298 K; 100 MHz
134 133 132 131 130 129 128 127 126 125 124 123 122 121 120 119 118 117 116 115 114
f1 (ppm)
160 150 140 130 120 110 100 90 80 70 60 50 40 30 20 10 0
f1 (ppm)

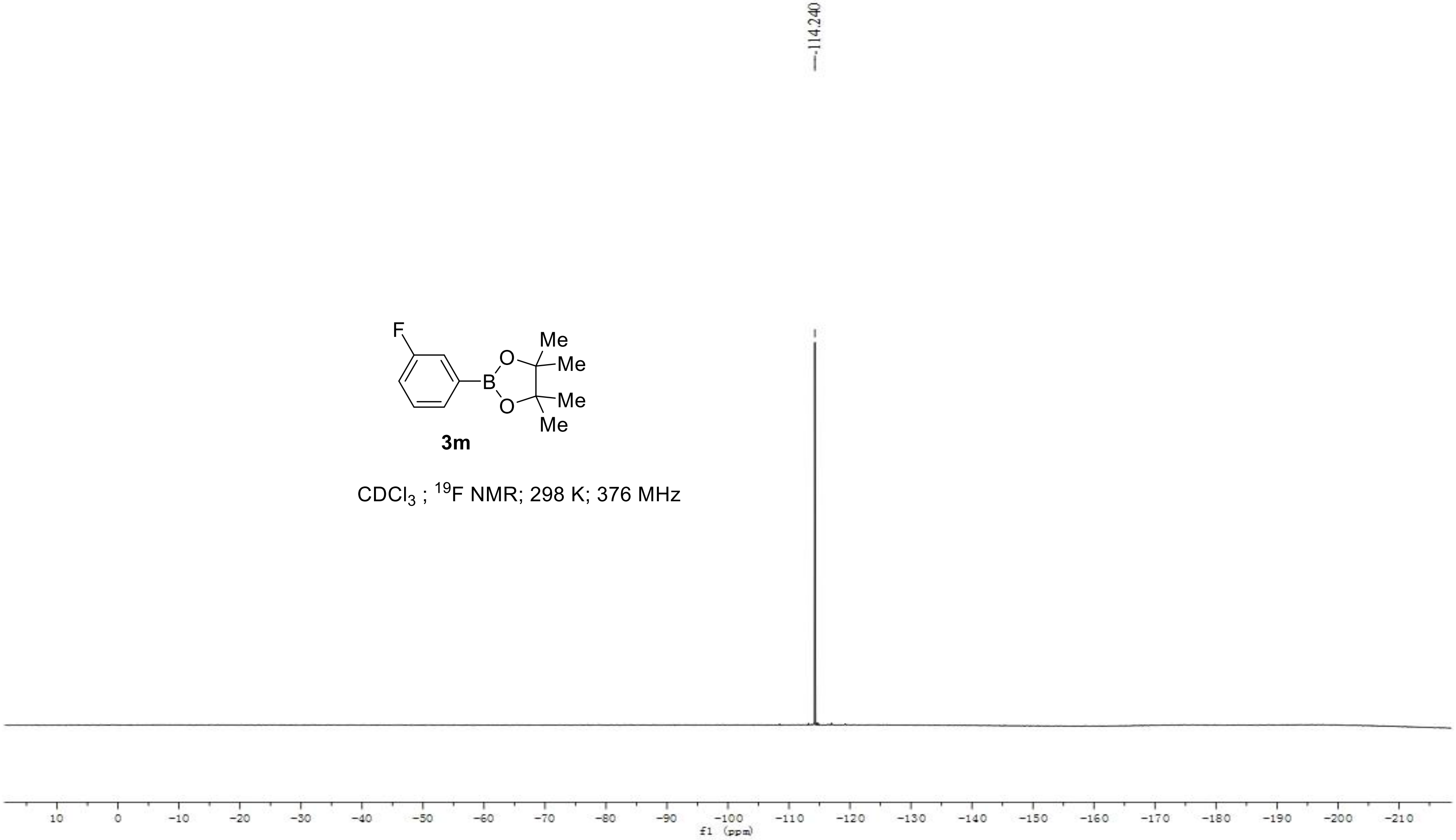
-114.240
F
Me
Me
Me
Me
O
B
O
3m
CDCl3 ; 19F NMR; 298 K; 376 MHz
10
0
−10
−20
−30
−40
−50
−60
−70
−80
−90
−100
−110
−120
−130
−140
−150
−160
−170
−180
−190
−200
−210
f1 (ppm)

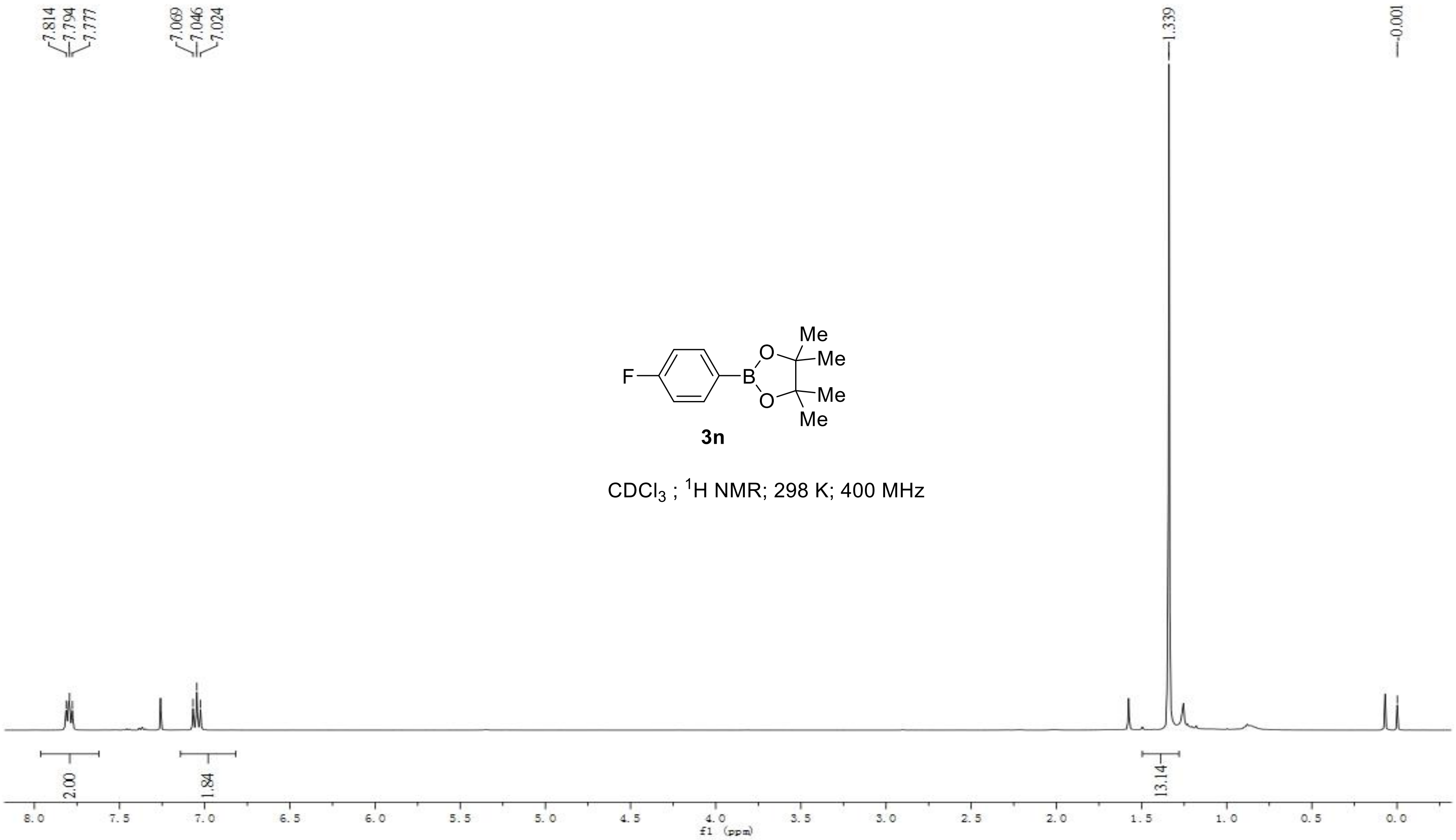

7.814
7.794
7.777
7.069
7.046
7.024
1.339
-0.001
F
B
O
O
Me
Me
Me
Me
3n
CDCl3 ; 1H NMR; 298 K; 400 MHz
2.00
1.84
13.14
8.0
7.5
7.0
6.5
6.0
5.5
5.0
4.5
4.0
3.5
3.0
2.5
2.0
1.5
1.0
0.5
0.0
f1 (ppm)

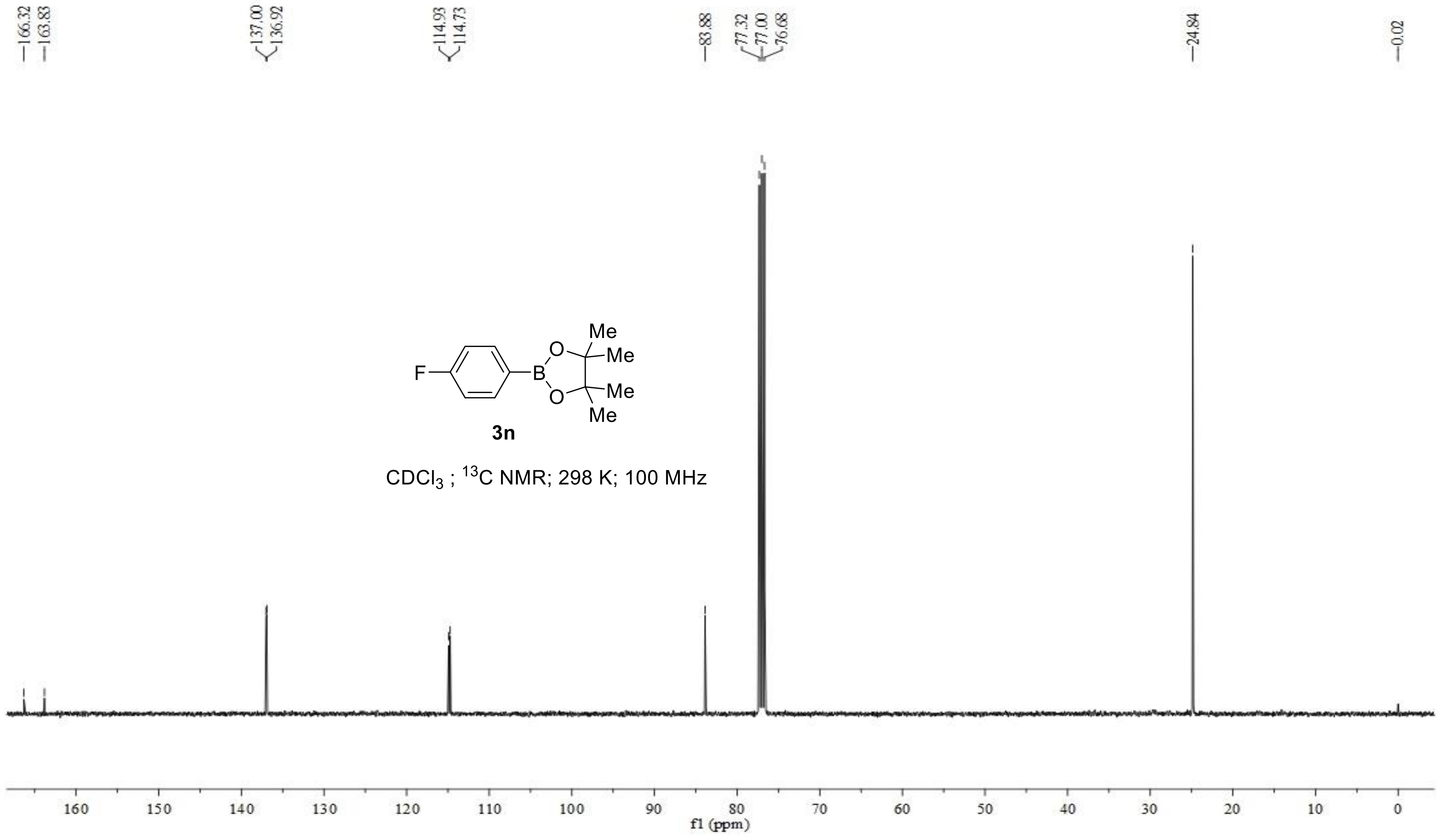
166.32
163.83
137.00
136.92
114.93
114.73
83.88
77.32
77.00
76.68
24.84
0.02
F
B
O
O
Me
Me
Me
Me
3n
CDCl3 ; 13C NMR; 298 K; 100 MHz
160
150
140
130
120
110
100
90
80
70
60
50
40
30
20
10
0
f1 (ppm)

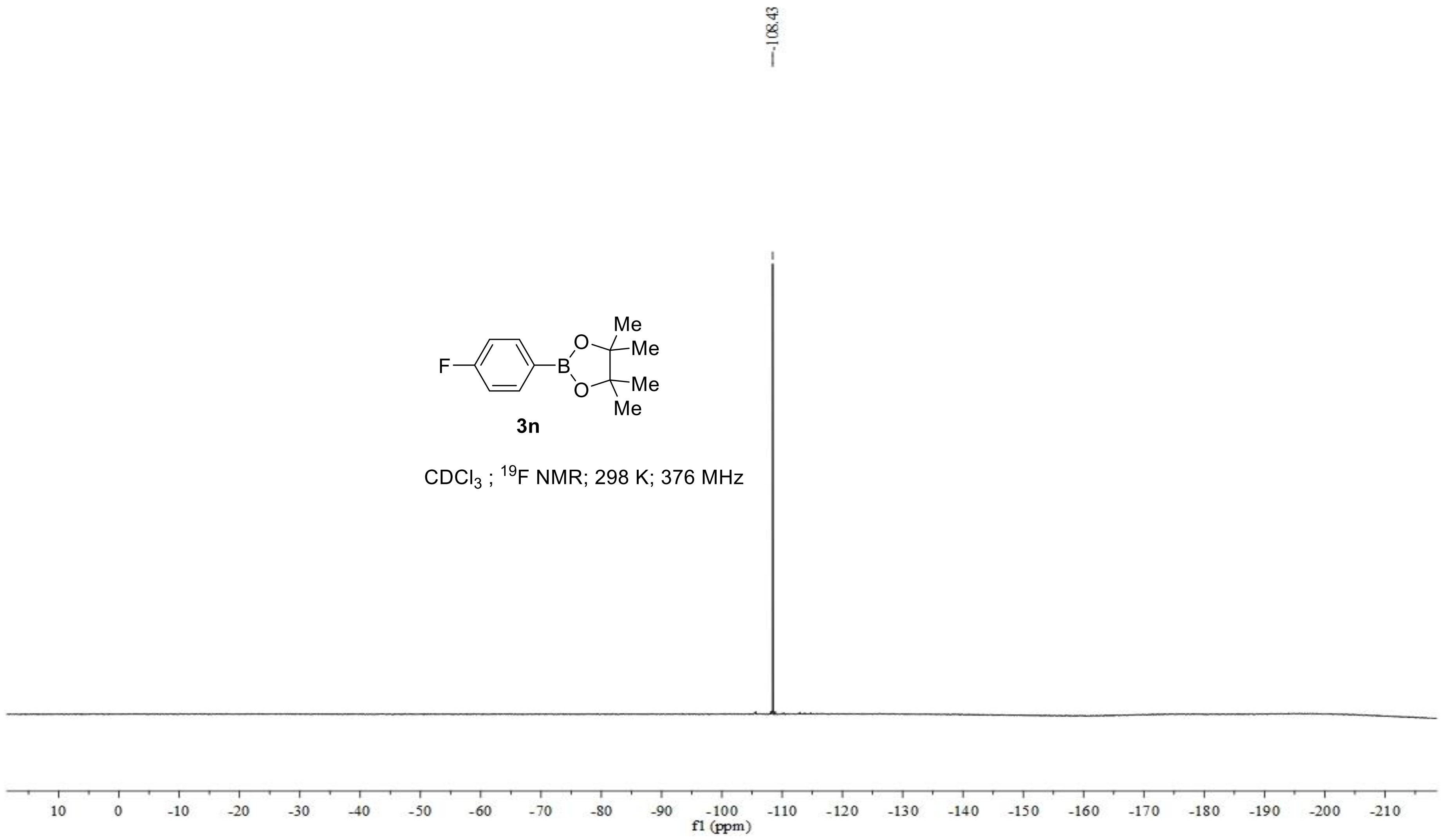

-108.43
F
B
O
O
Me
Me
Me
Me
3n
CDCl$_3$; ^{19}F NMR; 298 K; 376 MHz
10 0 -10 -20 -30 -40 -50 -60 -70 -80 -90 -100 -110 -120 -130 -140 -150 -160 -170 -180 -190 -200 -210
f1 (ppm)

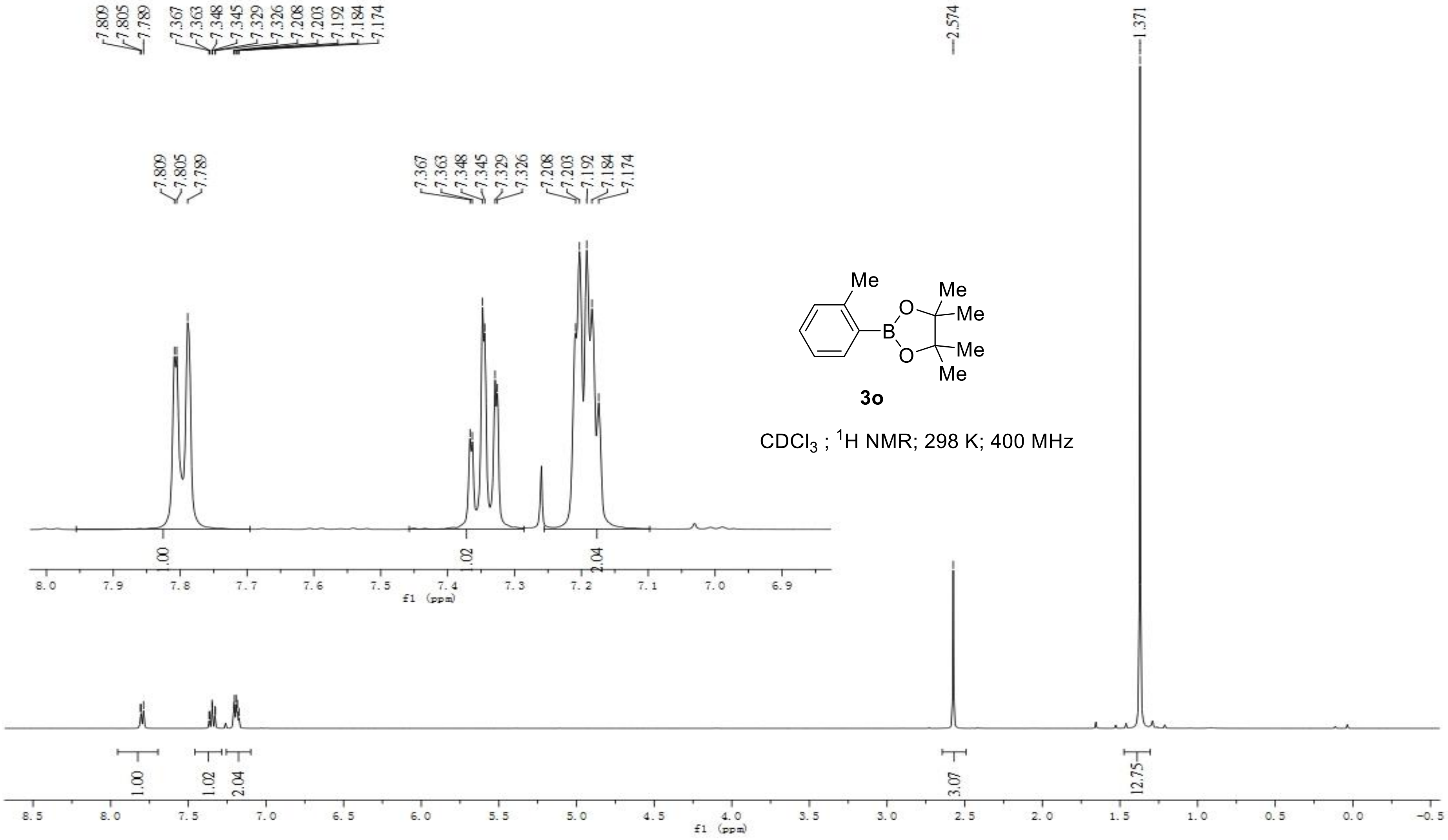

7.809
7.805
7.789
7.367
7.363
7.348
7.345
7.329
7.326
7.208
7.203
7.192
7.184
7.174
2.574
1.371
Me
O
B
O
Me
Me
Me
Me
3o
$CDCl_3$; ^{1}H NMR; 298 K; 400 MHz
1.00
1.02
2.04
3.07
12.75
f1 (ppm)

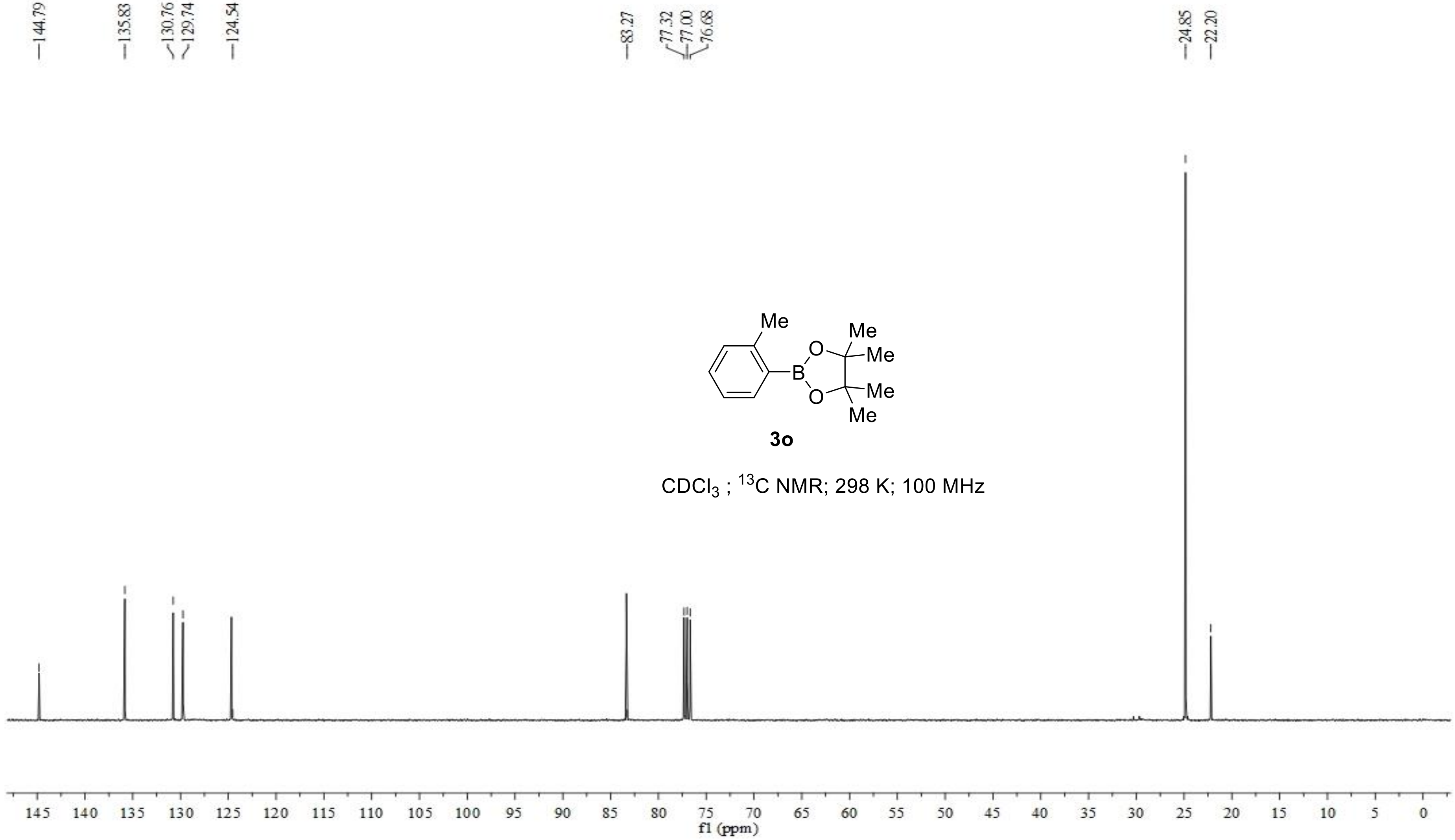

144.79
135.83
130.76
129.74
124.54
83.27
77.32
77.00
76.68
24.85
22.20
Me
Me
Me
Me
Me
O
B
O
3o
CDCl$_3$; ^{13}C NMR; 298 K; 100 MHz
145 140 135 130 125 120 115 110 105 100 95 90 85 80 75 70 65 60 55 50 45 40 35 30 25 20 15 10 5 0
f1 (ppm)

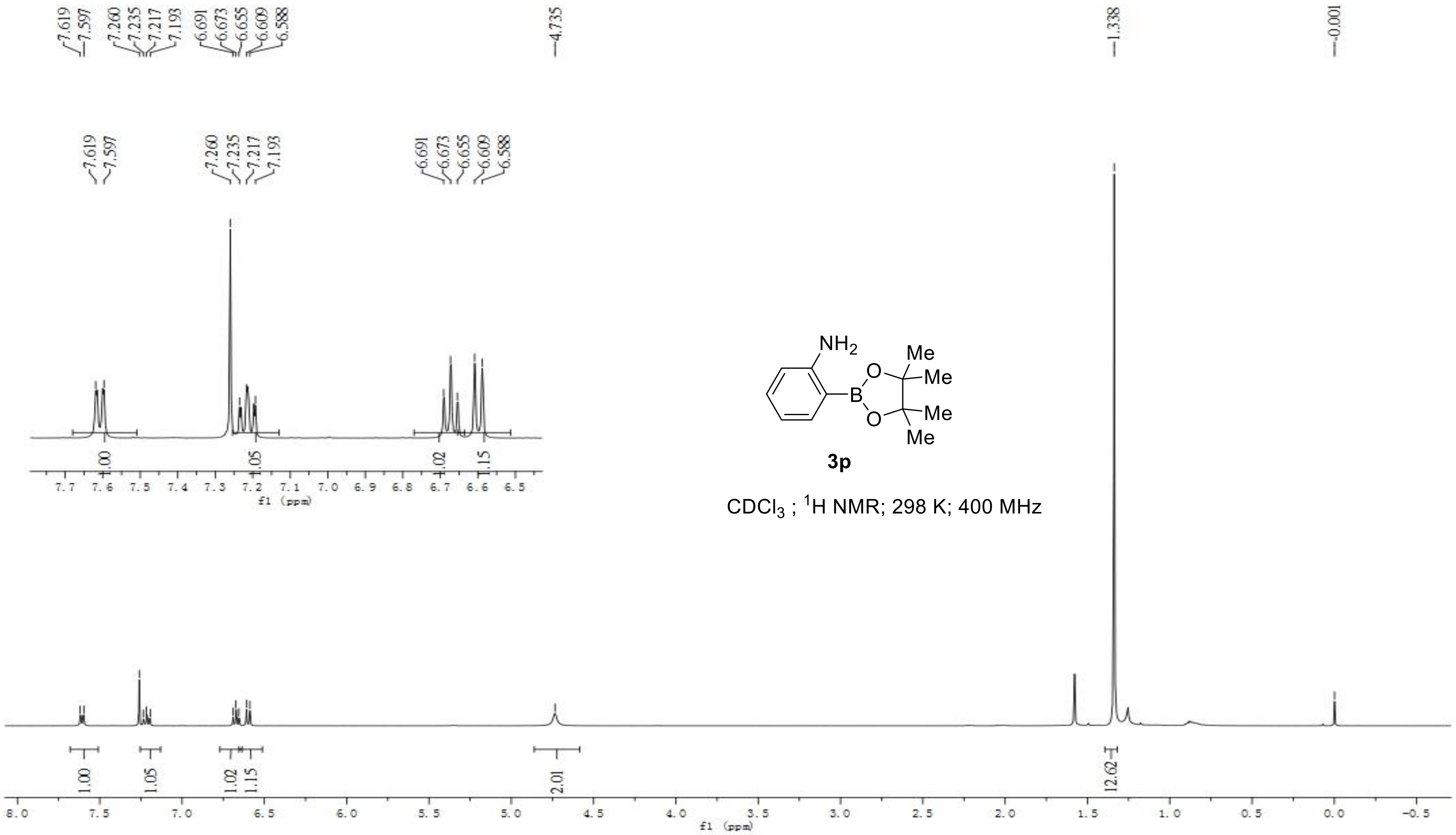
7.619
7.597
7.260
7.235
7.217
7.193
6.691
6.673
6.655
6.609
6.588
4.735
1.338
-0.001
NH2
B
O
O
Me
Me
Me
Me
3p
CDCl3 ; 1H NMR; 298 K; 400 MHz
1.00
1.05
1.02
1.15
2.01
12.62
f1 (ppm)

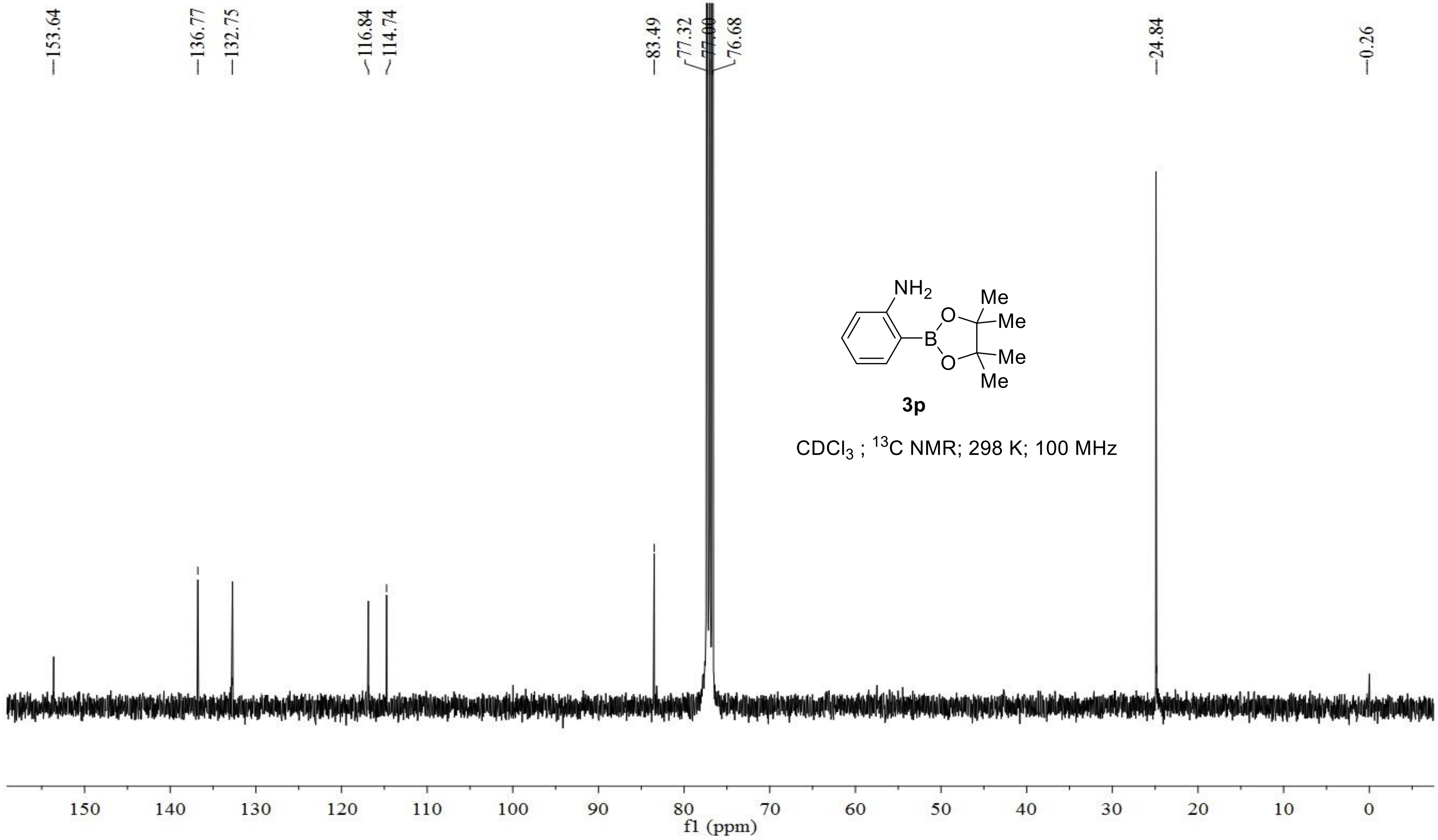
153.64
136.77
132.75
116.84
114.74
83.49
77.32
77.00
76.68
24.84
0.26
NH2
O
B
O
Me
Me
Me
Me
3p
CDCl3 ; 13C NMR; 298 K; 100 MHz
150
140
130
120
110
100
90
80
70
60
50
40
30
20
10
0
f1 (ppm)

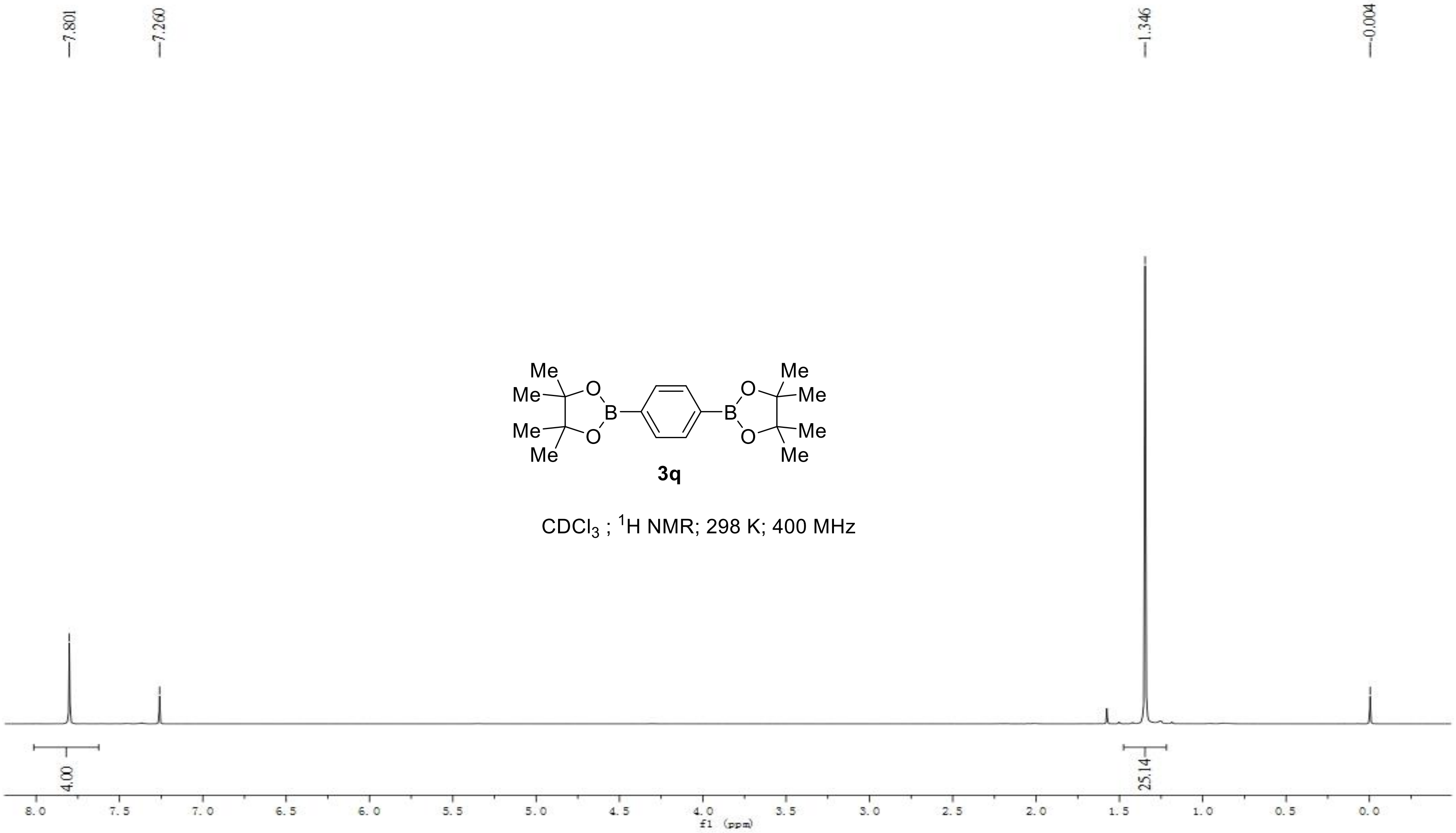
7.801
7.260
1.346
0.004
3q
CDCl$_3$; ^{1}H NMR; 298 K; 400 MHz
4.00
25.14
8.0 7.5 7.0 6.5 6.0 5.5 5.0 4.5 4.0 3.5 3.0 2.5 2.0 1.5 1.0 0.5 0.0
f1 (ppm)

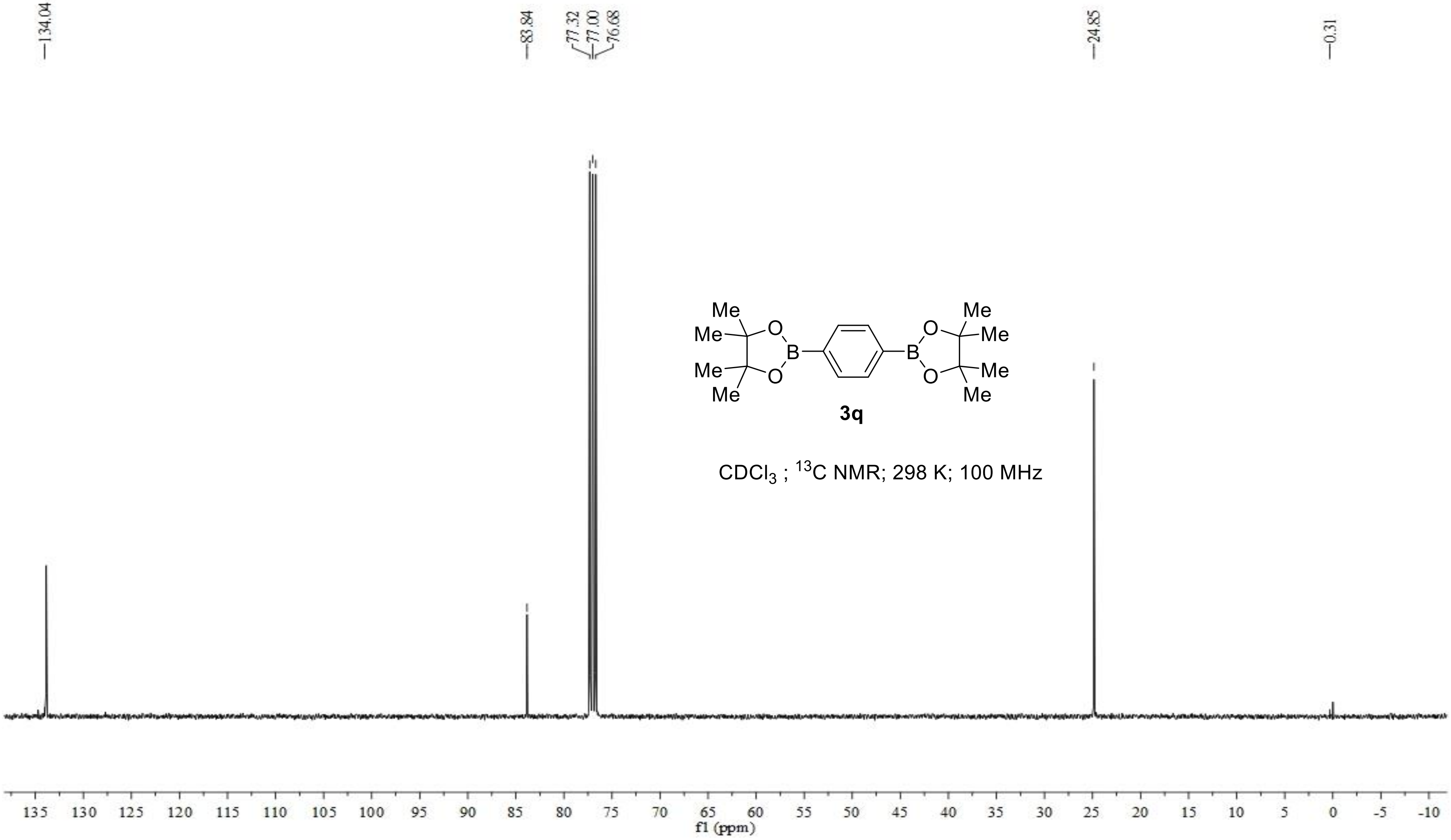

134.04
83.84
77.32
77.00
76.68
24.85
0.31
Me
Me
Me
Me
O
O
B
B
O
O
Me
Me
Me
Me
3q
CDCl3 ; 13C NMR; 298 K; 100 MHz
135 130 125 120 115 110 105 100 95 90 85 80 75 70 65 60 55 50 45 40 35 30 25 20 15 10 5 0 -5 -10
f1 (ppm)

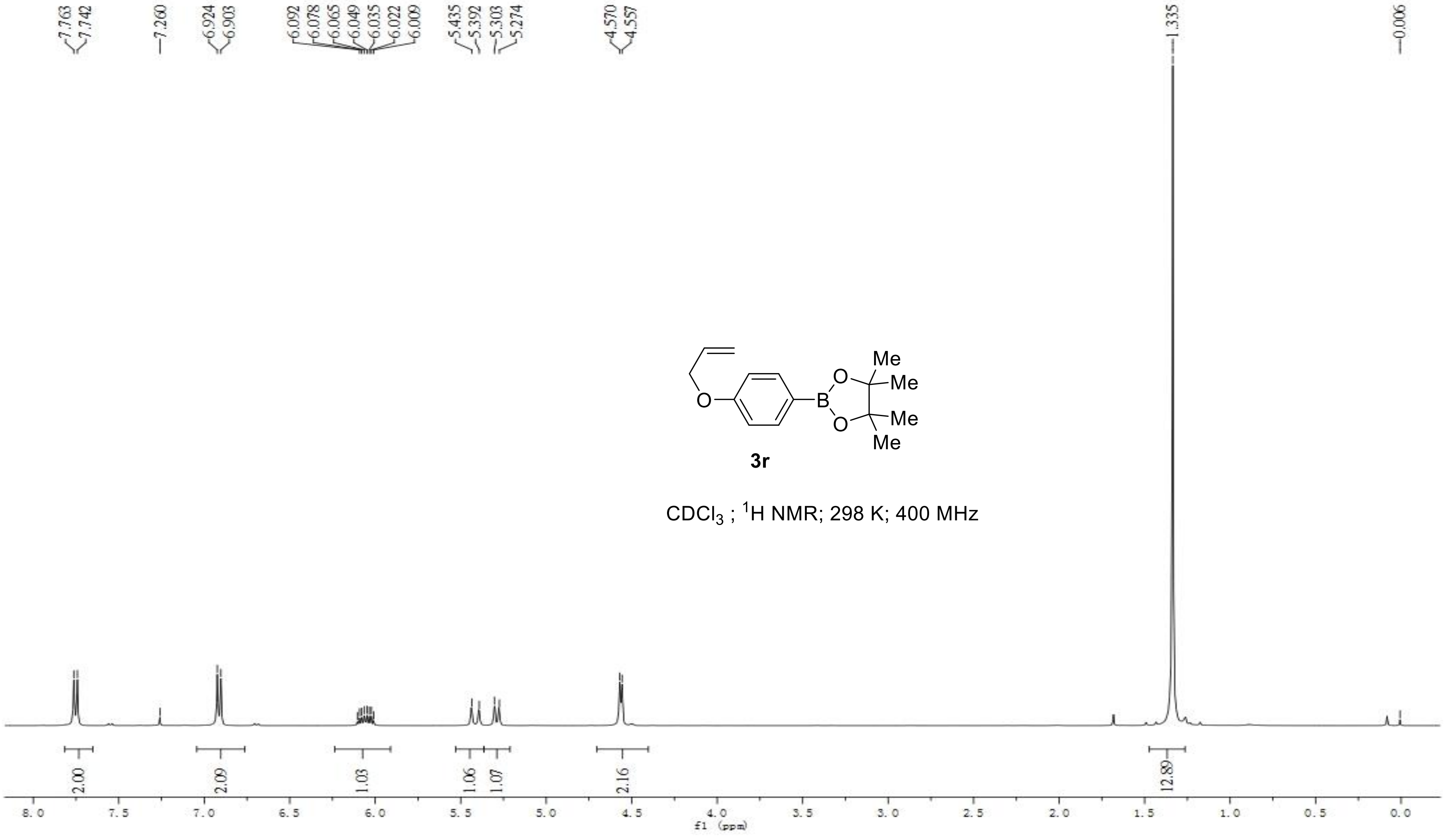
7.763
7.742
7.260
6.924
6.903
6.092
6.078
6.065
6.049
6.035
6.022
6.009
5.435
5.392
5.303
5.274
4.570
4.557
1.335
0.006
Me
Me
Me
Me
O
O
B
O
3r
CDCl3 ; 1H NMR; 298 K; 400 MHz
2.00
2.09
1.03
1.06
1.07
2.16
12.89
8.0
7.5
7.0
6.5
6.0
5.5
5.0
4.5
4.0
3.5
3.0
2.5
2.0
1.5
1.0
0.5
0.0
f1 (ppm)

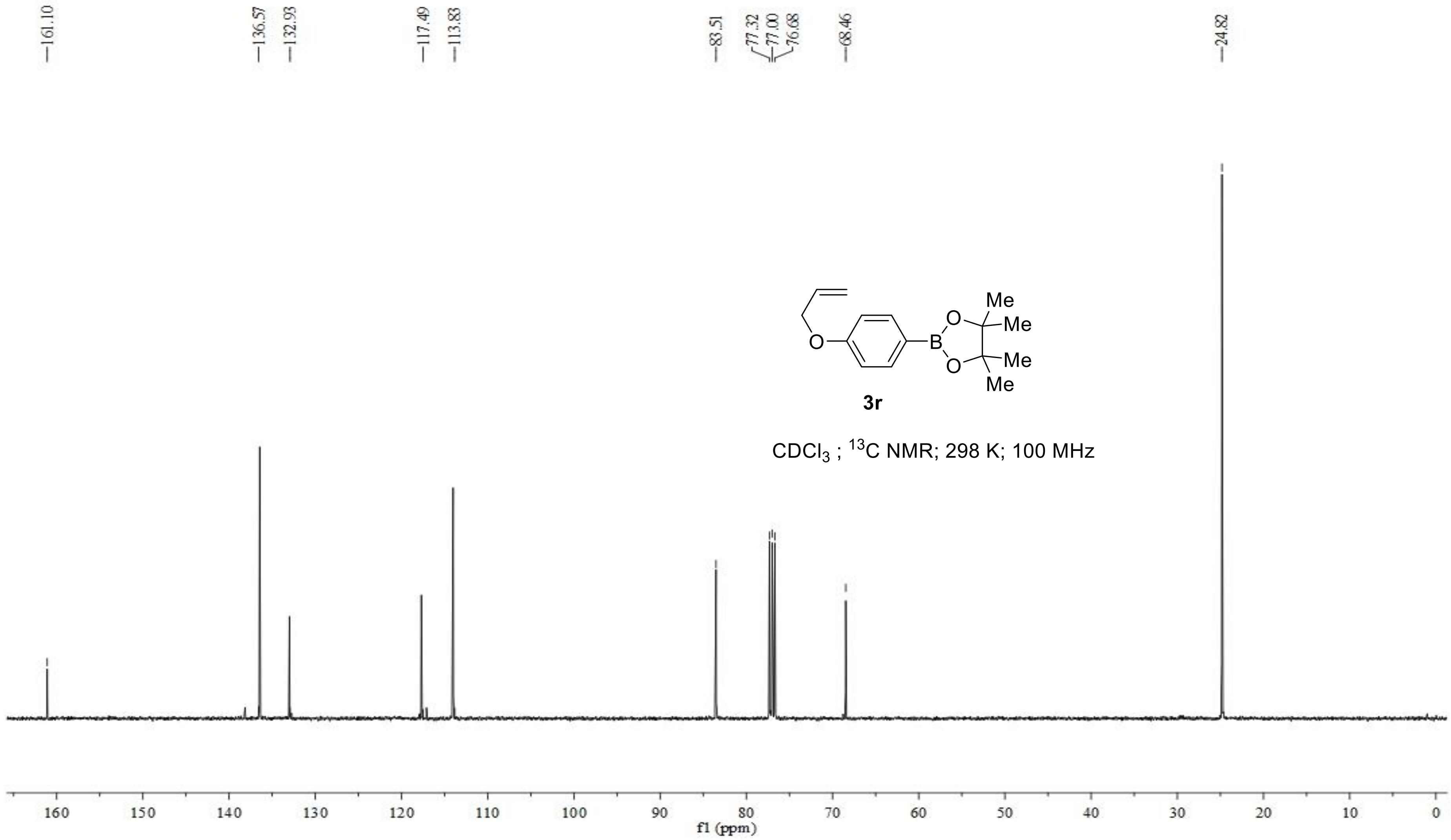
161.10
136.57
132.93
117.49
113.83
83.51
77.32
77.00
76.68
68.46
24.82
Me
O
Me
O
B
Me
O
Me
3r
CDCl3 ; 13C NMR; 298 K; 100 MHz
160
150
140
130
120
110
100
90
80
70
60
50
40
30
20
10
0
f1 (ppm)

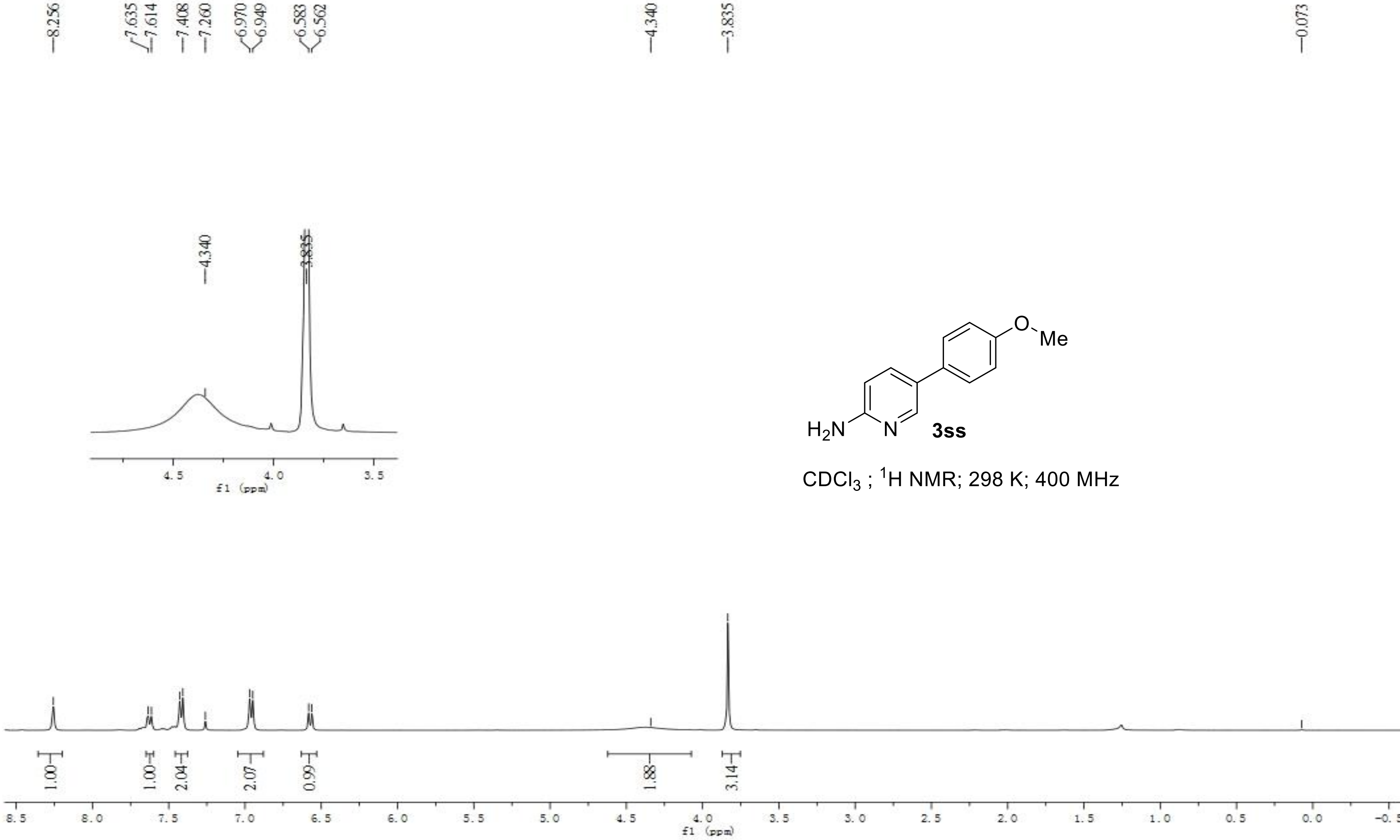

CDCl$_3$; ^{1}H NMR; 298 K; 400 MHz

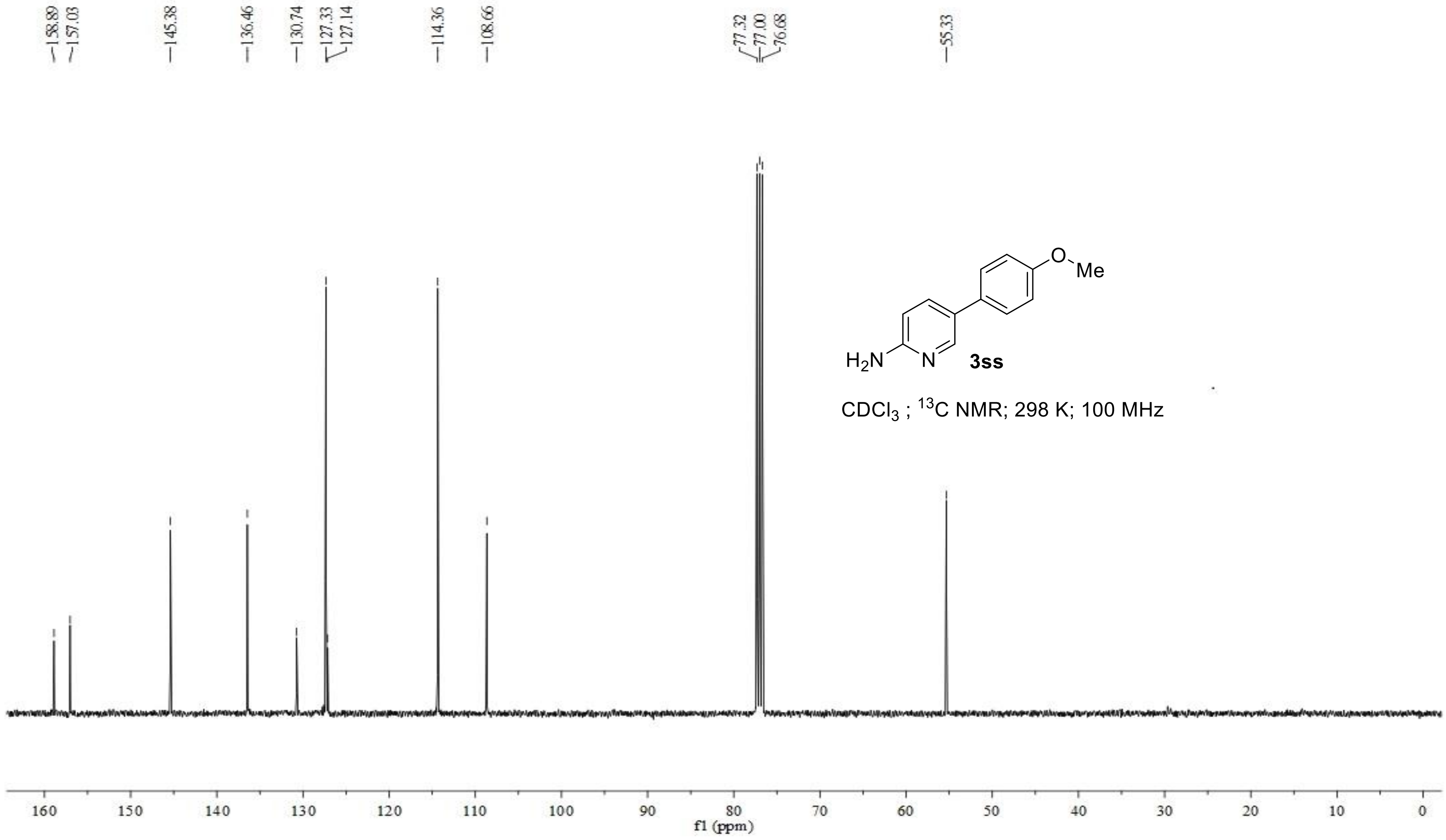
158.89
157.03
145.38
136.46
130.74
127.33
127.14
114.36
108.66
77.32
77.00
76.68
55.33
O
Me
H2N
N
3ss
CDCl3 ; 13C NMR; 298 K; 100 MHz
160 150 140 130 120 110 100 90 80 70 60 50 40 30 20 10 0
f1 (ppm)

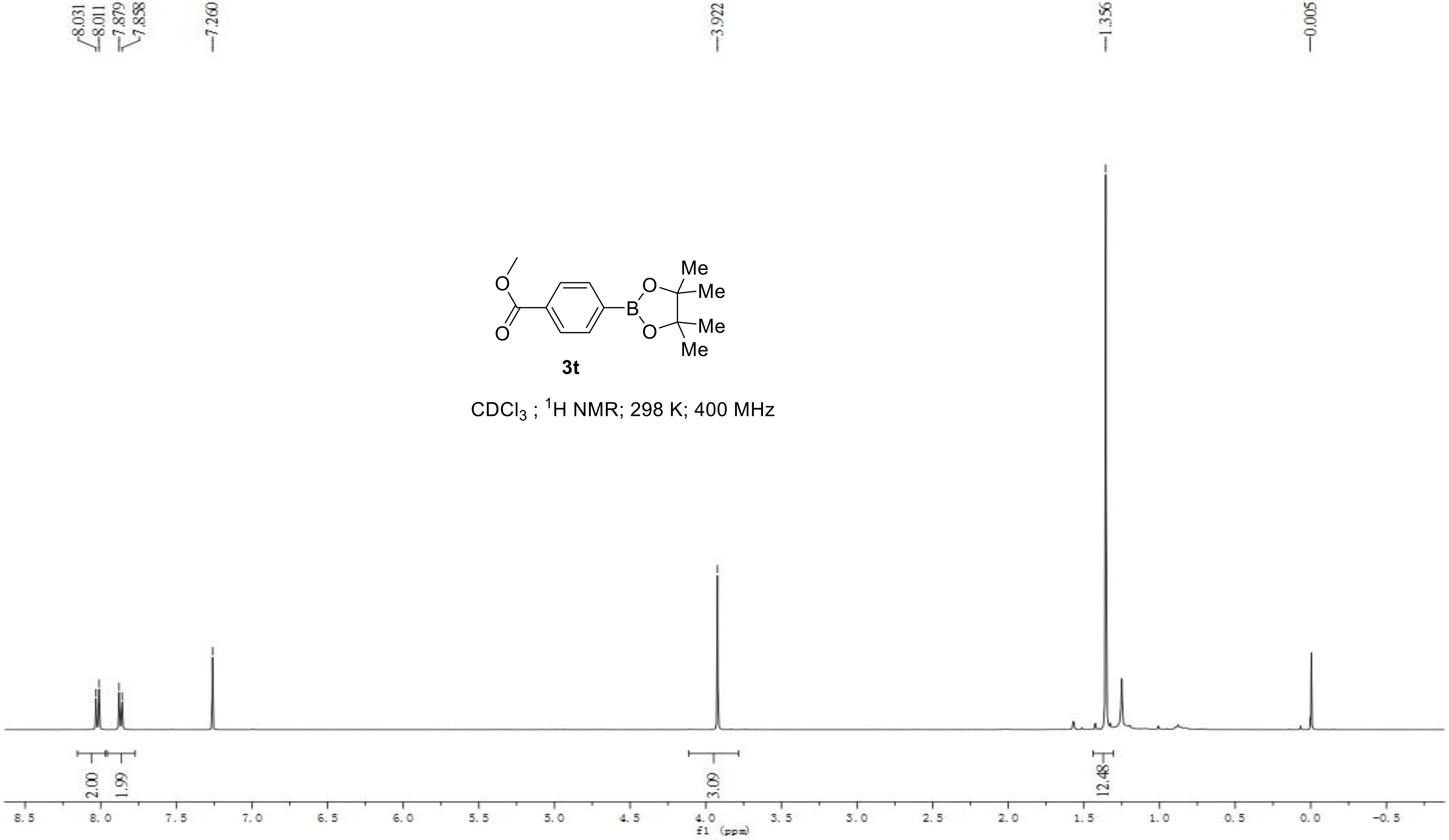

8.031
8.011
7.879
7.858
7.260
3.922
1.356
0.005
Me
Me
Me
Me
O
O
B
O
O
3t
CDCl3 ; 1H NMR; 298 K; 400 MHz
2.00
1.99
3.09
12.48
8.5 8.0 7.5 7.0 6.5 6.0 5.5 5.0 4.5 4.0 3.5 3.0 2.5 2.0 1.5 1.0 0.5 0.0 -0.5
f1 (ppm)

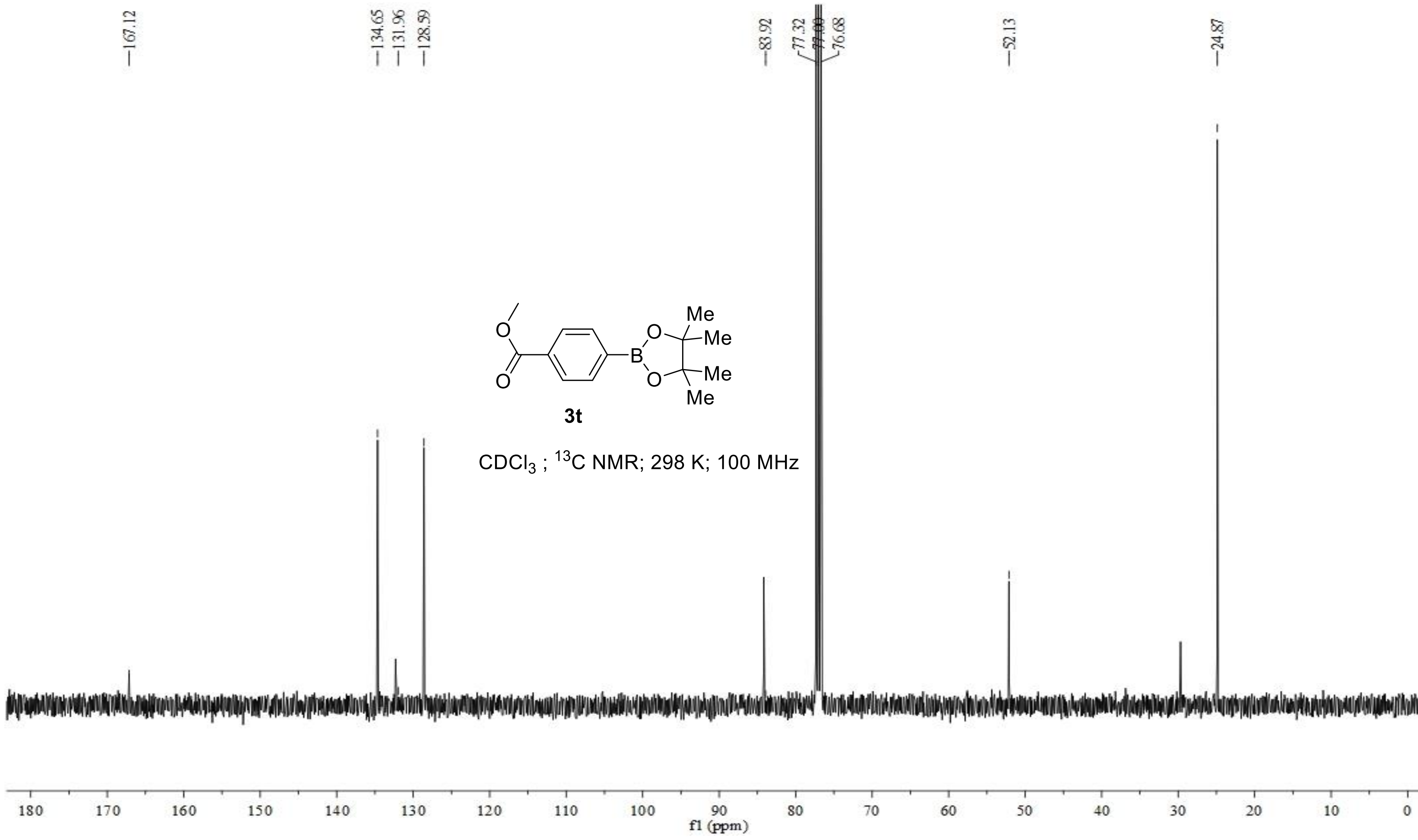

167.12
134.65
131.96
128.59
83.92
77.32
77.00
76.68
52.13
24.87
O
O
B
O
O
Me
Me
Me
Me
3t
$CDCl_3$; ^{13}C NMR; 298 K; 100 MHz
180 170 160 150 140 130 120 110 100 90 80 70 60 50 40 30 20 10 0
f1 (ppm)

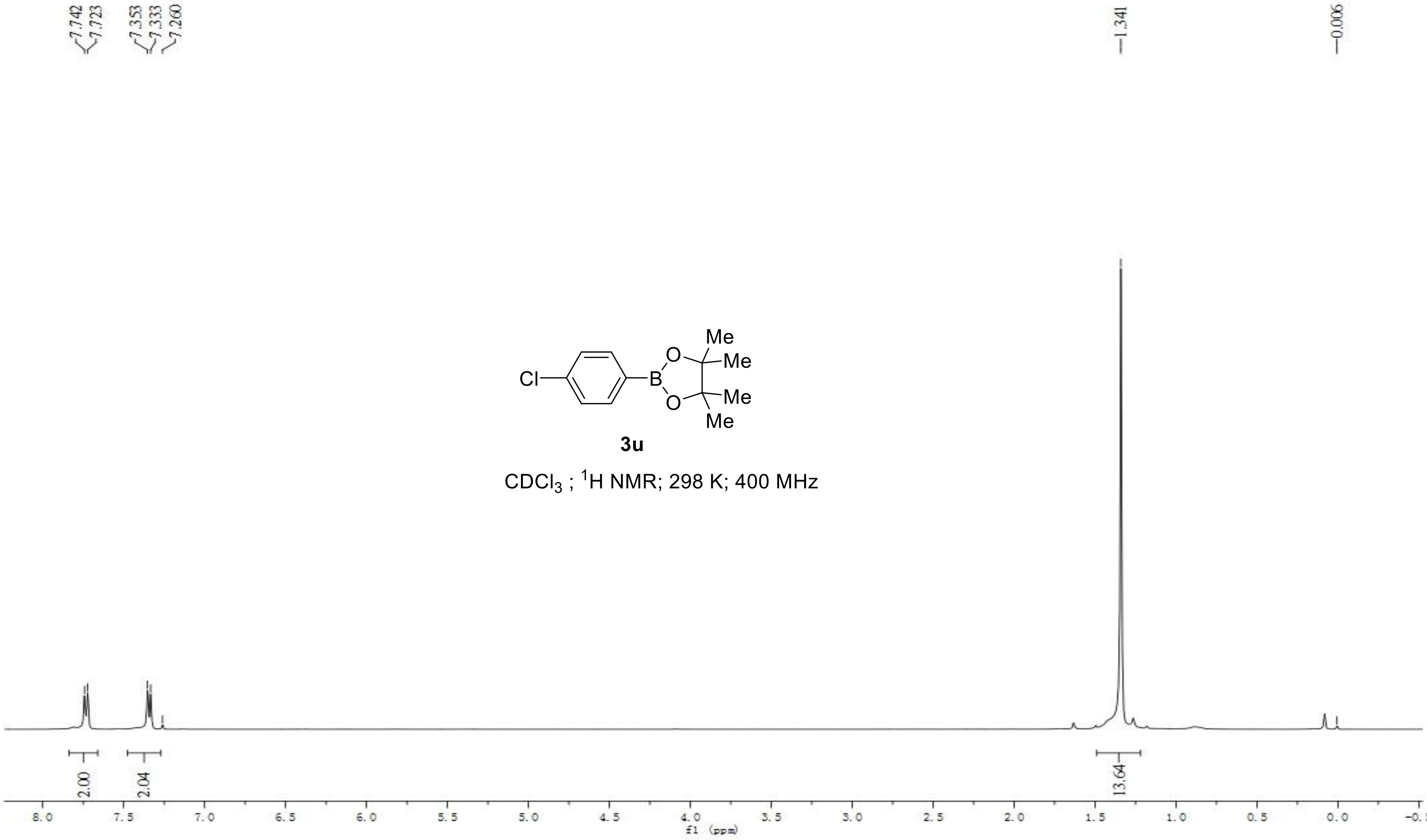
7.742
7.723
7.353
7.333
7.260
1.341
0.006
Me
Me
Me
Me
Cl
B
O
O
3u
CDCl3 ; 1H NMR; 298 K; 400 MHz
2.00
2.04
13.64
8.0
7.5
7.0
6.5
6.0
5.5
5.0
4.5
4.0
3.5
3.0
2.5
2.0
1.5
1.0
0.5
0.0
-0.5
f1 (ppm)

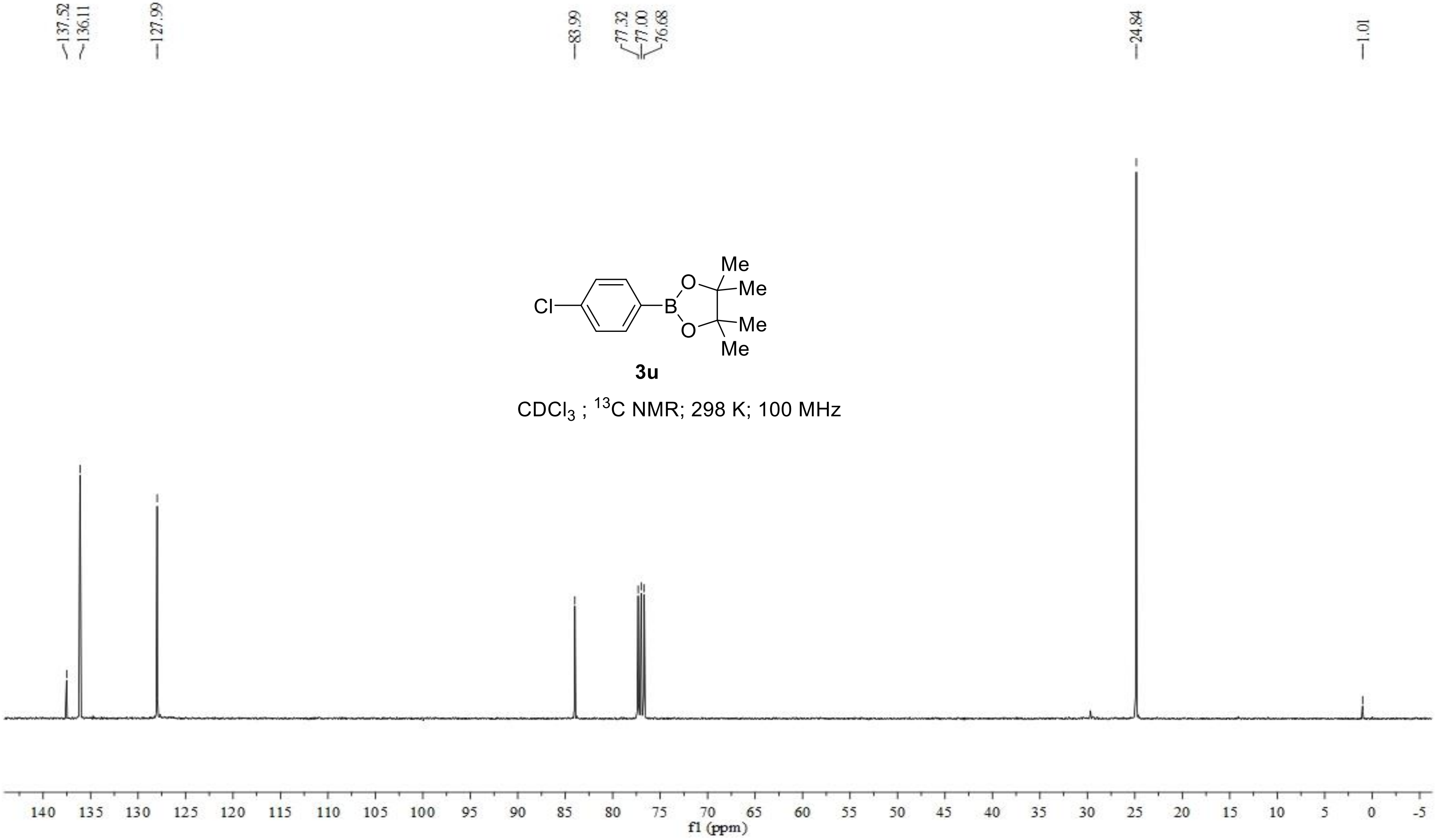

3u

$CDCl_3$; ^{13}C NMR; 298 K; 100 MHz

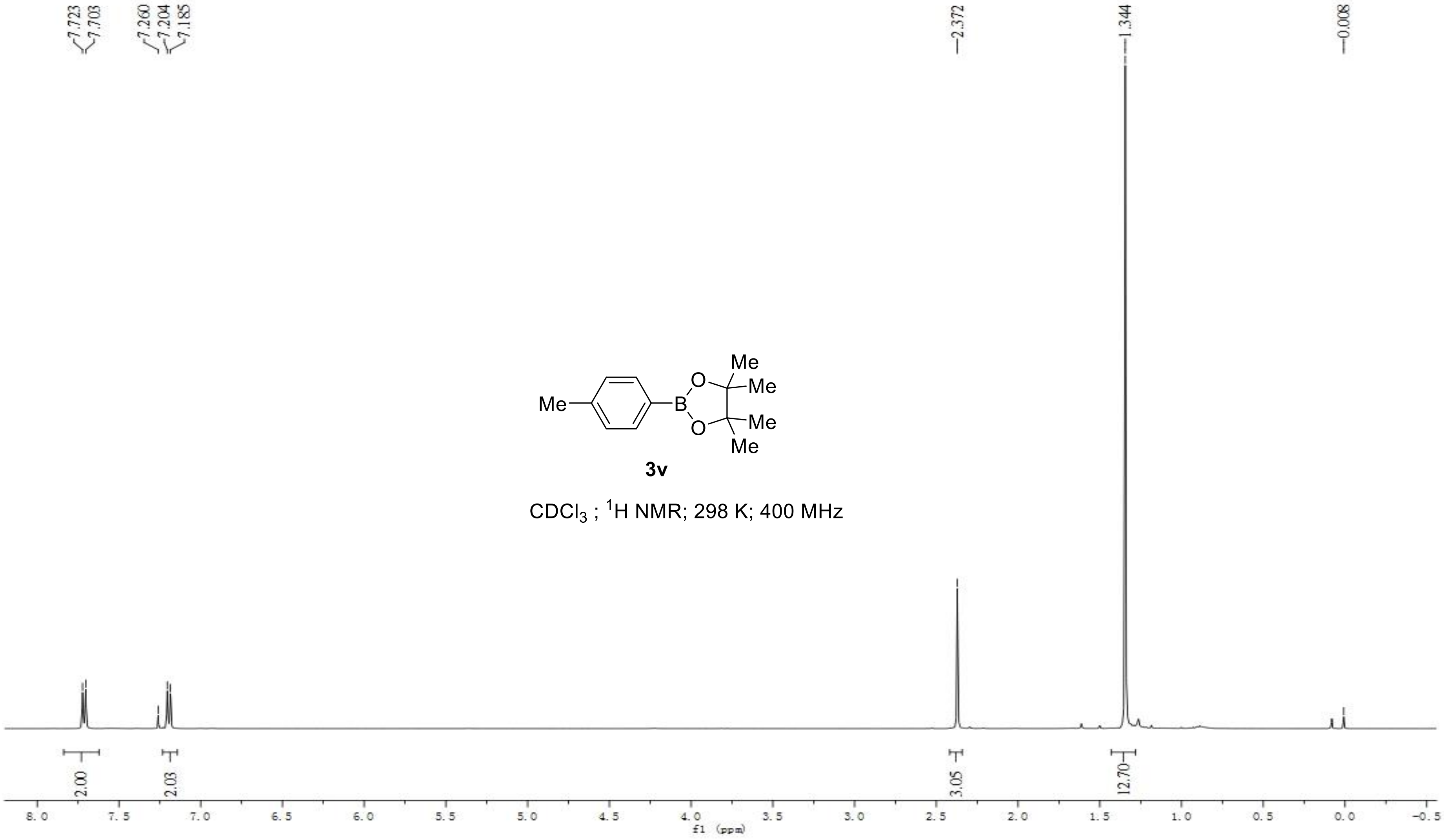
7.723
7.703
7.260
7.204
7.185
2.372
1.344
-0.008
Me
3v
CDCl$_3$; ^{1}H NMR; 298 K; 400 MHz
2.00
2.03
3.05
12.70
8.0 7.5 7.0 6.5 6.0 5.5 5.0 4.5 4.0 3.5 3.0 2.5 2.0 1.5 1.0 0.5 0.0 -0.5
f1 (ppm)

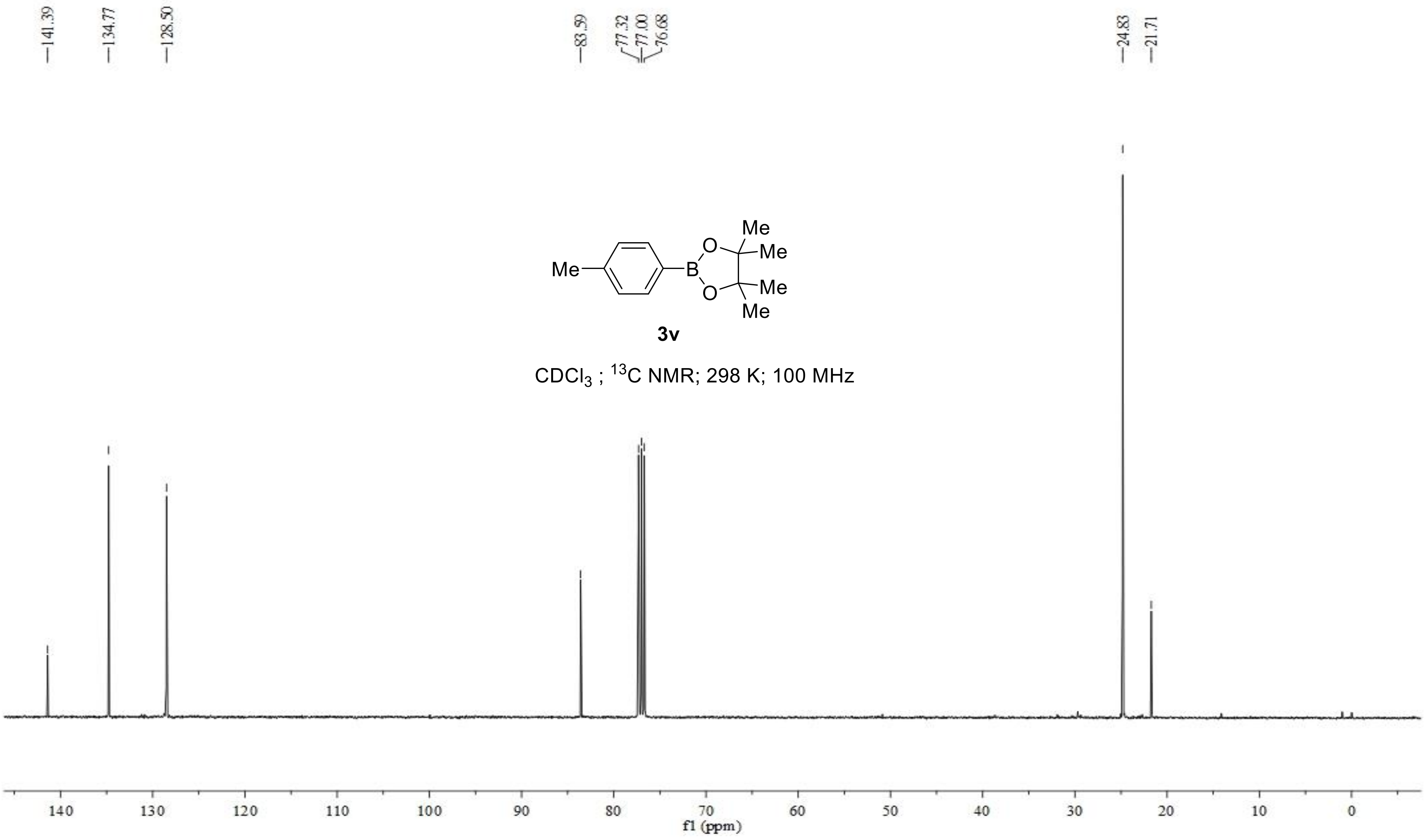

3v

$CDCl_3$; ^{13}C NMR; 298 K; 100 MHz

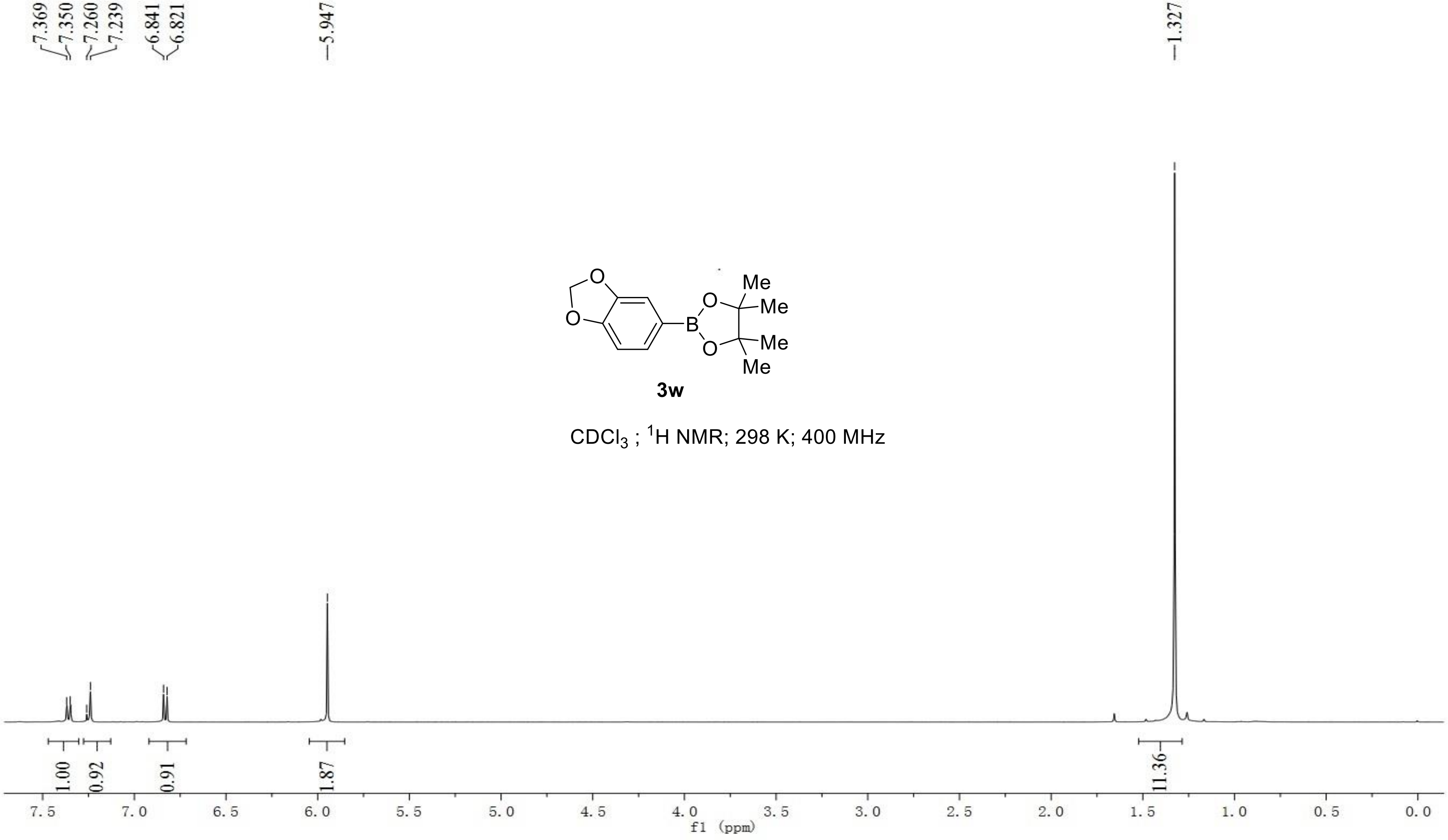
7.369
7.350
7.260
7.239
6.841
6.821
5.947
1.327
Me
Me
Me
Me
O
O
O
O
B
3w
CDCl3 ; 1H NMR; 298 K; 400 MHz
1.00
0.92
0.91
1.87
11.36
7.5
7.0
6.5
6.0
5.5
5.0
4.5
4.0
3.5
3.0
2.5
2.0
1.5
1.0
0.5
0.0
f1 (ppm)

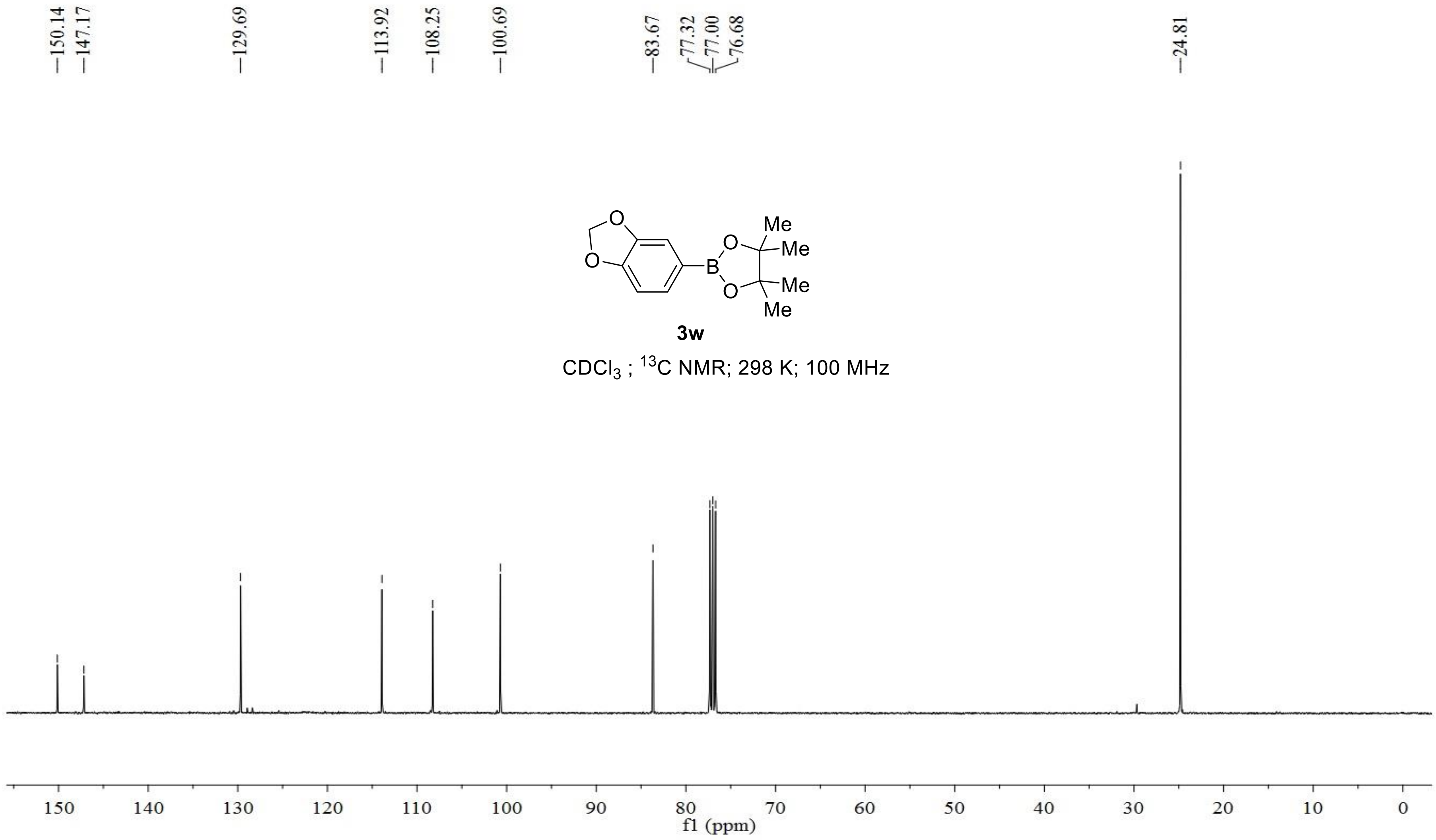

150.14
147.17
129.69
113.92
108.25
100.69
83.67
77.32
77.00
76.68
24.81
Me
Me
Me
Me
O
O
B
O
O
3w
CDCl3 ; 13C NMR; 298 K; 100 MHz
150 140 130 120 110 100 90 80 70 60 50 40 30 20 10 0
f1 (ppm)

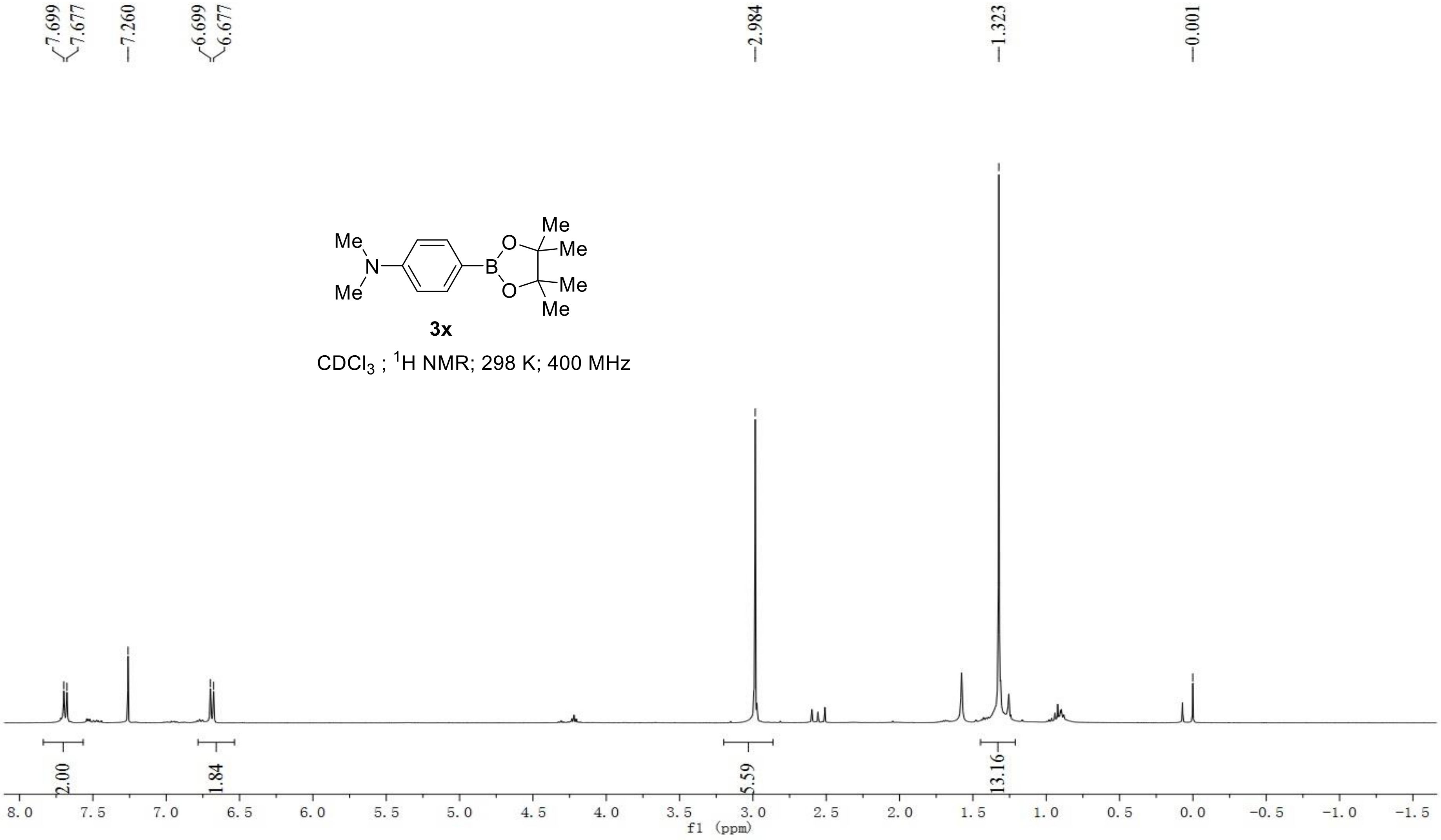
7.699
7.677
7.260
6.699
6.677
2.984
1.323
-0.001
Me
N
Me
B
O
O
Me
Me
Me
Me
3x
$CDCl_3$; ^{1}H NMR; 298 K; 400 MHz
2.00
1.84
5.59
13.16
8.0 7.5 7.0 6.5 6.0 5.5 5.0 4.5 4.0 3.5 3.0 2.5 2.0 1.5 1.0 0.5 0.0 -0.5 -1.0 -1.5
f1 (ppm)

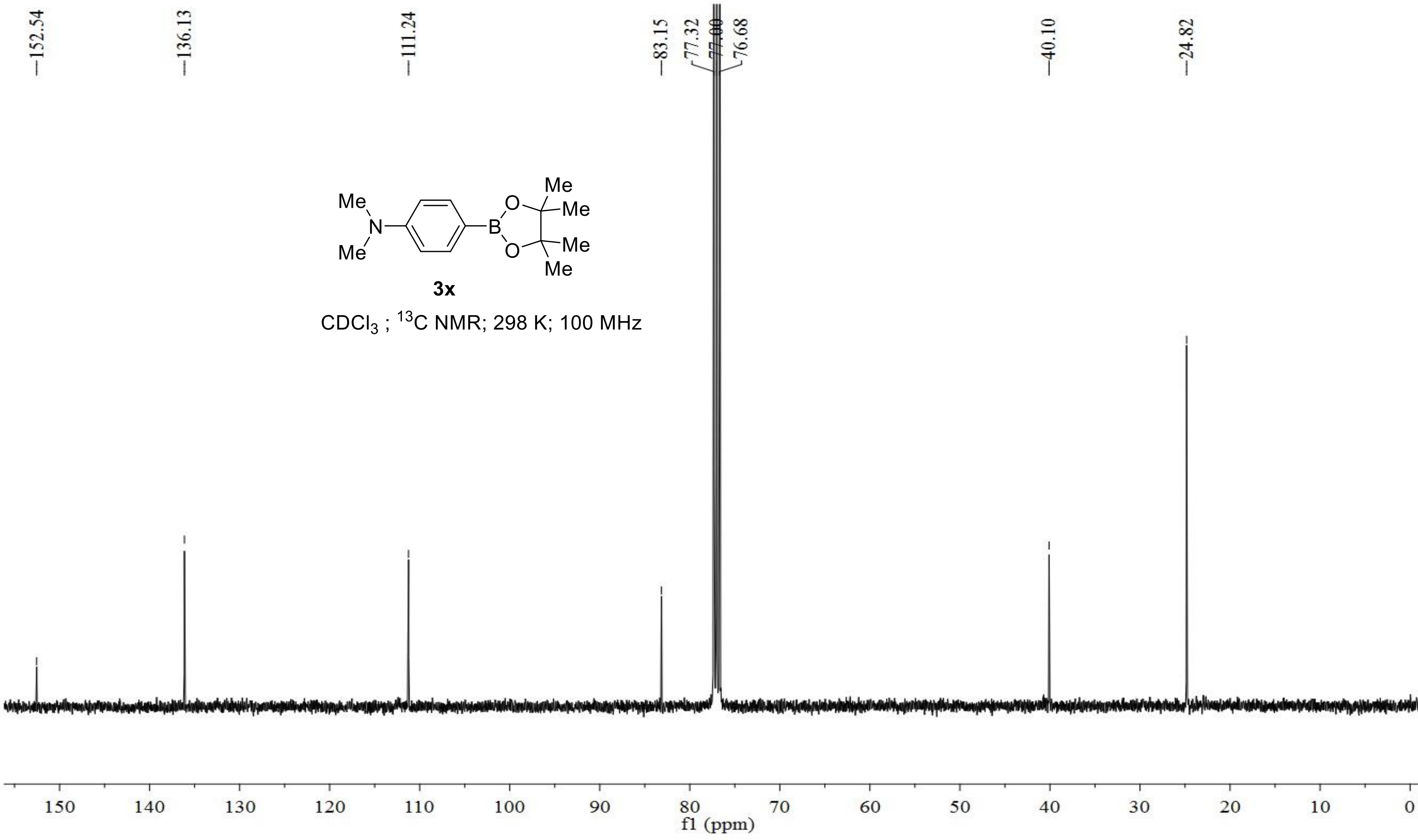
152.54
136.13
111.24
83.15
77.32
77.00
76.68
40.10
24.82
Me
N
Me
B
O
O
Me
Me
Me
Me
3x
CDCl3 ; 13C NMR; 298 K; 100 MHz
150
140
130
120
110
100
90
80
70
60
50
40
30
20
10
0
f1 (ppm)

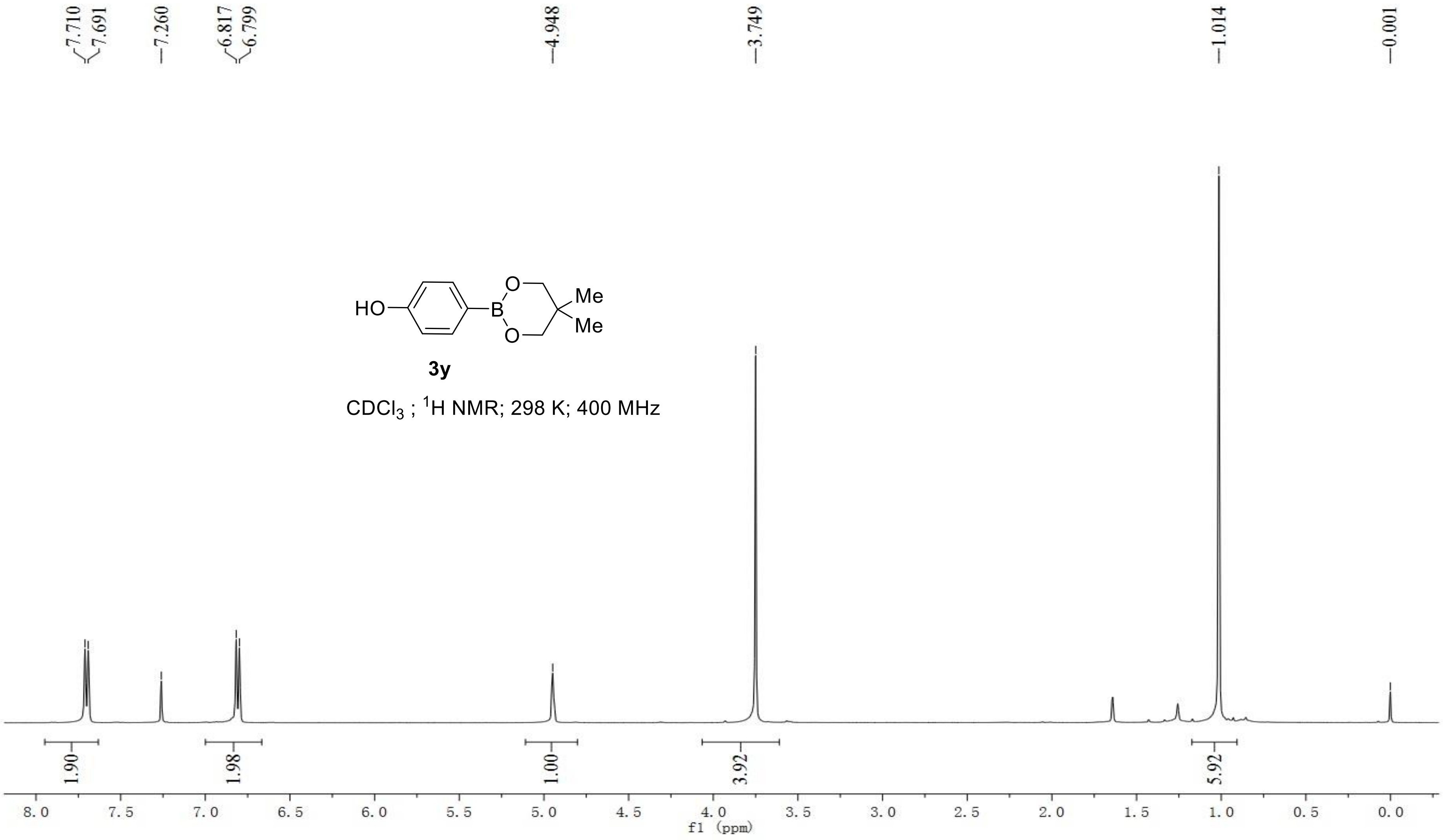

7.710
7.691
7.260
6.817
6.799
4.948
3.749
1.014
0.001
HO
B
O
O
Me
Me
3y
CDCl$_3$; ^{1}H NMR; 298 K; 400 MHz
1.90
1.98
1.00
3.92
5.92
8.0 7.5 7.0 6.5 6.0 5.5 5.0 4.5 4.0 3.5 3.0 2.5 2.0 1.5 1.0 0.5 0.0
f1 (ppm)

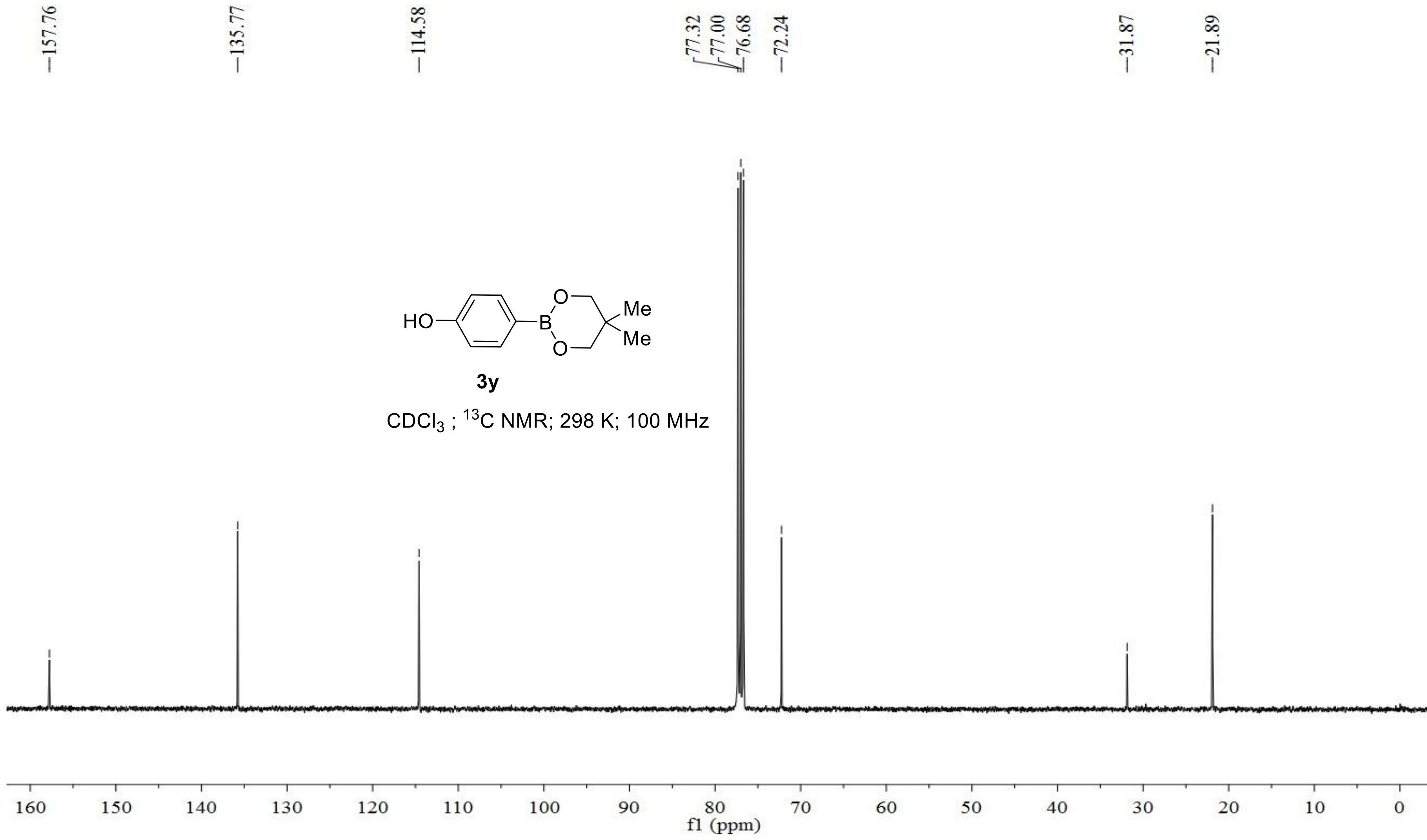
157.76
135.77
114.58
77.32
77.00
76.68
72.24
31.87
21.89
HO
B
O
O
Me
Me
3y
CDCl3 ; 13C NMR; 298 K; 100 MHz
160
150
140
130
120
110
100
90
80
70
60
50
40
30
20
10
0
f1 (ppm)

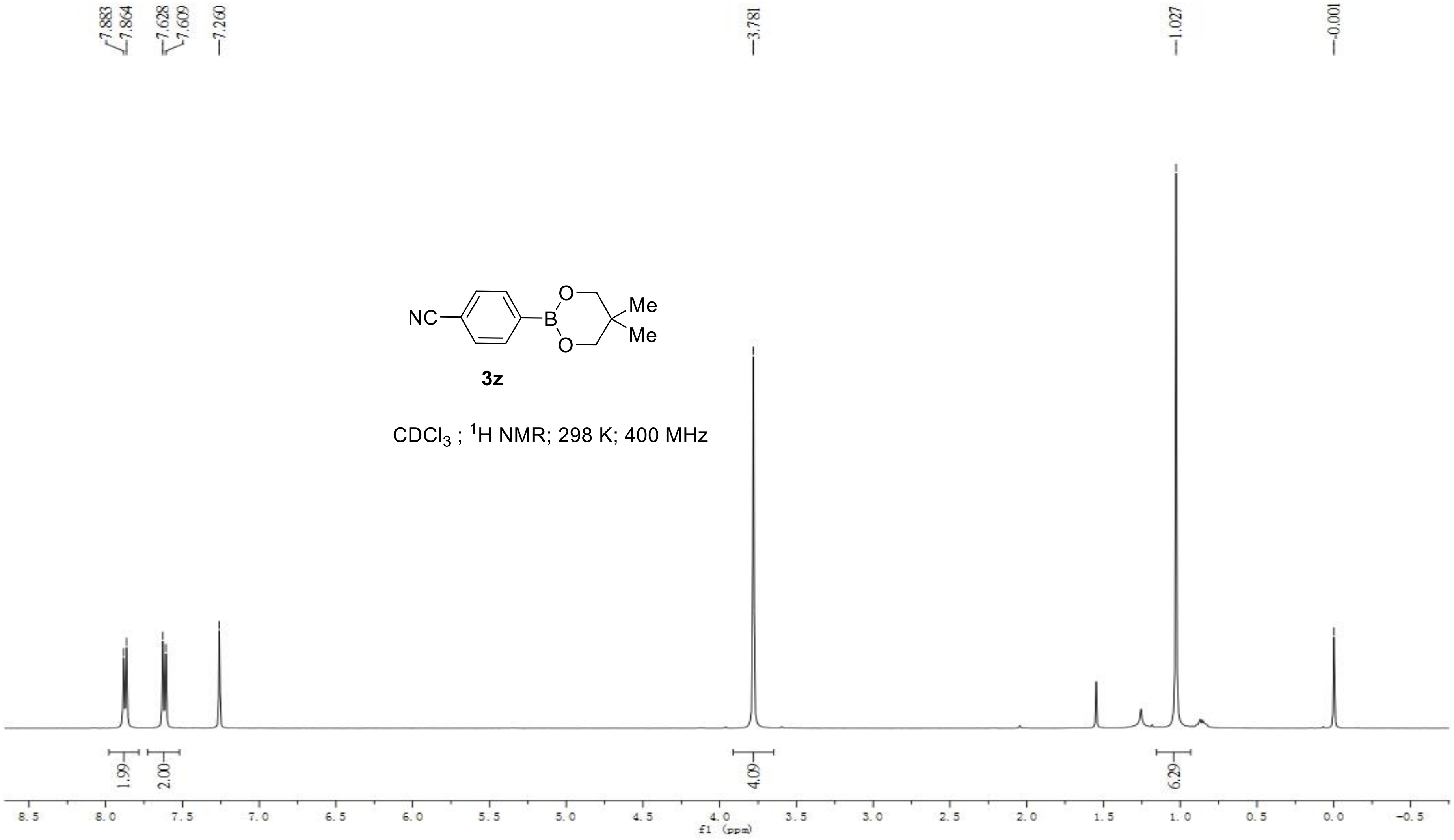
NC
B
O
O
Me
Me
3z
CDCl3 ; 1H NMR; 298 K; 400 MHz
7.883
7.864
7.628
7.609
7.260
3.781
1.027
-0.001
1.99
2.00
4.09
6.29
8.5
8.0
7.5
7.0
6.5
6.0
5.5
5.0
4.5
4.0
3.5
3.0
2.5
2.0
1.5
1.0
0.5
0.0
-0.5
f1 (ppm)

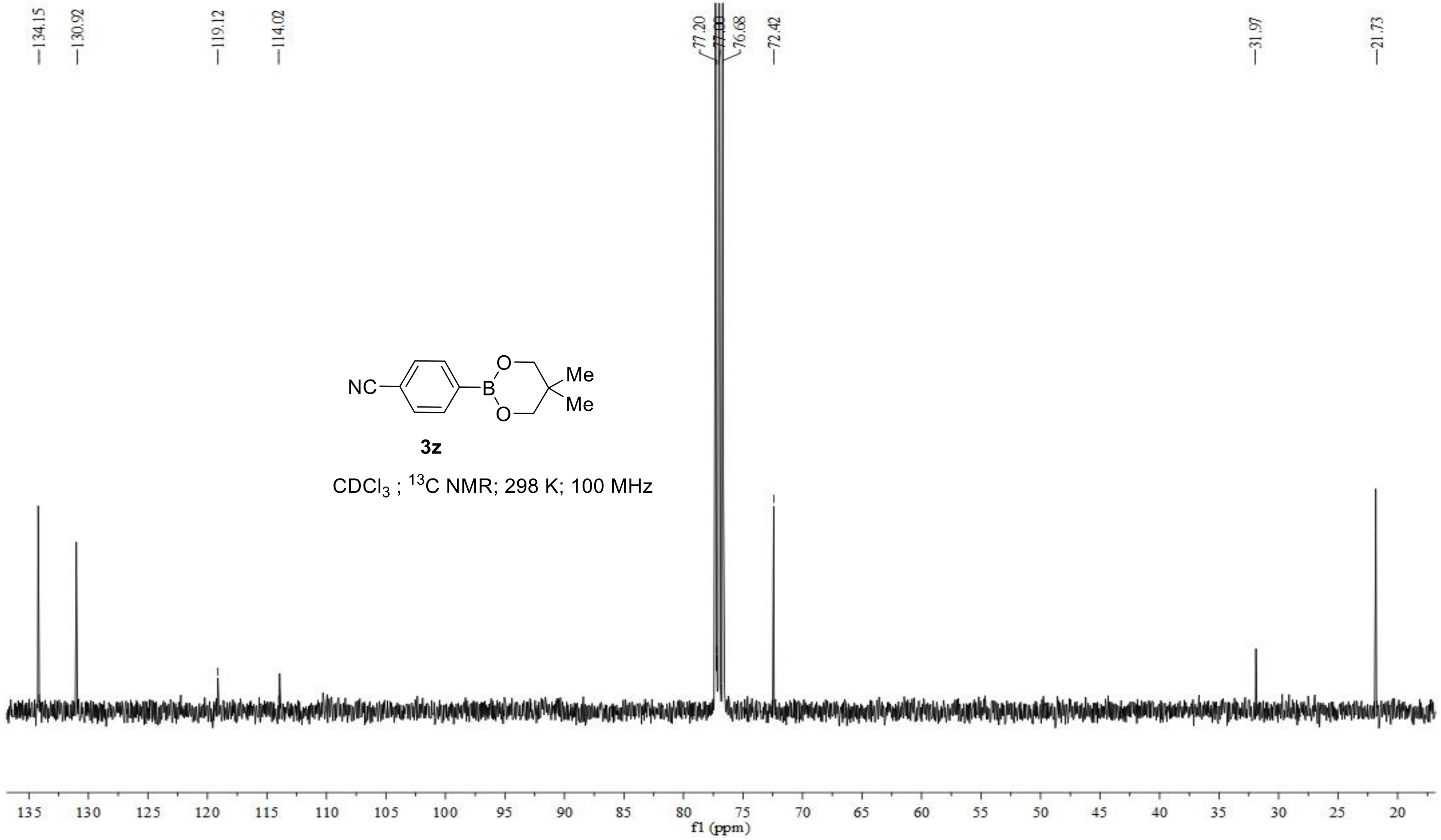
134.15
130.92
119.12
114.02
77.20
77.00
76.68
72.42
31.97
21.73
NC
B
O
O
Me
Me
3z
CDCl3 ; 13C NMR; 298 K; 100 MHz
135 130 125 120 115 110 105 100 95 90 85 80 75 70 65 60 55 50 45 40 35 30 25 20
f1 (ppm)

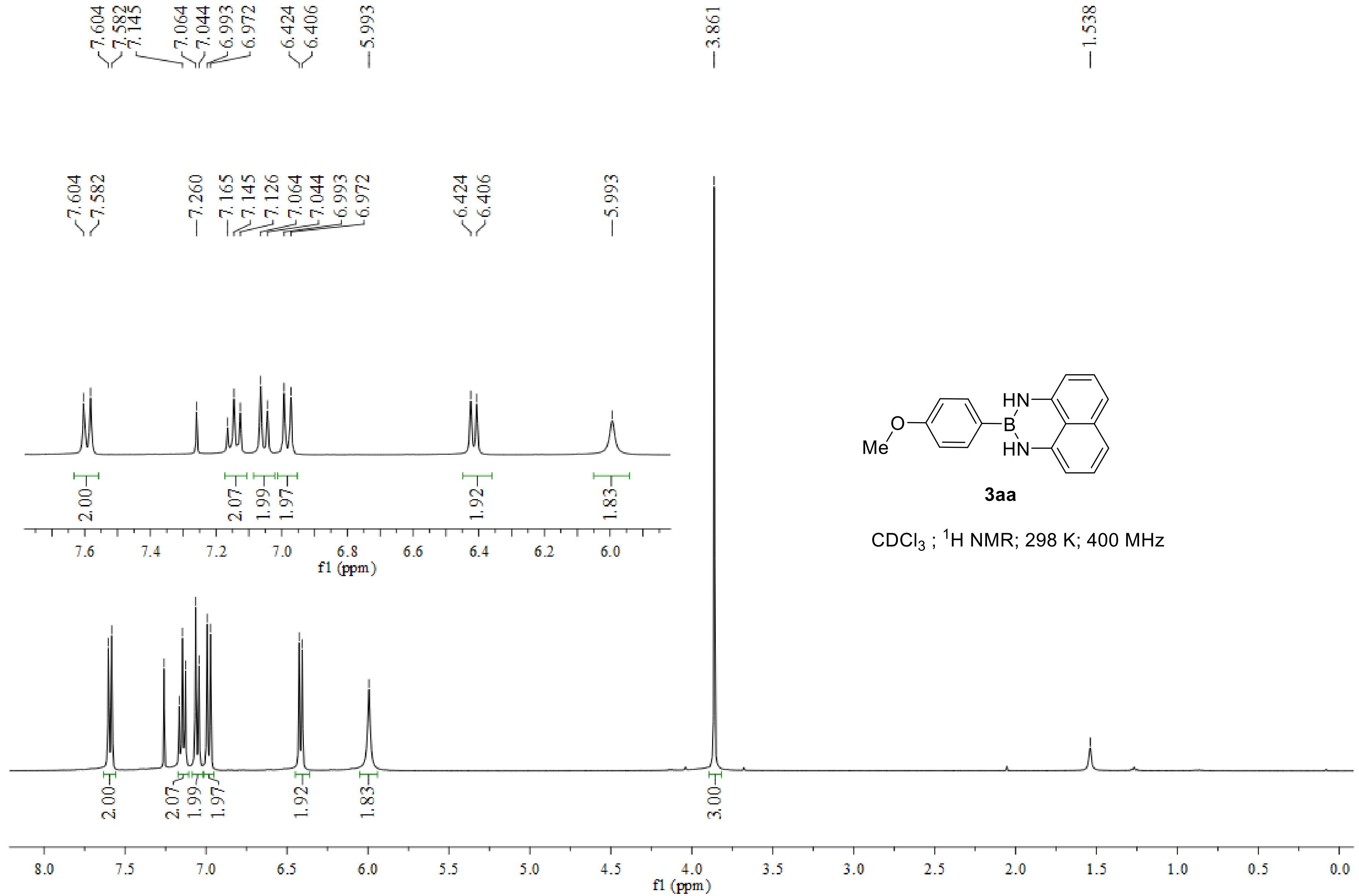

7.604
7.582
7.145
7.064
7.044
6.993
6.972
6.424
6.406
5.993
3.861
1.538
7.604
7.582
7.260
7.165
7.145
7.126
7.064
7.044
6.993
6.972
6.424
6.406
5.993
2.00
2.07
1.99
1.97
1.92
1.83
7.6
7.4
7.2
7.0
6.8
6.6
6.4
6.2
6.0
f1 (ppm)
HN
B
HN
O
Me
3aa
CDCl3 ; 1H NMR; 298 K; 400 MHz
2.00
2.07
1.99
1.97
1.92
1.83
3.00
8.0
7.5
7.0
6.5
6.0
5.5
5.0
4.5
4.0
3.5
3.0
2.5
2.0
1.5
1.0
0.5
0.0
f1 (ppm)

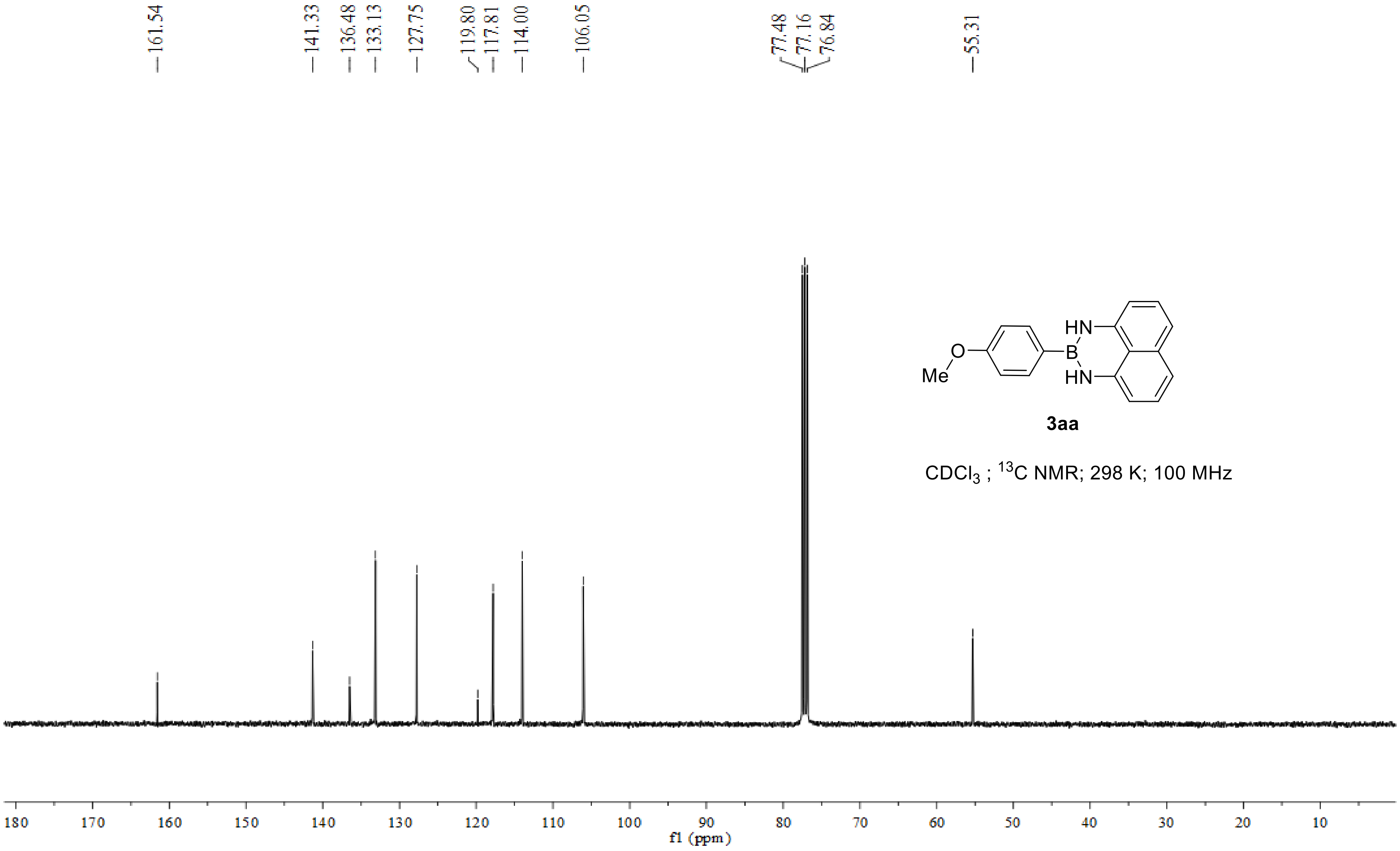
161.54
141.33
136.48
133.13
127.75
119.80
117.81
114.00
106.05
77.48
77.16
76.84
55.31
HN
B
HN
O
Me
3aa
CDCl3 ; 13C NMR; 298 K; 100 MHz
180
170
160
150
140
130
120
110
100
90
80
70
60
50
40
30
20
10
f1 (ppm)

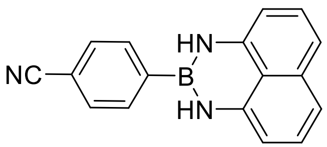

3ab

$CDCl_3$; ^1H NMR; 298 K; 400 MHz

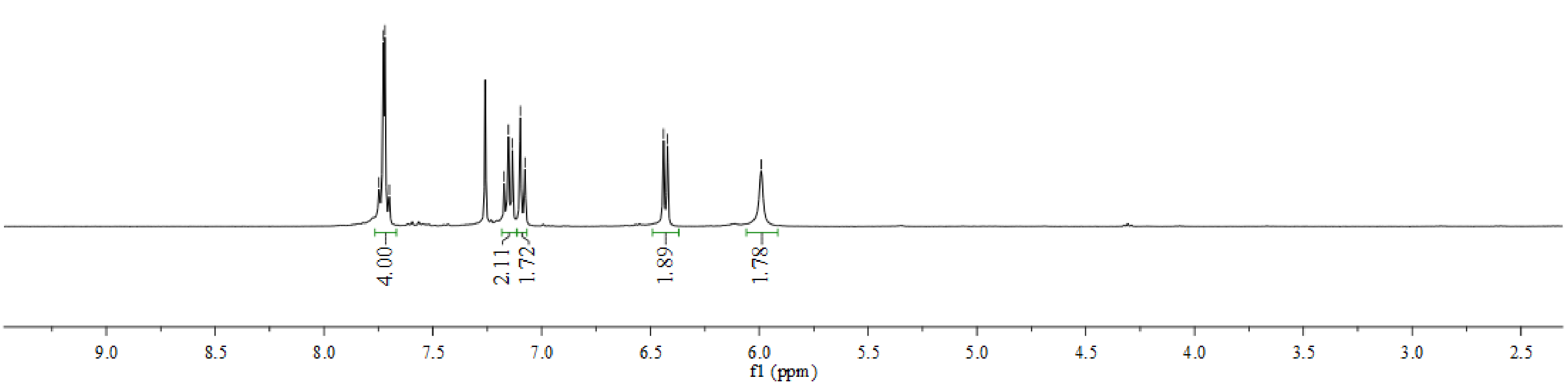

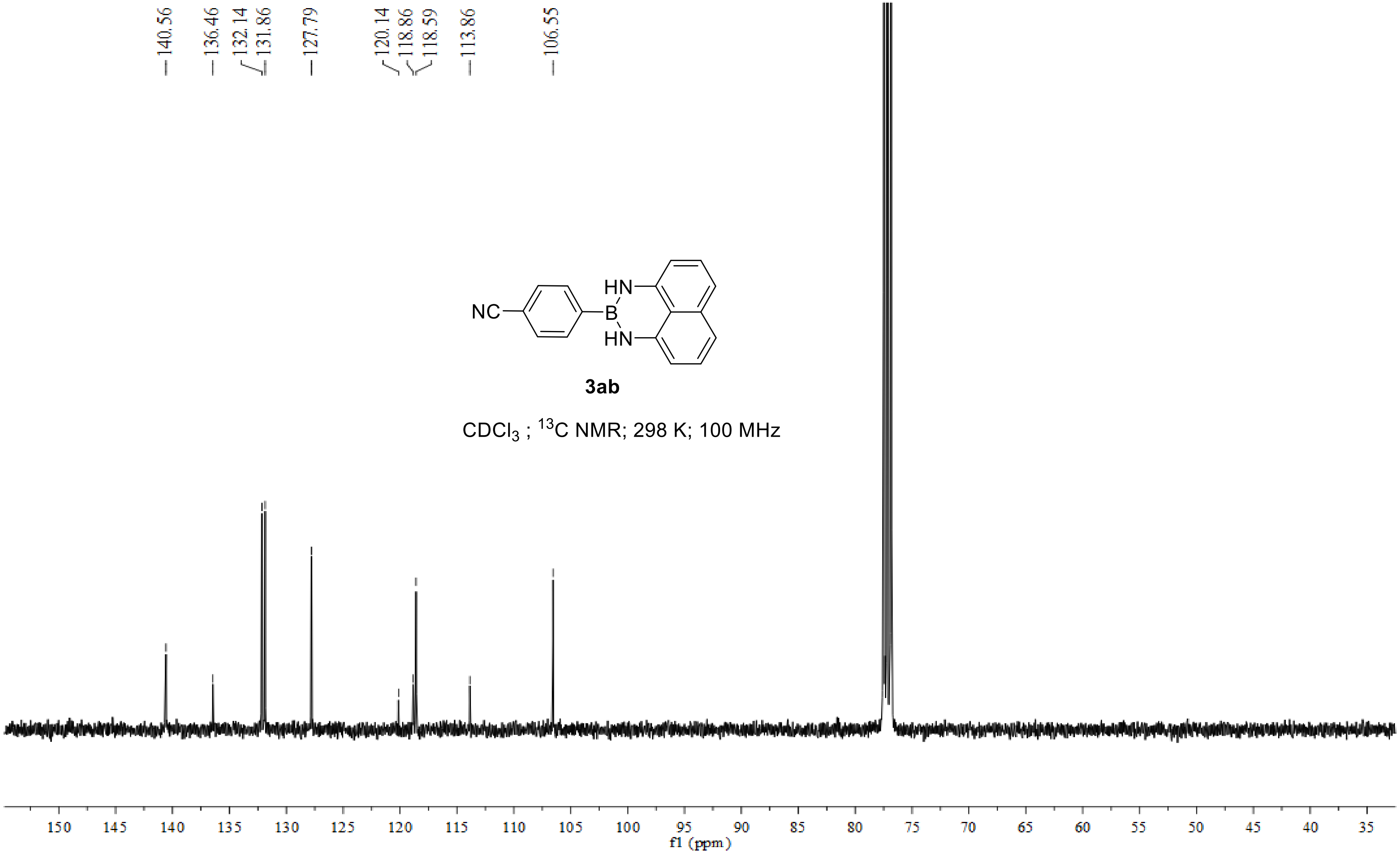
140.56
136.46
132.14
131.86
127.79
120.14
118.86
118.59
113.86
106.55
NC
HN
B
HN
3ab
CDCl3 ; 13C NMR; 298 K; 100 MHz
150
145
140
135
130
125
120
115
110
105
100
95
90
85
80
75
70
65
60
55
50
45
40
35
f1 (ppm)

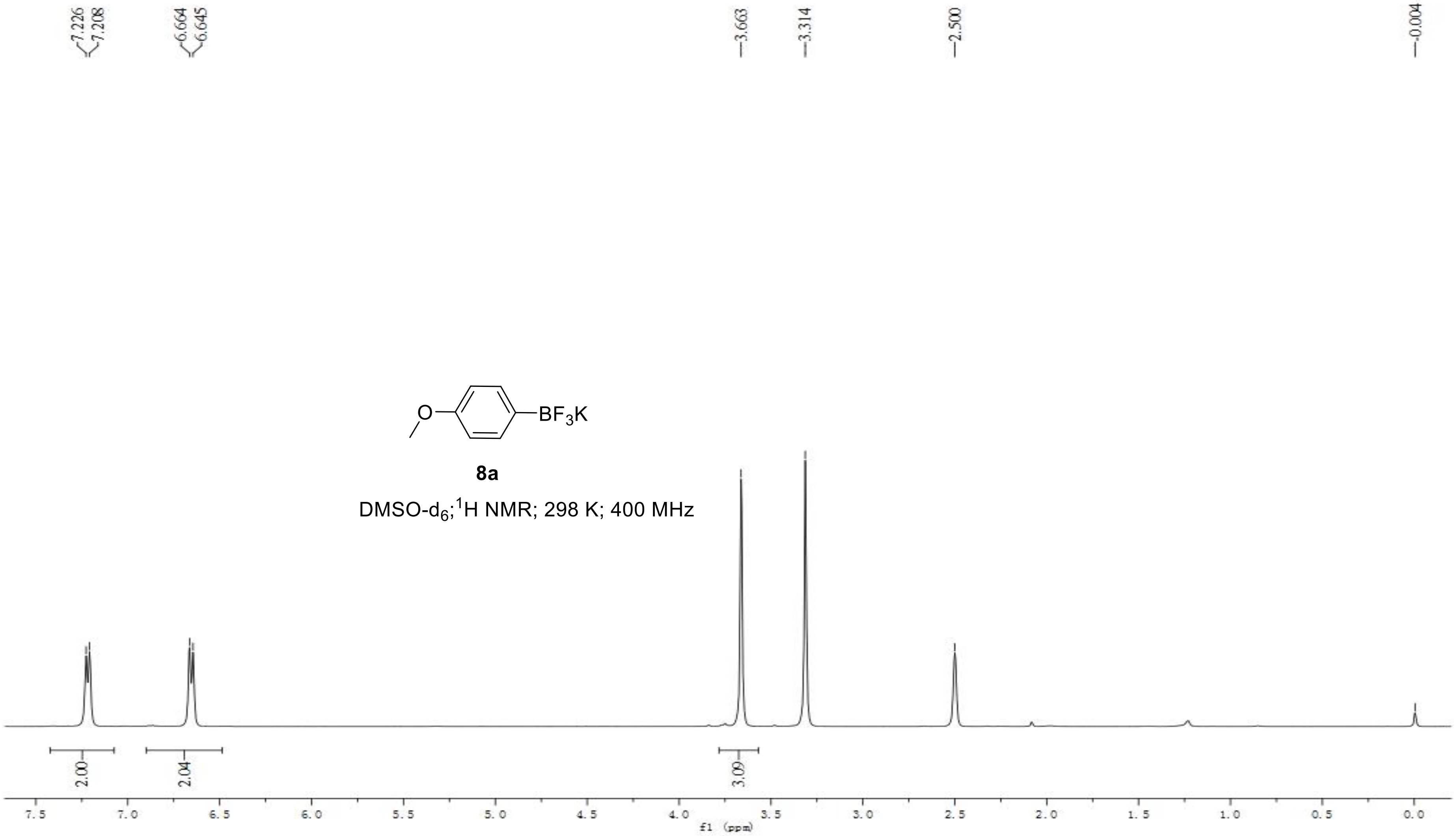
7.226
7.208
6.664
6.645
3.663
3.314
2.500
-0.004
BF3K
O
8a
DMSO-d6;1H NMR; 298 K; 400 MHz
2.00
2.04
3.09
7.5
7.0
6.5
6.0
5.5
5.0
4.5
4.0
3.5
3.0
2.5
2.0
1.5
1.0
0.5
0.0
f1 (ppm)

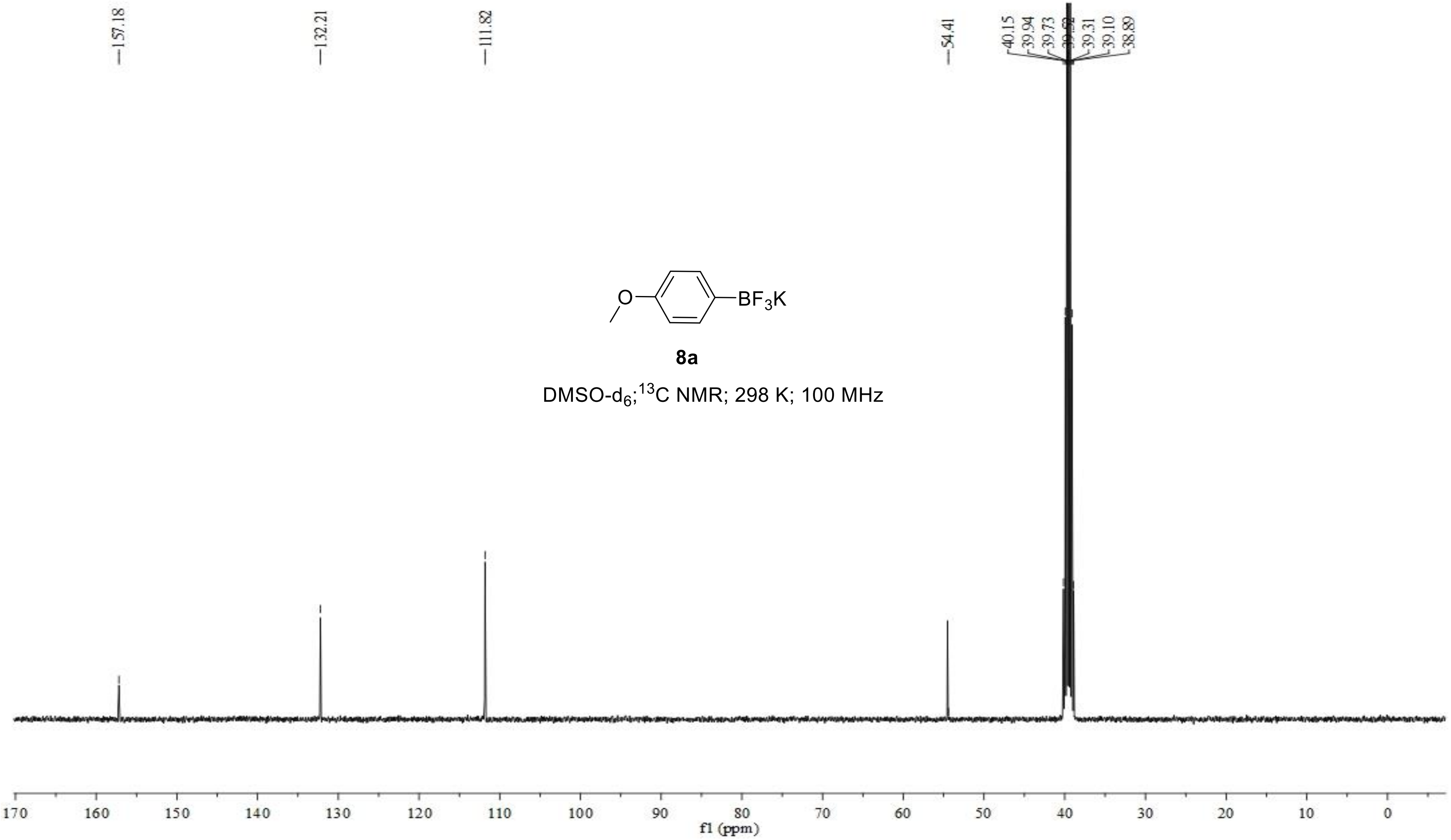

8a

DMSO-d_6; ^{13}C NMR; 298 K; 100 MHz

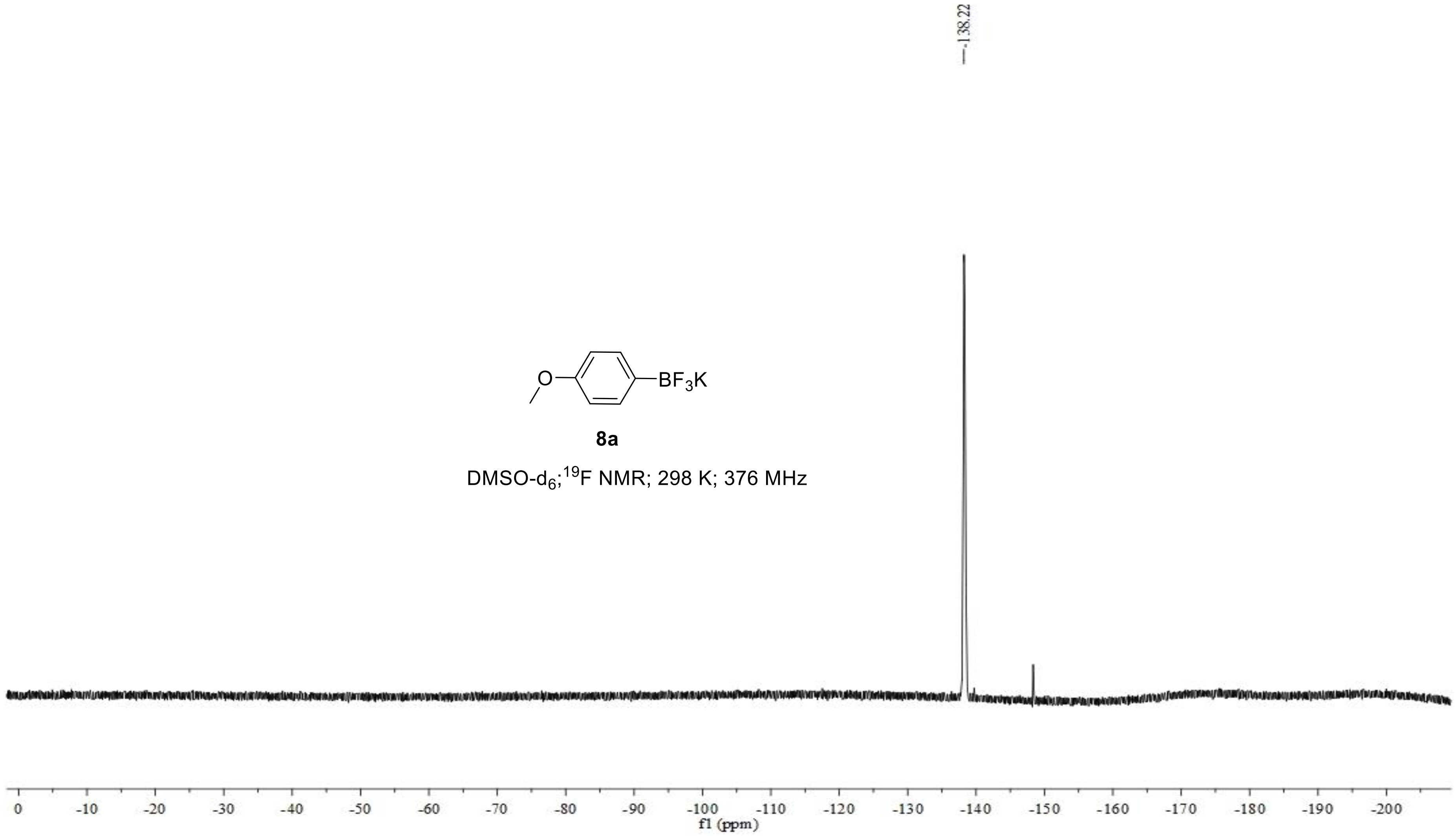

8a

DMSO-d_6;^{19}F NMR; 298 K; 376 MHz

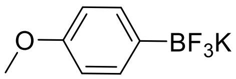

8a

DMSO-d$_6$;^{11}B NMR; 298 K; 128 MHz

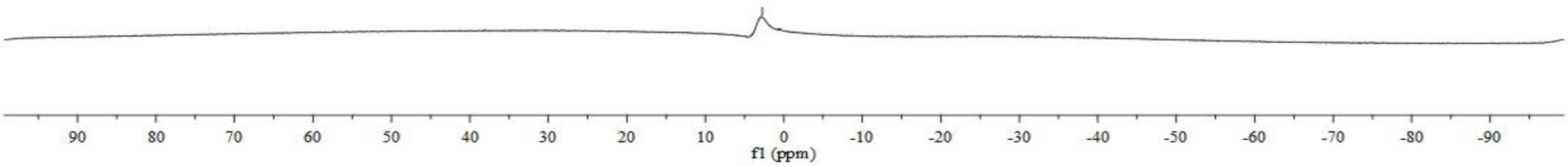

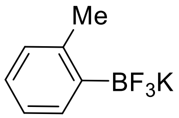

8b

Acetone-d_6; ^1H NMR; 298 K; 400 MHz

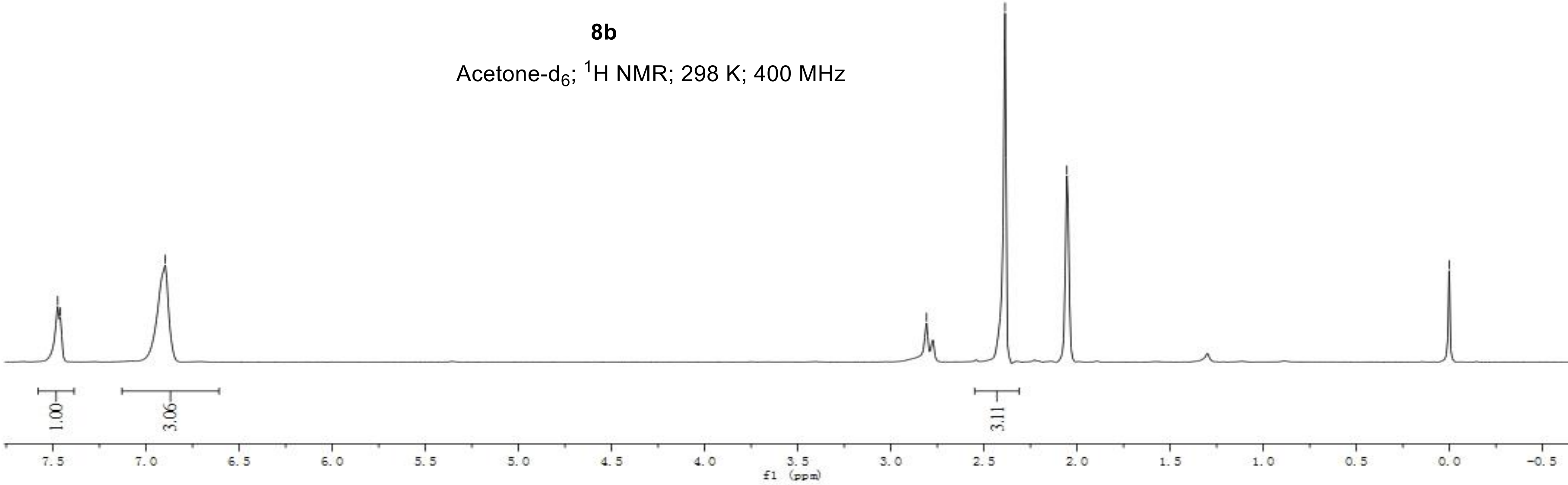

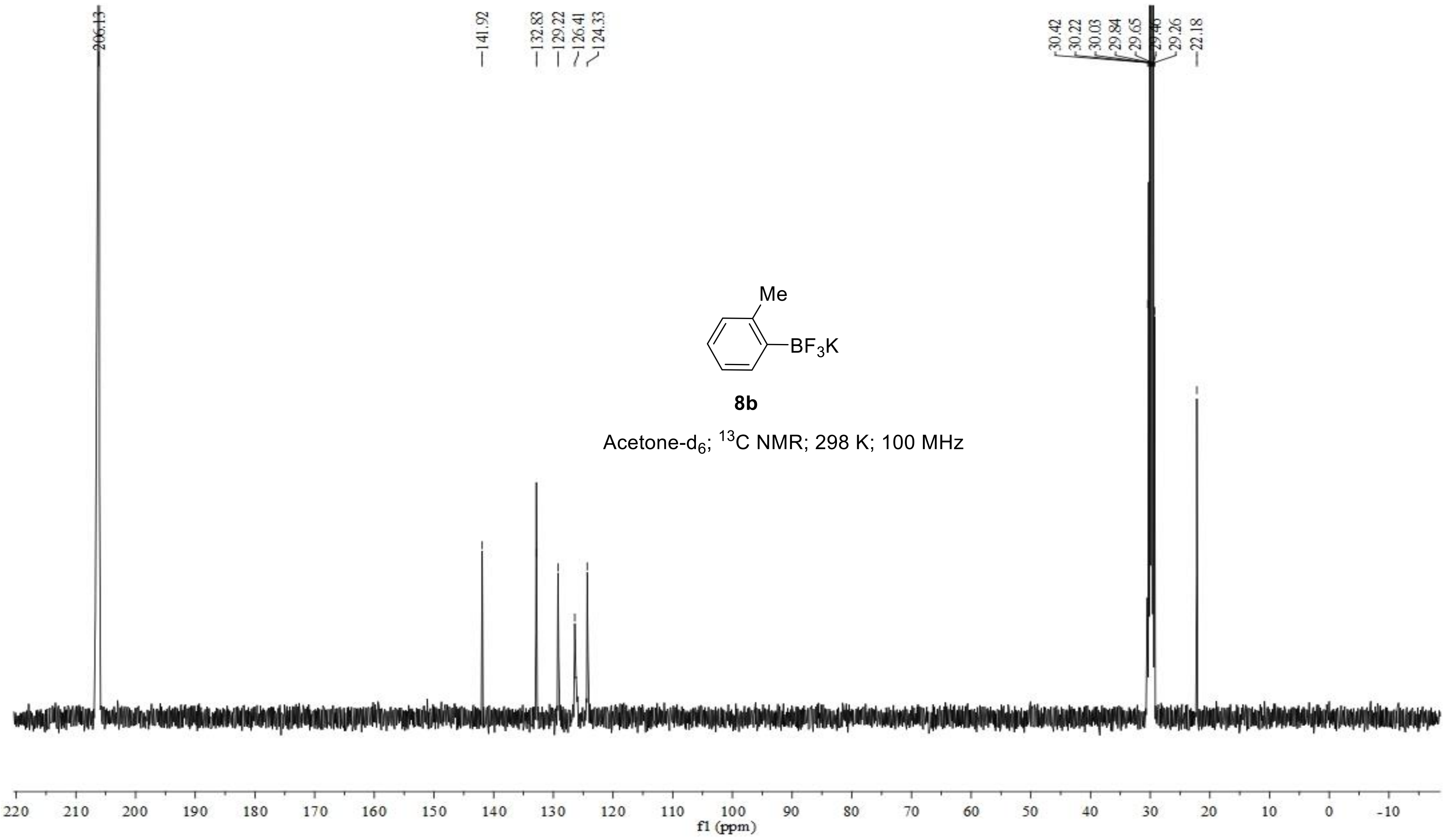
206.13
141.92
132.83
129.22
126.41
124.33
30.42
30.22
30.03
29.84
29.65
29.46
29.26
22.18
Me
BF3K
8b
Acetone-d6; 13C NMR; 298 K; 100 MHz
220 210 200 190 180 170 160 150 140 130 120 110 100 90 80 70 60 50 40 30 20 10 0 -10
f1 (ppm)

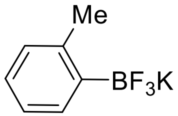

8b

Acetone-d_6; ^{19}F NMR; 298 K; 376 MHz

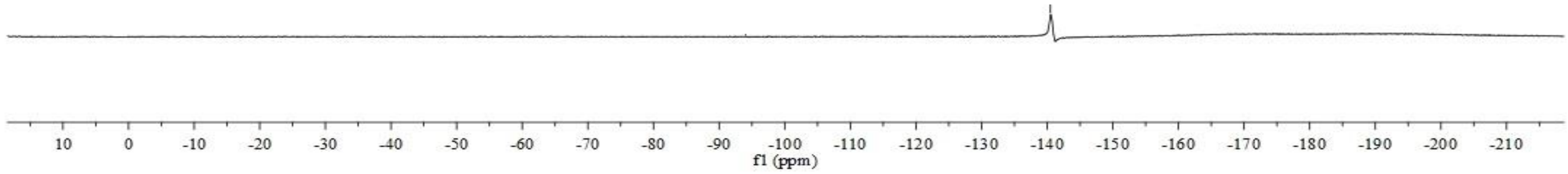

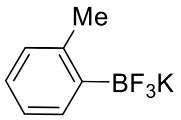

8b

Acetone-d_6; ^{11}B NMR; 298 K; 128 MHz

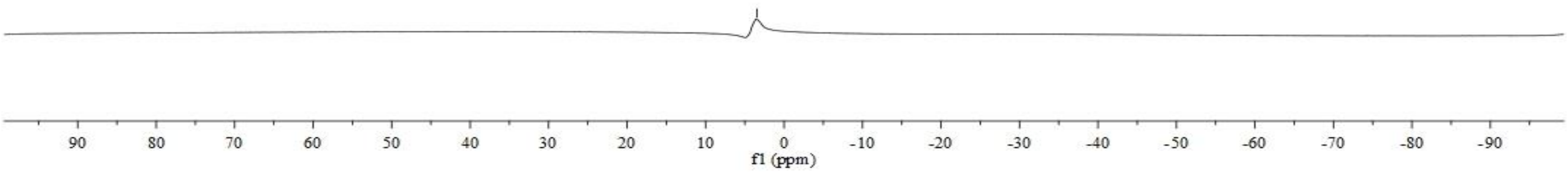

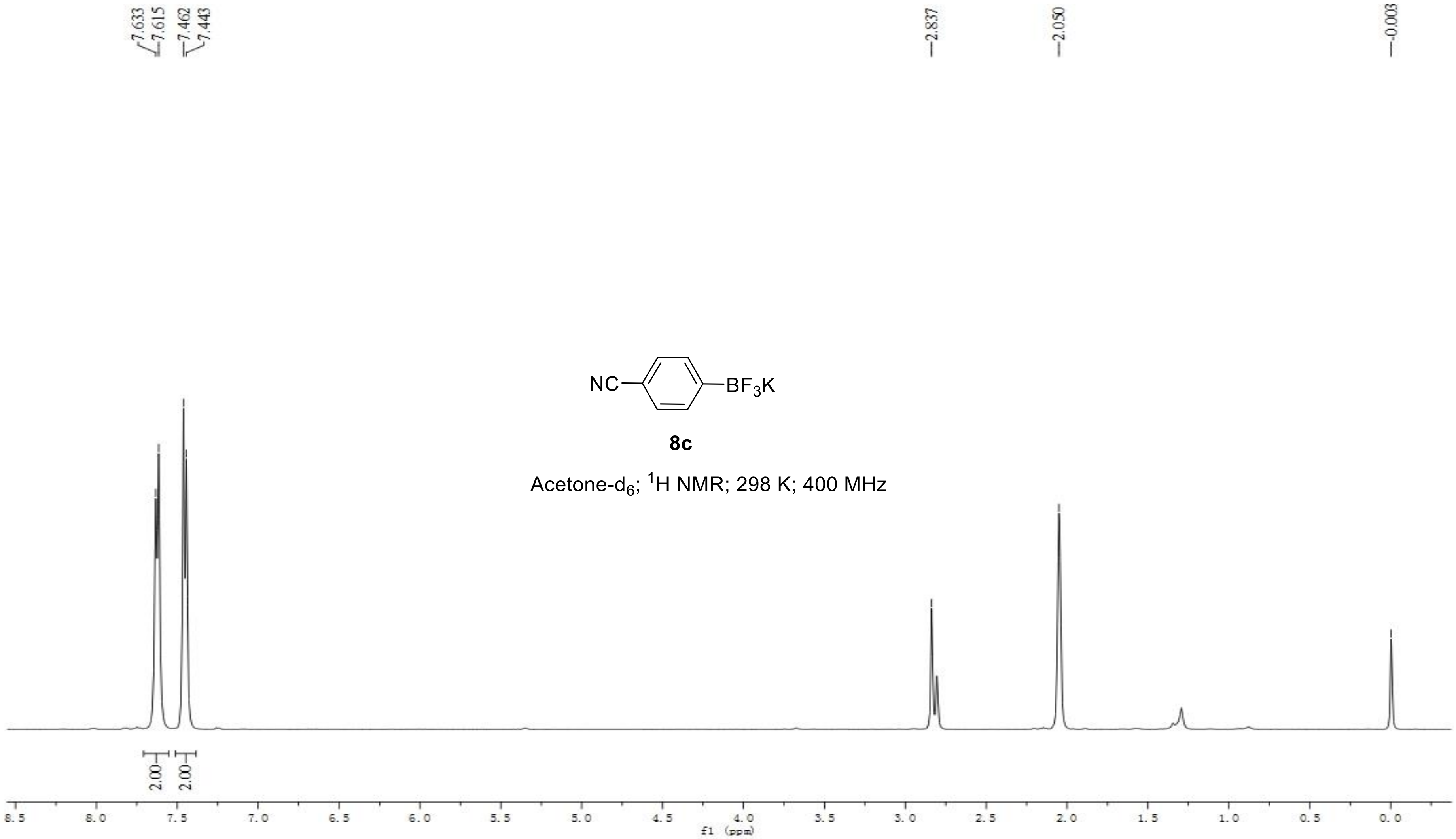

8c

Acetone-d_6; ^{1}H NMR; 298 K; 400 MHz

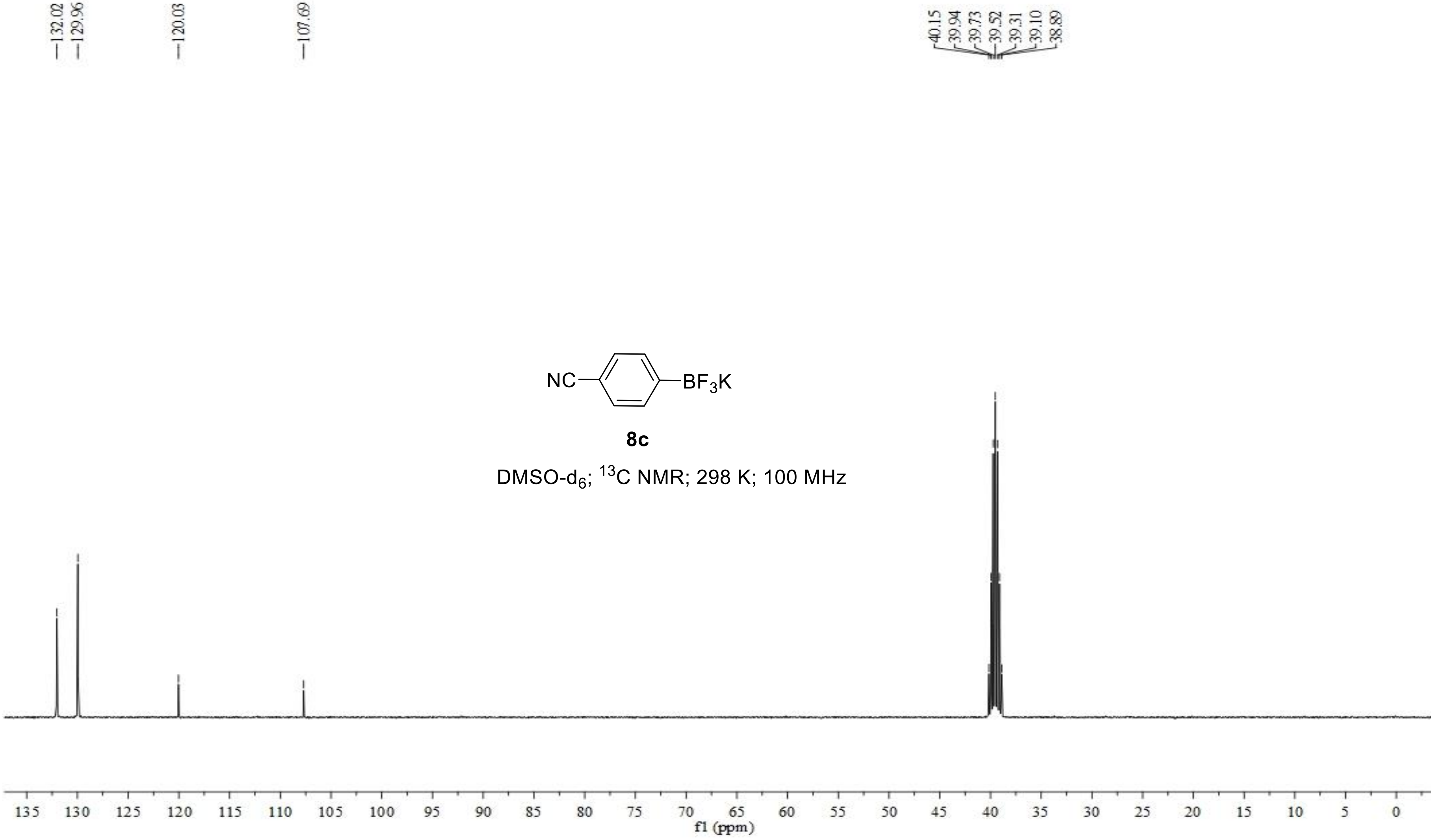

132.02
129.96
120.03
107.69
40.15
39.94
39.73
39.52
39.31
39.10
38.89
NC
BF3K
8c
DMSO-d6; 13C NMR; 298 K; 100 MHz
135 130 125 120 115 110 105 100 95 90 85 80 75 70 65 60 55 50 45 40 35 30 25 20 15 10 5 0
f1 (ppm)

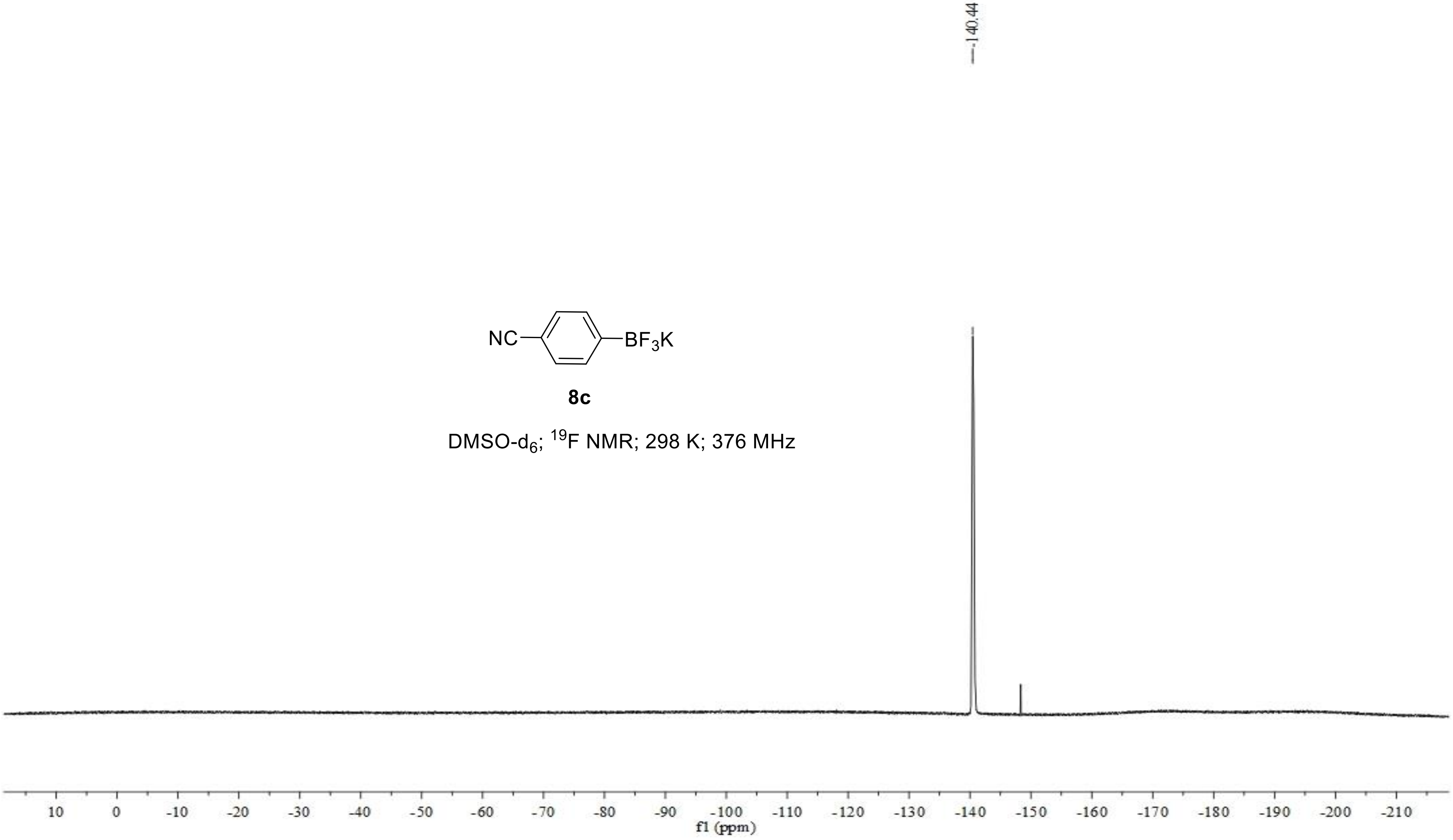
8c
DMSO-d$_6$; ^{19}F NMR; 298 K; 376 MHz
-140.44
10 0 -10 -20 -30 -40 -50 -60 -70 -80 -90 -100 -110 -120 -130 -140 -150 -160 -170 -180 -190 -200 -210
f1 (ppm)

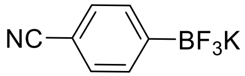

8c

DMSO-d_6; ^{11}B NMR; 298 K; 128 MHz

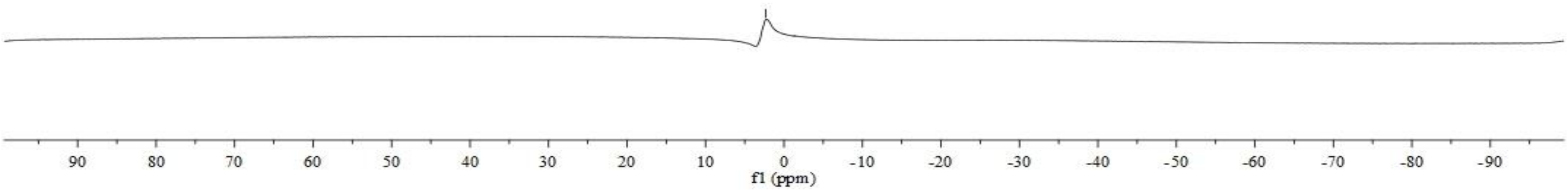

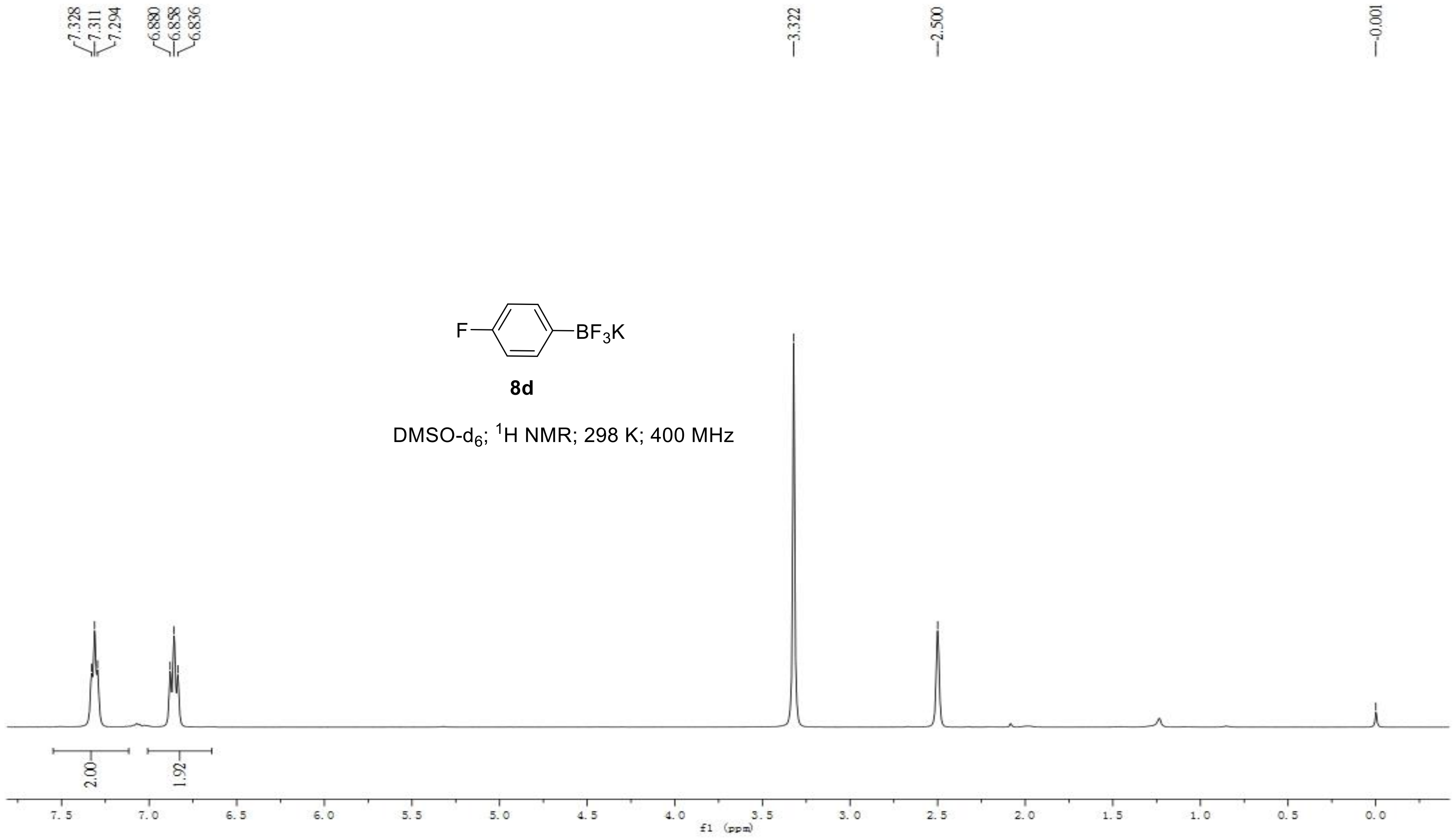
7.328
7.311
7.294
6.880
6.858
6.836
3.322
2.500
-0.001
F
BF3K
8d
DMSO-d6; 1H NMR; 298 K; 400 MHz
2.00
1.92
7.5
7.0
6.5
6.0
5.5
5.0
4.5
4.0
3.5
3.0
2.5
2.0
1.5
1.0
0.5
0.0
f1 (ppm)

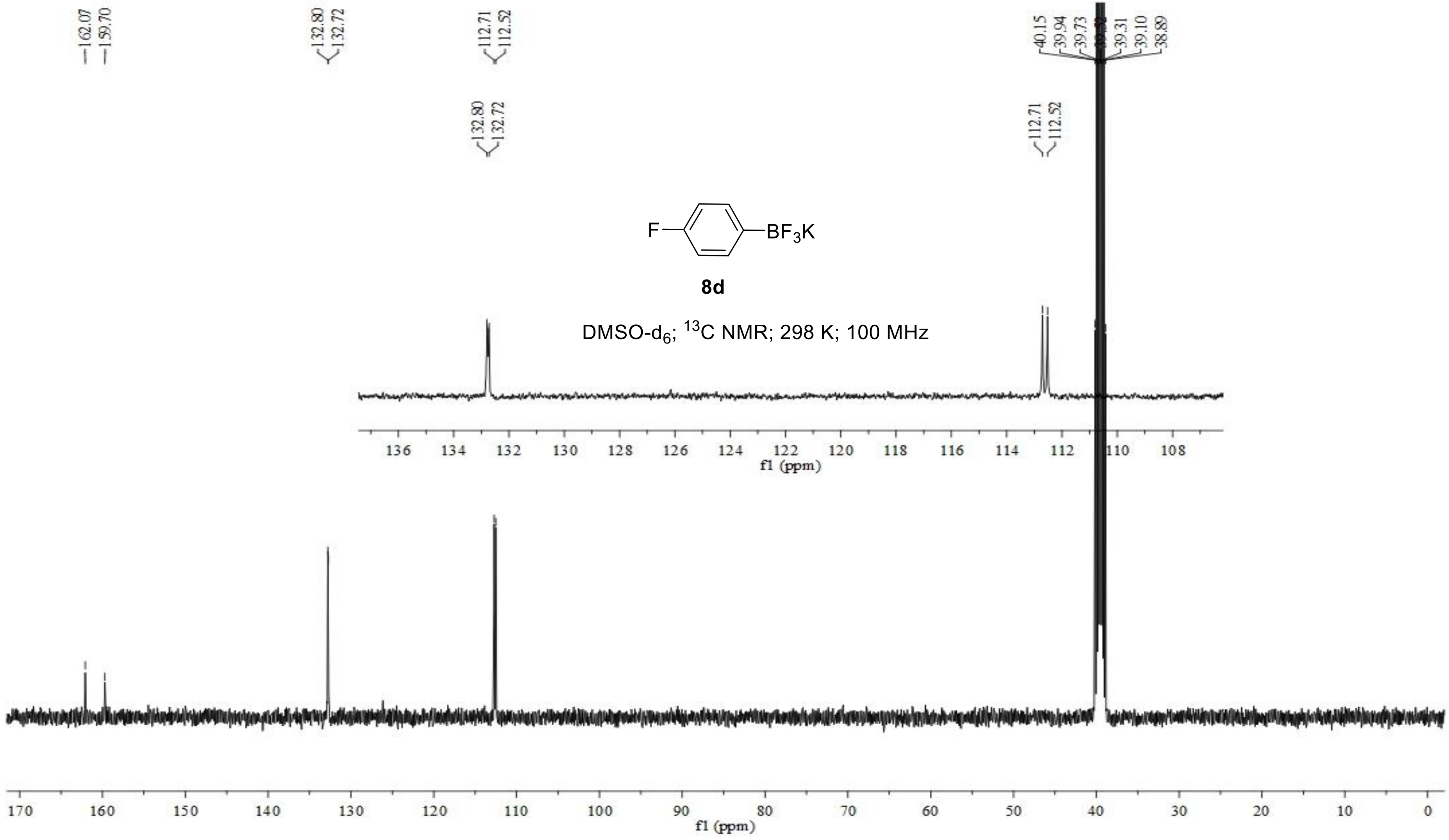
162.07
159.70
132.80
132.72
112.71
112.52
40.15
39.94
39.73
39.31
39.10
38.89
F
BF3K
8d
DMSO-d6; 13C NMR; 298 K; 100 MHz
f1 (ppm)
f1 (ppm)

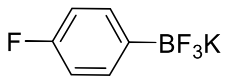

8d

DMSO-d_6; ^{19}F NMR; 298 K; 376 MHz

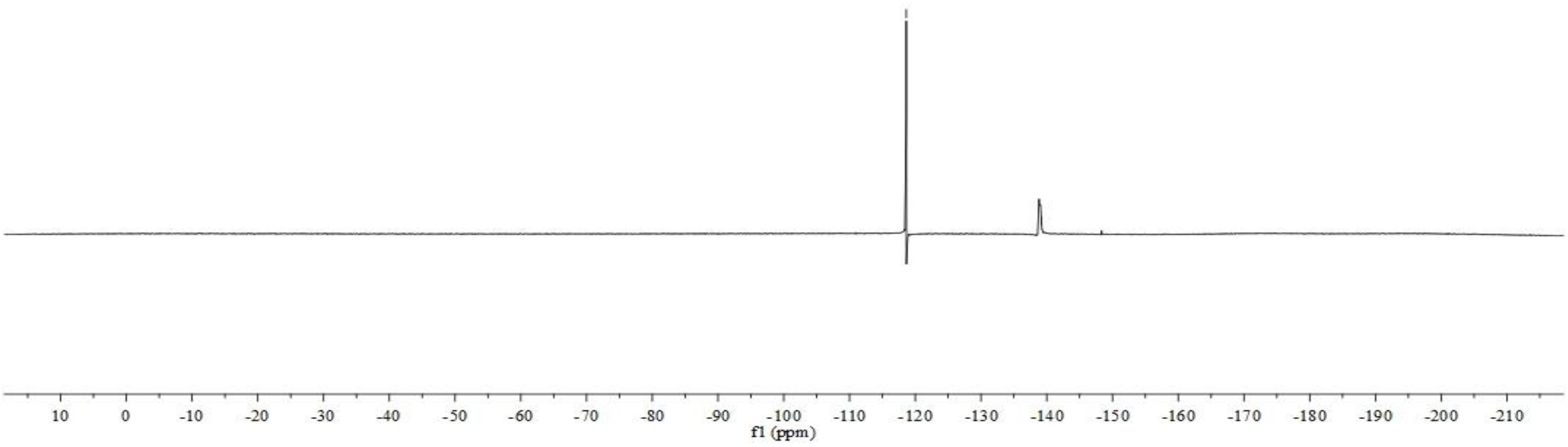

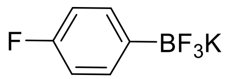

8d

DMSO-d_6; ^{11}B NMR; 298 K; 128 MHz

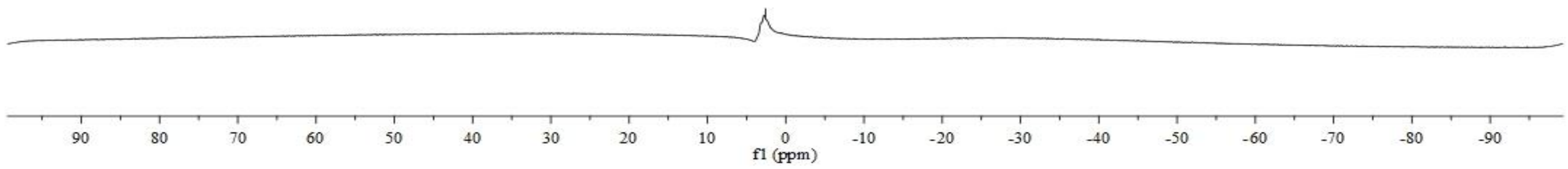

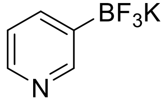

8e

DMSO-d_6; ^{1}H NMR; 298 K; 400 MHz

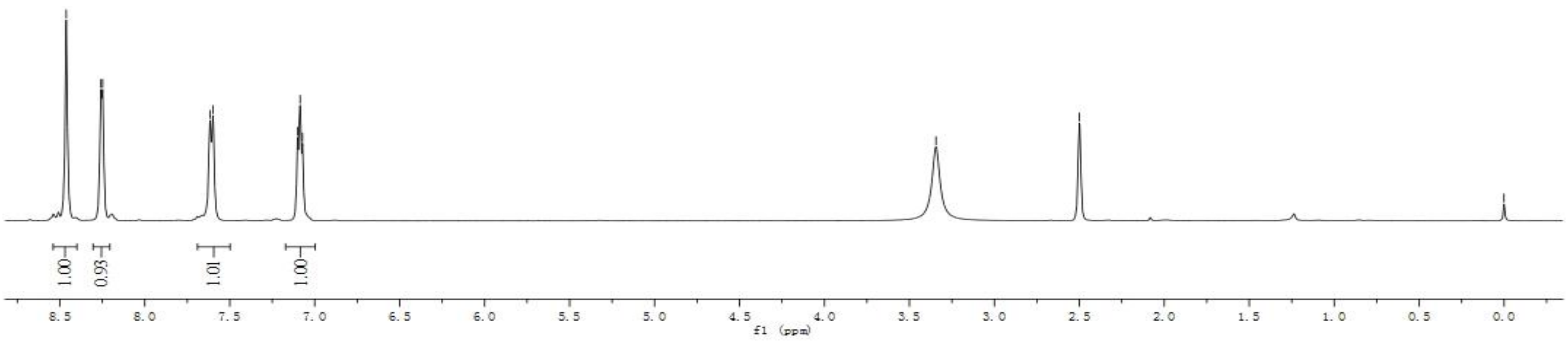

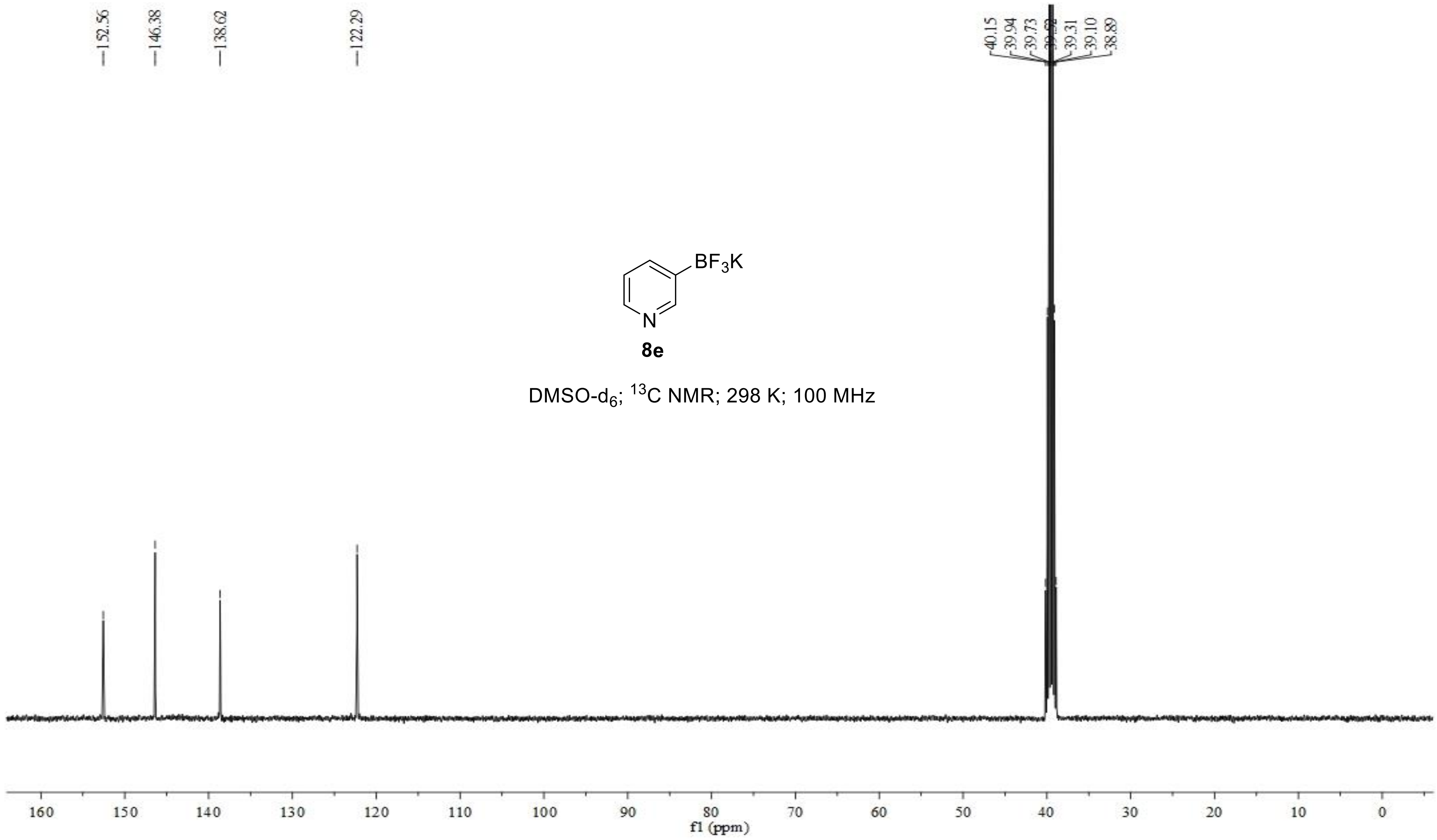
152.56
146.38
138.62
122.29
40.15
39.94
39.73
39.31
39.10
38.89
BF3K
N
8e
DMSO-d6; 13C NMR; 298 K; 100 MHz
160 150 140 130 120 110 100 90 80 70 60 50 40 30 20 10 0
f1 (ppm)

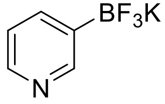

8e

DMSO-d_6; ^{19}F NMR; 298 K; 376 MHz

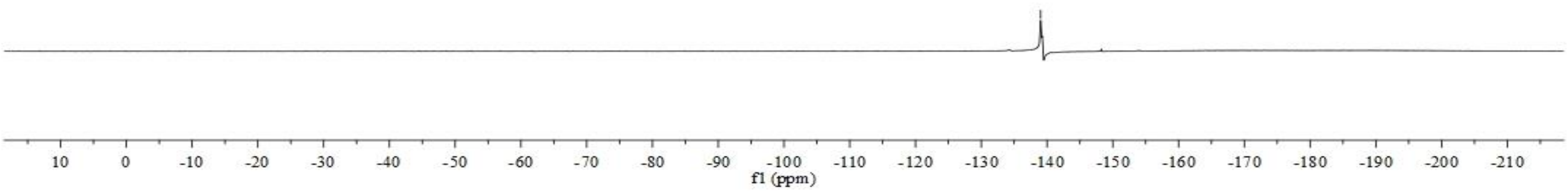

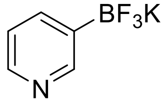

8e

DMSO-d_6; ^{11}B NMR; 298 K; 128 MHz

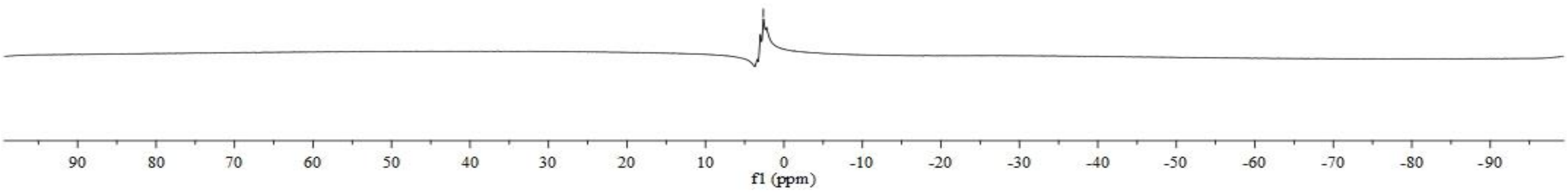

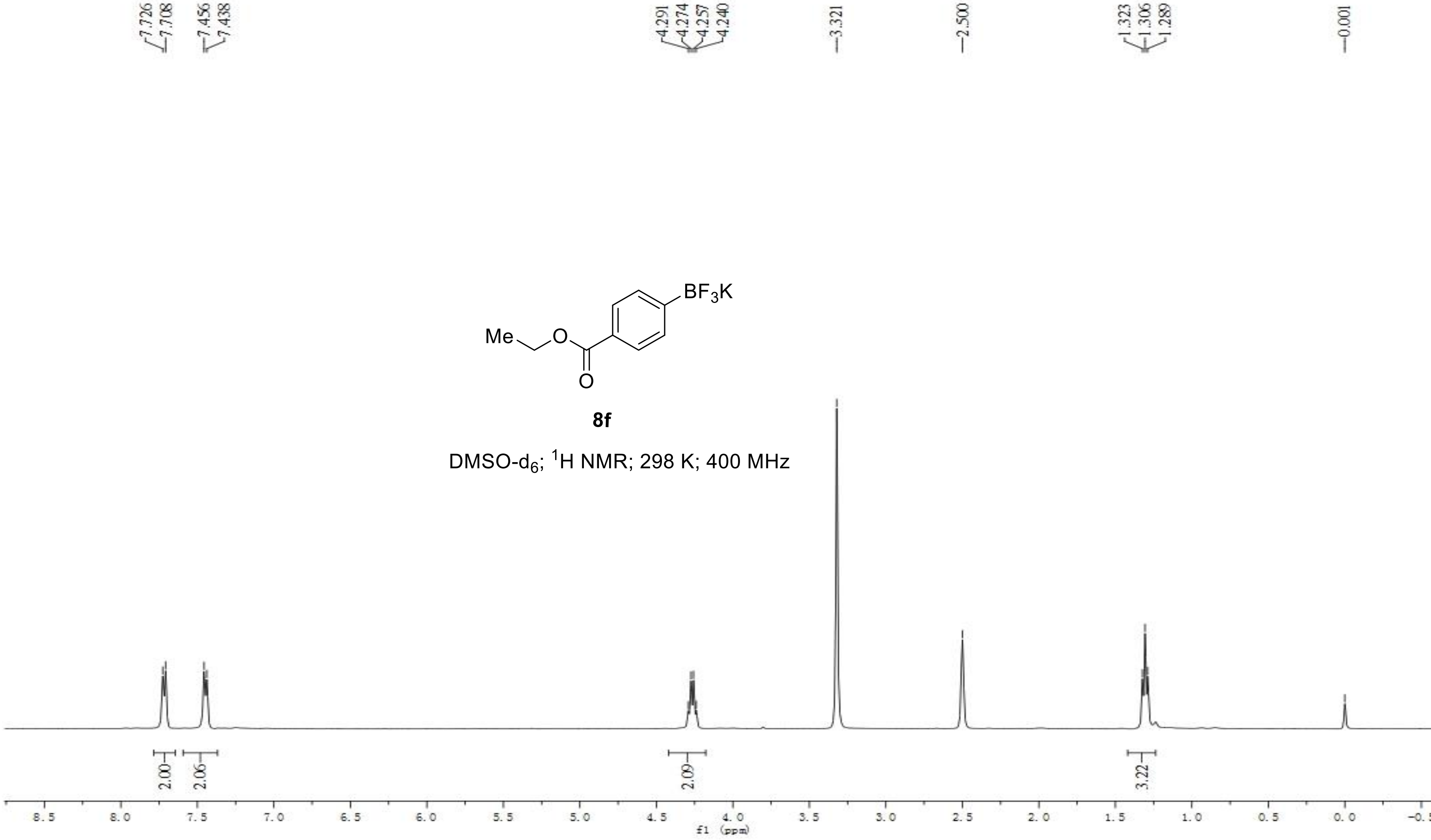
7.726
7.708
7.456
7.438
4.291
4.274
4.257
4.240
3.321
2.500
1.323
1.306
1.289
0.001
BF3K
Me
O
O
8f
DMSO-d6; 1H NMR; 298 K; 400 MHz
2.00
2.06
2.09
3.22
8.5
8.0
7.5
7.0
6.5
6.0
5.5
5.0
4.5
4.0
3.5
3.0
2.5
2.0
1.5
1.0
0.5
0.0
-0.5
f1 (ppm)

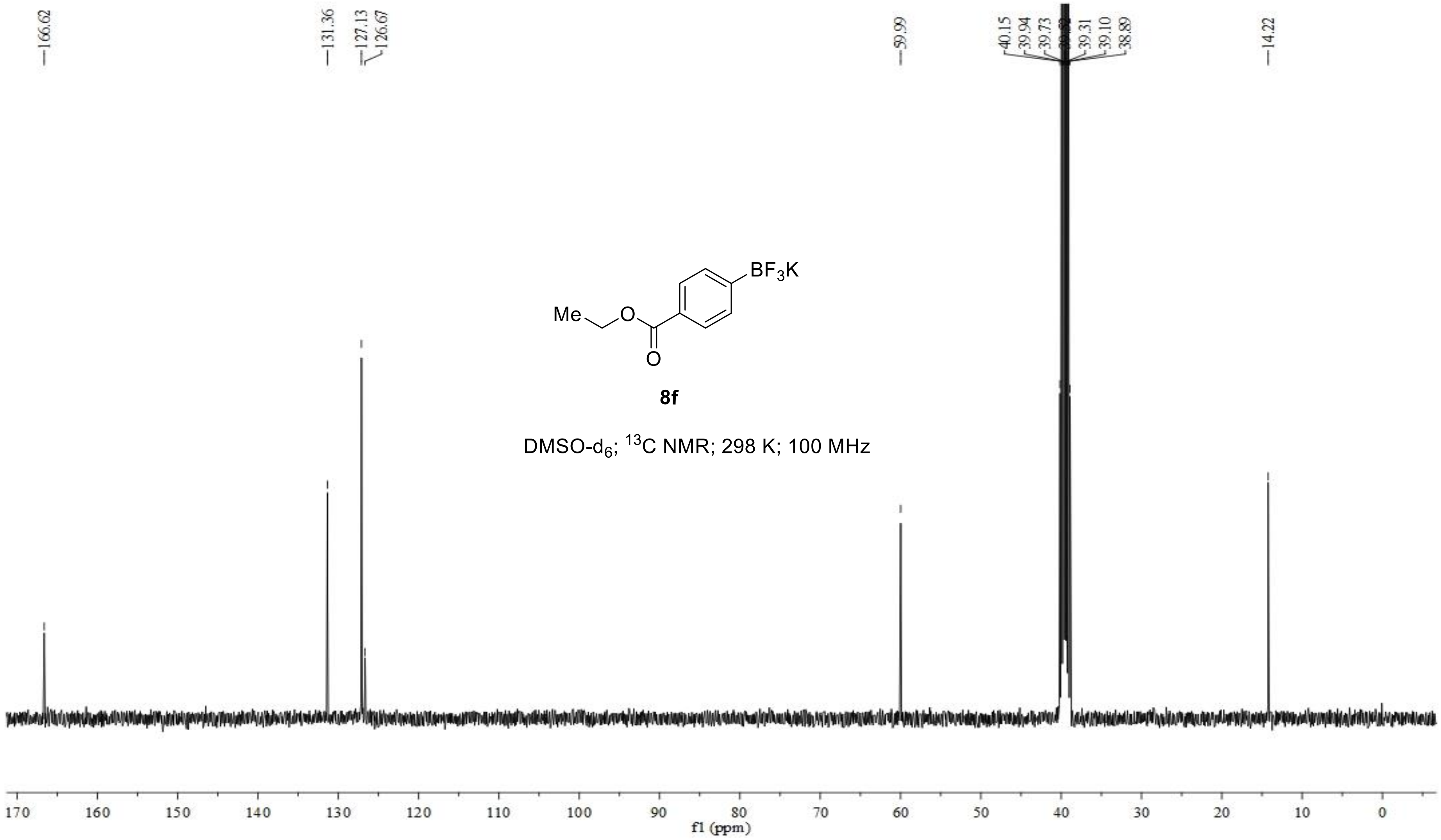

166.62
131.36
127.13
126.67
59.99
40.15
39.94
39.73
39.31
39.10
38.89
14.22
BF3K
Me
O
O
8f
DMSO-d6; 13C NMR; 298 K; 100 MHz
170 160 150 140 130 120 110 100 90 80 70 60 50 40 30 20 10 0
f1 (ppm)

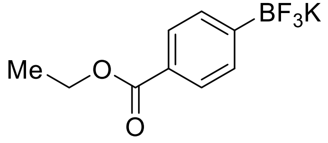

8f

DMSO-d_6; ^{19}F NMR; 298 K; 376 MHz

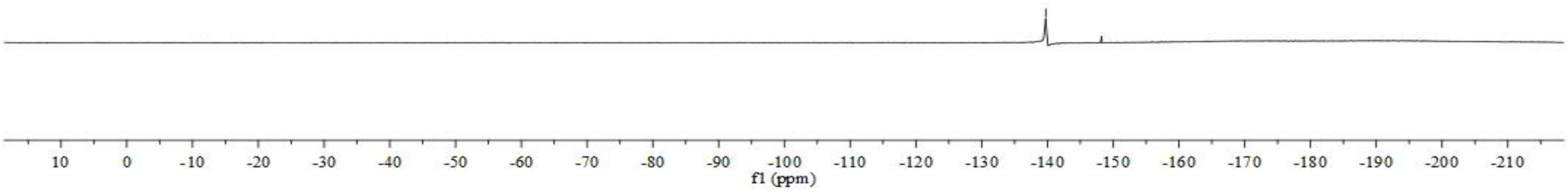

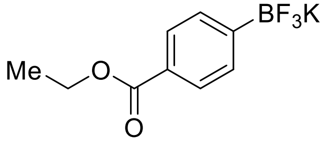

8f

DMSO-d_6; ^{11}B NMR; 298 K; 128 MHz

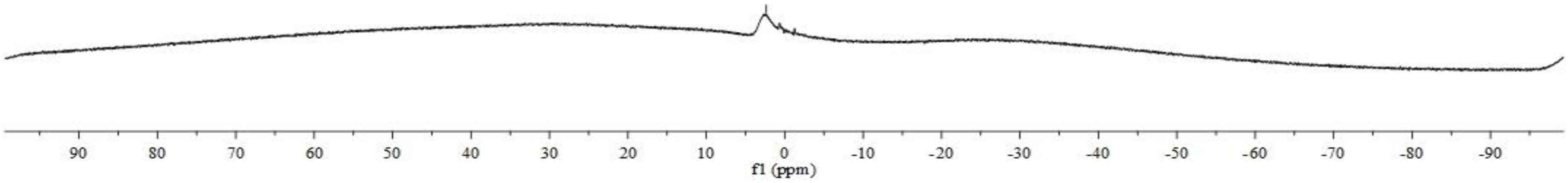

Supplement: Supplementary file 1 [file SC-007-C5SC04521E-s001.pdf]
